# The genetic history of Portugal over the past 5,000 years

**DOI:** 10.1186/s13059-025-03707-2

**Published:** 2025-08-18

**Authors:** Xavier Roca-Rada, Roberta Davidson, Matthew P. Williams, Vanessa Villalba-Mouco, António Faustino Carvalho, Shyamsundar Ravishankar, Evelyn Collen, Christian Haarkötter, Leonard Taufik, Daniel R. Cuesta-Aguirre, Catarina Tente, Álvaro M. Monge Calleja, Rebecca Anne MacRoberts, Linda Melo, Gludhug A. Purnomo, Yassine Souilmi, Raymond Tobler, Eugénia Cunha, Sofia Tereso, Vítor M. J. Matos, Teresa Matos Fernandes, Anne-France Maurer, Ana Maria Silva, Pedro C. Carvalho, Bastien Llamas, João C. Teixeira

**Affiliations:** 1 Australian Centre for Ancient DNA, The Environment Institute, School of Biological Sciences, The University of Adelaide, Adelaide, SA Australia; 2 Faculty of Arts and Humanities, University of Coimbra, Largo da Porta Férrea, Coimbra, 3004-530 Portugal; 3 Department of Biology, Pennsylvania State University, University Park, USA; 4 Institute of Evolutionary Biology, CSIC-Universitat Pompeu Fabra, Barcelona, Spain; 5 Centro de Estudos de Arqueologia, Artes e Ciências do Património - Polo do Algarve, Interdisciplinary Center for Archaeology and Evolution of Human Behaviour, University of Algarve, Faro, Portugal; 6 Technology Advancement Unit, Genetics and Molecular Pathology, SA Pathology, Adelaide, South Australia Australia; 7 Laboratory of Genetic Identification & Human Rights (LABIGEN-UGR), Department of Legal Medicine, Faculty of Medicine, University of Granada, Av. Investigación 11 – PTS – 18016, Granada, Spain; 8 Australian Research Council Centre of Excellence for Australian Biodiversity and Heritage (CABAH), School of Biological Sciences, The University of Adelaide, Adelaide, SA Australia; 9 Mochtar Riady Institute for Nanotechnology, Universitas Pelita Harapan, Tangerang, Indonesia; 10 Research Group in Biological Anthropology (GREAB), Biological Anthropology Unit, Department of Animal Biology, Vegetal Biology and Ecology, Universitat Autònoma de Barcelona, Bellaterra, Spain; 11 Instituto de Estudos Medievais, FCSH-NOVA, Lisboa, Portugal; 12 Research Centre for Anthropology and Health (CIAS), Department of Life Sciences, University of Coimbra, Calçada Martim de Freitas Coimbra, 3000-456 Portugal; 13 HERCULES Laboratory and IN2PAST, University of Évora, Évora, Portugal; 14 National Centre for Indigenous Genomics, John Curtin School of Medical Research, Australian National University, Canberra, Australian Capital Territory, Australia; 15 Indigenous Genomics, The Kids Research Institute Australia, Adelaide, SA Australia; 16 Evolution of Cultural Diversity Initiative, School of Culture, History and Language, The Australian National University, Canberra, ACT, Australia; 17 Australian Research Council Centre of Excellence for Indigenous and Environmental Histories and Futures, Australian National University, Canberra, ACT, Australia; 18 Centre for Functional Ecology, Laboratory of Forensic Anthropology Department of Life Sciences, University of Coimbra, Calçada Martim de Freitas Coimbra, 3000-456 Portugal; 19 National Institute of Legal Medicine, Lisbon, Portugal; 20 School of Sciences and Technology, Department of Biology, University of Évora, Évora, Portugal; 21 Centre for Archaeology, University of Lisbon, Lisbon, Portugal; 22 Centre for Interdisciplinary Studies, University of Coimbra, Coimbra, Portugal; 23 Present Address: Department of Ecology, Evolution, and Organismal Biology, Brown University, Providence, RI USA; 24 Present Address: Center for Computational Molecular Biology, Brown University, Providence, RI USA

**Keywords:** Ancient DNA, Portuguese populations, Iberia, Paleogenomics, Population genetics, Archaeology, Molecular anthropology

## Abstract

**Background:**

Recent ancient DNA studies uncovering large-scale demographic events in Iberia have presented very limited data for Portugal, a country located at the westernmost edge of continental Eurasia. Here, we present the most comprehensive collection of Portuguese ancient genome-wide data, from 67 individuals spanning 5000 years of human history, from the Neolithic to the nineteenth century.

**Results:**

We identify early admixture between local hunter-gatherers and Anatolian-related farmers in Neolithic Portugal, with a northeastern–southwestern gradient of increasing Magdalenian-associated ancestry persistence in Iberia. This profile continues into the Chalcolithic, though Bell Beaker-associated sites reveal Portugal’s first evidence of Steppe-related ancestry. Such ancestry has a broader demographic impact during the Bronze Age, despite continuity of local Chalcolithic genetic ancestry and limited Mediterranean connections. The village of Idanha-a-Velha emerges in the Roman period as a site of significant migration and interaction, presenting a notably diverse genetic profile that includes North African and Eastern Mediterranean ancestries. The Early Medieval period is marked by the arrival of Central European genetic diversity, likely linked to migrations of Germanic tribes, adding to coeval local, African, and Mediterranean influences. The Islamic and Christian Conquest periods show strong genetic continuity in northern Portugal and significant additional African admixture in the south. The latter remains stable during the post-Islamic period, suggesting enduring African influences.

**Conclusions:**

We reveal dynamic patterns of migration in line with cultural exchange across millennia, but also the persistence of local ancestries. Our findings integrate genetic information with historical and archeological data, enhancing our understanding of Iberia’s biological and cultural heritage.

**Supplementary Information:**

The online version contains supplementary material available at 10.1186/s13059-025-03707-2.

## Background

The Iberian Peninsula, located at the westernmost edge of continental Europe, is a geographically *quasi*-isolated region that offers a unique perspective on ancient patterns of human migration into a European *cul-de-sac*. Specifically, this geographical setting provides a valuable opportunity to investigate how relative local isolation and continental patterns of human movement influenced the persistence and addition of genetic ancestries in the region, as well as to investigate the overall structure and stability of local population networks.

The importance of Iberia for European human evolution is underscored by its role as a refugium during the Last Glacial Maximum (LGM, ~ 26,000 to 19,000 years ago), when harsh climatic conditions led to the contraction of human habitable areas across Europe [1–6]. As the climate warmed and the glacial ice receded around 14,000 years ago, hunter-gatherer human populations began to expand across Europe [1–5, 7–11]. This period saw the gradual transition from a nomadic lifestyle to more settled forms of habitats and subsistence strategies in some parts of Europe, as evidenced by Mesolithic necropolises associated with shell middens (i.e., *concheiros*) in present-day Portugal [12]. Previous genetic studies have shown that northwestern, southwestern, and southeastern Iberian Mesolithic hunter-gatherers retained higher ancestry proportions from LGM-related ancestry representative of Magdalenian-associated individuals (Goyet_Q2 cluster) compared to northern and northeastern Iberian Mesolithic hunter-gatherers, which appear to derive most of their ancestry from post-LGM population expansions (Villabruna/Oberkassel cluster) [5, 13–15].

It was not until the beginning of the Neolithic period (~ 5700/5600 BCE) that significant social and demographic changes occurred in Iberia with the introduction of agriculture, herding, and permanent settlements [16]. The shift from hunting and gathering to farming was primarily driven by population movements from Southwestern Asia into Europe [17–21]. Specifically, human groups carrying impressed potteries, such as the Cardial group [16, 22], spread along the Western Mediterranean and reached central-southern Portugal by around 5500 BCE [23, 24]. Recent genetic studies have shown that the spread of Neolithic groups involved dynamic population movements, with admixture between local hunter-gatherers and migrating farmers. As a result, Neolithic Iberian communities retained a higher proportion of hunter-gatherer ancestry than those in Central Europe due to admixture along the migration route and additional Iberian contributions [13, 15, 25–27].

Advancements in metal technology (e.g., copper and gold metallurgy) along with economic and trade intensification resulted in the emergence of more complex forms of social organization during the Chalcolithic period (~ 3000–2000 BCE) [28, 29]. These societies were characterized by diverse regional cultures that, despite interactions, preserved unique cultural traits. Specifically, the Bell Beaker phenomenon in Portugal emerged within preexisting Chalcolithic contexts, contributing to changes in burial practices, material culture, and long-distance connections [28, 29]. Genetic studies indicate a persistence of ancestry from preceding Neolithic populations, while Steppe-related affinities appear sporadically in northern Iberia for the first time [30]. Additionally, a resurgence of hunter-gatherer ancestry is observed in some parts of Europe, likely due to the assimilation of hunter-gatherers that had persisted in certain isolated regions [25, 26, 31, 32].

Changes observed in the Bronze Age (beginning ~ 2200 BCE)—such as the development of bronze metallurgy and the emergence of stronger social stratification—were accompanied by demographic shifts in genetic ancestry influenced by Steppe-related populations in North and Central Europeans and, to some extent, Iberians [13, 30, 31, 33–37]. While the existence of Bronze Age trade networks connecting the Iberian Peninsula with the Mediterranean and Atlantic regions, including North Africa, have been described [38, 39], large-scale genetic evidence of such interactions remains limited, with only sporadic cases detected [13, 27, 37].

During the Iron Age, starting around 800 BCE, the introduction of iron technology significantly advanced agricultural and warfare practices [40]. Several local groups known as *Iberians* occupied much of eastern and central Iberia; Celtic material culture has been found in northern, western, and central regions; while Eastern Mediterranean influences, particularly from the Phoenicians and Greeks, were found in south and east [40]. Beyond these documented cultural exchanges, genetic studies show a slight increase in Steppe-related ancestry in the Iron Age compared to the Bronze Age and Eastern Mediterranean connections in Greek-associated sites [41, 42].

The Roman conquest of the Iberian Peninsula began in the third century BCE in northeastern Iberia. By the mid-second century BCE, Roman forces had gradually extended their control, integrating present-day Portugal into the Roman Empire. The region was initially divided into the provinces of Lusitania and Tarraconensis, with Gallaecia added in the north at a later stage [43]. This period saw urbanization, infrastructure development, and economic growth driven by Mediterranean trade and large-scale mineral exploitation [44]. Roman culture, law, and the Latin language spread throughout the region, gradually integrating local populations, while settlers from various parts of the Empire, including Southern Europe, North Africa, and the Eastern Mediterranean, arrived in Iberia [44]. These demographic changes have been supported both by written records and genetic studies [13, 44, 45].

In the fifth century, the arrival of Germanic tribes contributed to the changing political landscape of the Iberian Peninsula, coinciding with the decline of Roman power [46, 47]. The Suebi dominated much of the northwest, corresponding largely to modern-day northern Portugal, until the Visigoths politically unified the Peninsula in 585 CE [46, 47].

This political unity was later disrupted in the early eighth century, when Islamic tribes from North Africa conquered most of the Iberian Peninsula, incorporating it into the Umayyad Caliphate [48, 49]. The gradual Christian Conquest of present-day Portugal began in the ninth century from the north [50] and culminated in the formation of the County of Portugal and the establishment of the Kingdom of Portugal in 1143 [51]. During the late Middle Ages, the Kingdom of Portugal expanded and consolidated its borders when the Algarve (southern Portugal) was conquered in 1249 [51].

The early Modern Ages (fifteenth to eighteenth centuries) were a period of expansion for Portugal, with the establishment of colonies in Africa, Asia, and South America, and Lisbon emerging as a major global trade hub [52]. The nineteenth century was marked by political instability, including the Napoleonic occupation (1807–1811), the Liberal Revolution of 1820 and a subsequent civil war that lasted until 1834 [52]. The twentieth century saw the monarchy overthrown in 1910, leading to the establishment of the Portuguese Republic, which was followed by Oliveira Salazar’s *Estado Novo* authoritarian regime until the peaceful Carnation Revolution restored democracy in 1974 [52]. Portugal has since become a stable democratic republic, joining the European Union in 1986 and experiencing steady economic and social development [52].

Despite the wealth of archeological and historical evidence gathered to date, the temporal variation of the genetic makeup of Portugal is still poorly characterized, leading to a critically partial understanding of Southwestern European demographic history. Recently, several human ancient DNA studies have aimed to uncover past large-scale demographic events in the history of the Iberian Peninsula [13, 15, 20, 25, 30, 35–37, 45, 53–62], primarily focusing on the study of Spanish populations (*n* = 471 individuals). However, only 51 individual ancient genomes dating from pre-Visigothic times are published for Portugal, which necessarily limits demographic inferences for both earlier and later periods in such a historically rich region [13, 15, 30, 36, 45, 58].

**Fig. 1:**
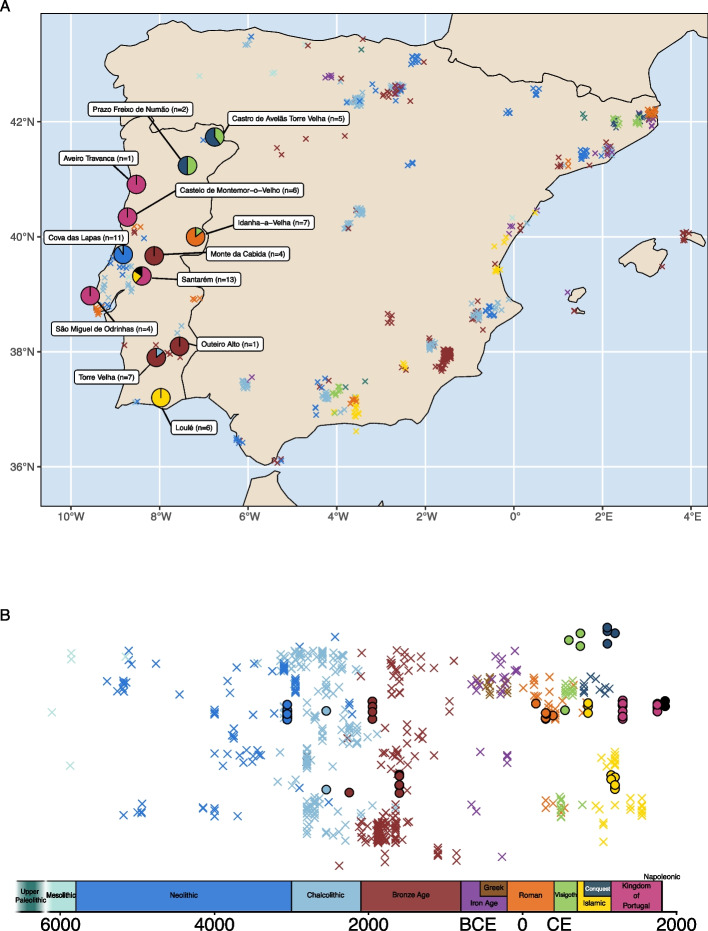
**A** Geographic and **B** chronological distribution of newly generated paleogenomic datasets (*n* = 67; circles) and previously reported paleogenomic datasets (crosses) along a north–south (top to bottom) gradient. Pie charts indicate the archeological sites analyzed in this study, with colors corresponding to different temporal periods. Random jitter has been applied to other sites for clarity

Here, we present the largest collection of ancient Portuguese individuals to date. We screened 94 individuals spanning 5000 years of human history, including newly generated ancient genome-wide data from 67 individuals, from the Neolithic to the nineteenth century (Fig. 1; Additional file 1; Additional file 3: Tables S1-3). Overall, we analyzed 590 Iberian ancient individuals [13, 15, 20, 25, 30, 35–37, 45, 53–62] from key periods of cultural and political transitions in Portugal and Spain—Prehistory, Antiquity, Middle Ages, and Modern Age—to explore population integration and historical movements, thereby enhancing our understanding of the genetic history of Portugal and providing a more complete picture of Iberia’s genetic heritage.

## Results

### Middle/Late Neolithic and Chalcolithic

We analyzed a total of ten newly sequenced individuals from Middle/Late Neolithic Portugal, including eight genetic males and two genetic females (Additional file 3: Table S2) from the archeological site of Cova das Lapas (central-western Portugal), hereafter referred to as Cova_das_Lapas_N. Additionally, we analyzed two new individuals from Chalcolithic Portugal, one from Cova das Lapas (PT_22197; genetically male; hereafter referred to as Cova_das_Lapas_C) and another from Torre Velha 3 (PT_23206; genetically female; southern Portugal; hereafter, referred to as Torre_Velha_C).

We detected a high diversity of mtDNA haplogroups associated with the LGM Iberian refugium (U5b1, U5b1c, U5b1i, U5b2b, U5b2b3a, and U8a1b) and the spread of Neolithic farmers into Europe from Southwestern Asia (K1a4a1, J1c1c, T2b3, and T2c1d) (Additional file 3: Table S5). In contrast, we found only two distinct Y-chromosome sub-haplogroups from the same clade: I2a1a1a1a1 and I2a1a2a/I2a1a2a1a (different levels of resolution within the same sub-haplogroup). Interestingly, the same Y-chromosome sub-haplogroup (I2a1a1a1a) persisted in Cova das Lapas from the Neolithic to the Chalcolithic (Additional file 3: Table S6).

In Cova_das_Lapas_N, we identified a sibling pair between two males and a paternal second-degree relationship between two other males (Additional file 2: Fig. S1; Additional file 3: Table S7). We also found a higher average kinship coefficient among unrelated male pairs compared to unrelated female and male–female pairs (Additional file 3: Table S8). The biological proximity of the first- and second-degree relationships was also observed in a Multidimensional Scaling (MDS) plot based on 1- *f*_3_(Mbuti; Ind1, Ind2) computed iteratively across Neolithic and Chalcolithic individuals from Cova das Lapas (Additional file 2: Fig. S2A). We also noticed a close genetic affinity between Cova_das_Lapas_N and Cova_das_Lapas_C (Additional file 2: Fig. S2A), indicating intra-site genetic continuity across time.

**Fig. 2:**
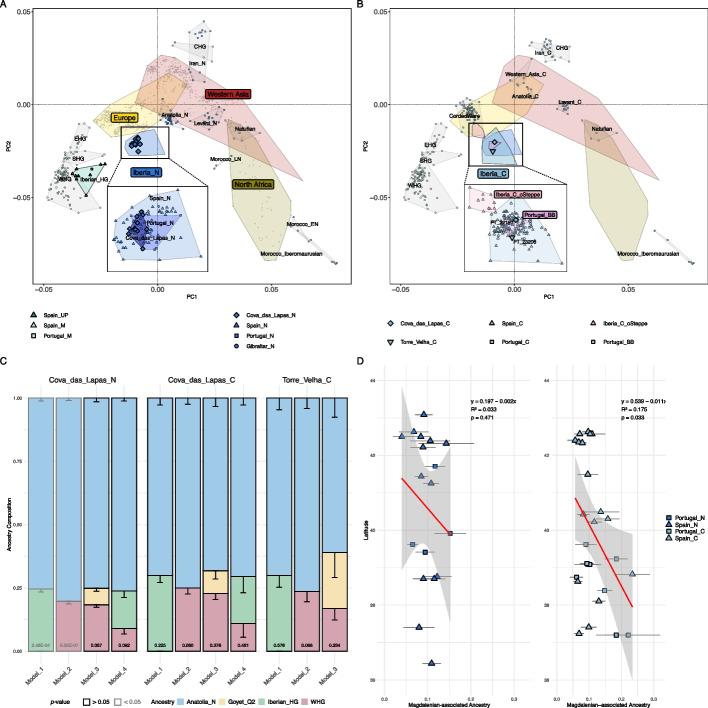
PCA of present-day West Eurasians and North Africans (gray points; overlaid colored polygons represent geographical clusters) with ancient individuals from Iberia and other regions projected onto the first two principal components. Colors indicate different temporal periods as shown in 1B, with a focus on **A** the Neolithic and **B** the Chalcolithic. **C** Ancestry proportions for Cova_das_Lapas_N/C and Torre_Velha_C (*y*-axis) using different admixture modeling frameworks (*x*-axis), *p*-values are provided inside each column and error bars indicate 1-SD. **D** Latitudinal distribution of Magdalenian-associated ancestry in Neolithic and Chalcolithic Iberia. The scatter plot shows the mean proportion of Goyet_Q2 ancestry ± 1-SE for Iberian archeological sites (*x*-axis) plotted against their latitude (*y*-axis). Ancestry proportions derive from *qpAdm* models where Goyet_Q2 was either required as a third source alongside Anatolia_N and WHG or significantly improved model fit. Sites adequately modeled without Goyet_Q2 were excluded from the analysis

Our genome-wide principal component analysis (PCA) computed with present-day West Eurasians and North Africans and projected ancient individuals from these regions revealed that the genetic profile of Cova_das_Lapas_N clusters closely with the broader Iberian Peninsula Neolithic (Fig. 2A). Specifically, Neolithic Iberians fall between pre-Neolithic Iberian hunter-gatherers (Iberian_HG) and Neolithic Anatolian farmers (Anatolia_N). In fact, we observed that Cova_das_Lapas_N and all co-analyzed Neolithic Iberians harbor an admixed genetic profile, comprising both Iberian_HG and Anatolia_N ancestry components, as indicated in *f*_*4*_-statistics and ADMIXTURE (Additional file 2: Figs. S3A, S4). This admixed ancestry was further confirmed through *qpAdm* ancestry modeling (Additional file 3: Tables S11A, S14; Methods), which showed that Cova_das_Lapas_N could be modeled as approximately 76% Anatolia_N, 15% Iberian_HG, and 9% WHG (Luxembourg_Loschbour and Hungary_EN_HG_Koros) (*p* = 0.0922; Fig. 2C), suggesting Cova_das_Lapas_N carried both local hunter-gatherer ancestry and European hunter-gatherer ancestry, likely introduced westward by European farmers.

Interestingly, in the PCA, we also observed that Neolithic Portuguese individuals shifted more towards hunter-gatherer groups compared to the rest of the Iberian Peninsula (Fig. 2A). Outgroup *f*_3_-statistics of the form *f*_3_(Portugal_N/Spain_N, Iberian_HG; Mbuti) suggest that Portugal_N shares more genetic drift with Iberian_HG than Spain_N. While not statistically significant, this pattern indicates a greater persistence of Iberian hunter-gatherer ancestry in Neolithic Portugal (Additional file 2: Fig. S5). This is further supported by *qpAdm* ancestry modeling (Additional file 3: Table S14), which shows a higher hunter-gatherer component (combining WHG and Iberian_HG) in Portugal_N (~ 23.8%) compared to Spain_N (~ 20.7%).

To investigate the Anatolian-related ancestry, we computed outgroup *f*_3_-statistics of the form *f*_3_(Cova_das_Lapas_N/Portugal_N/Spain_N, Test; Mbuti), where *Test* represents two Neolithic populations, one group including individuals from Austria, Czechia, and Germany, representing the Linearbandkeramik (LBK) culture and the Neolithic expansion along the Danube (Danube_LBK_N); and another group containing individuals from Croatia, Italy, and southeastern France, representing the *Impressa* and Cardial Ware cultures and the Neolithic expansion along the Mediterranean (Mediterranean_Impressed_Cardial_N). We observed that Cova_das_Lapas_N, Portugal_N (which includes Cova_das_Lapas_N and previously published Neolithic individuals from Portugal), and Spain_N suggestively exhibit a higher shared genetic drift with Mediterranean_Impressed_Cardial_N than with Danube_LBK_N (Additional file 2: Fig. S6). This result is further supported by significant negative *f*_4_-statistics calculated as *f*_4_(Danube_LBK_N, Mediterranean_Impressed_Cardial_N; Cova_das_Lapas_N/Portugal_N/Spain_N, Mbuti) (Additional file 2: Fig. S7). To determine whether the observed pattern was influenced by the higher hunter-gatherer ancestry in Mediterranean Neolithic groups, we included Poland_GlobularAmphora [63] in the analysis, as this population carries substantial hunter-gatherer ancestry but is primarily associated with the Danube expansion. While *f*_3_-statistics of the form *f*_3_(Poland_GlobularAmphora, Test; Mbuti) showed overlapping standard errors, shared genetic drift remained slightly higher with Mediterranean_Impressed_Cardial_N. Additionally, we found non-significant negative *f*_4_-values, indicating that this test should be interpreted with caution due to potential bias from hunter-gatherer ancestry.

We also found that Cova_das_Lapas_C and Torre_Velha_C cluster together with previously published Neolithic and Chalcolithic Iberians both in PCA and MDS plot (Fig. 2B; Additional file 2: Fig. S2B). In fact, both individuals carry highly similar proportions of hunter-gatherer ancestry (Iberian_HG or WHG) and Anatolian_N ancestry as measured in *qpAdm*, ADMIXTURE, and *f*_*4*_-statistics (Fig. 2C; Additional file 2: Figs. S4, S8A; Additional file 3: Table S12A), suggestive of genetic continuity.

In order to determine the closest genetic affinity between the studied archeological sites and different geographically defined Neolithic and Chalcolithic Iberian groups, we computed outgroup *f*_*3*_-statistics of the form *f*_*3*_(*X*, Test; Mbuti), where *X* represents Cova_das_Lapas_N/C and Torre_Velha_C, and *Test* rotates through distinct Neolithic and Chalcolithic Iberian groups (Additional file 2: Figs. S3B, S8B-C). Interestingly, we found no geographical partition of the genetic data, possibly due to a lack of population structure or insufficient resolution.

To test whether there was a resurgence of hunter-gatherer ancestry in Portugal during the Middle/Late Neolithic and Chalcolithic periods compared to the Early Neolithic as previously suggested in Spain and Europe [31], we performed admixture modeling with *qpAdm* for each Neolithic and Chalcolithic Portuguese archeological site (Additional file 3: Tables S11A-12A) and detected that, on average, the hunter-gatherer component accounted for approximately 28.25% of the genetic profile in Neolithic sites and ~ 29.61% in Chalcolithic sites (Additional file 3: Table S13). This suggests a substantial early contribution of hunter-gatherer ancestry to Early Neolithic Portuguese farmers that persisted over time.

To further investigate the hunter-gatherer ancestry in Neolithic and Chalcolithic Portugal, we computed outgroup *f*_3_-statistics of the form *f*_3_ (X, Test; Mbuti), where *X* includes various archeological sites and *Test* represents different Iberian_HG. Our analysis revealed that most central-western Neolithic sites, including Cova_das_Lapas_N, shared the highest genetic affinity with northwestern Mesolithic individuals from La_Braña, whereas Lugar_do_Canto_N was closest to Los_Canes (Additional file 2: Fig. S9). Both La_Braña and Los_Canes are more closely related to post-LGM hunter-gatherers than Magdalenian-related hunter-gatherers. In contrast, Lorga_de_Din_N (northern Portugal) exhibited greater shared genetic drift with the central Portuguese Late Mesolithic individual Moita_do_Sebastião, which has a genetic ancestry more closely related to Magdalenian-related hunter-gatherers. Conversely, most central Chalcolithic Portuguese sites, along with southern Torre_Velha_C, showed a higher affinity to Moita_do_Sebastião, while others, such as Cova_das_Lapas_C, were more closely related to Los_Canes (Additional file 2: Fig. S10).

To determine whether the LGM Magdalenian-associated ancestry persisted in Neolithic and Chalcolithic Iberia, in particular in Portugal, we computed *f*_4_-statistics of the form *f*_4_(Goyet_Q2, WHG; Test, Mbuti), where *Test* comprised various Neolithic and Chalcolithic Iberian groups as well as Cova_das_Lapas_N/C and Torre_Velha_C (Additional file 2: Fig. S11A-B). All *Test* groups with a high number of SNPs used to compute *f*_4_-statistics yielded significantly negative *f*_4_-values, consistent with WHG admixture. However, we observed a putative and intriguing geographical gradient, with Neolithic and Chalcolithic northern and northeastern groups displaying more negative *f*_4_-values, indicating a stronger affinity to WHG and, by extension, a weaker affinity to Goyet_Q2. Outgroup *f*_3_-statistics indicated that Neolithic and Chalcolithic groups from central, northwestern, western, and southwestern Iberia had a higher shared genetic drift with Goyet_Q2, compared to northern, northeastern, and eastern Iberians (Additional file 2: Fig. S12). This geographical cline is further supported by *f*_4_-statistics of the form (Test, N + NE_Iberia; Goyet_Q2, Mbuti), where *N* + *NE_Iberia* comprises northern and northeastern Iberian groups, and *Test* represents other regional groups (Additional file 2: Fig. S11C-D). Neolithic central, northwestern, and western Iberians show significantly higher affinity to Goyet_Q2, while southwestern (likely due to low coverage) and eastern Iberia exhibit positive but non-significant *f*_4_-values. By the Chalcolithic, however, this pattern becomes regionally restricted, with only southwestern Iberians retaining significantly higher Goyet_Q2 ancestry. Therefore, our analysis suggests a northeastern–southwestern geographical gradient of increasing Goyet_Q2-like ancestry over time, with the lowest persistence in regions near the Pyrenees and the highest in the Atlantic façade.

We further explored the persistence of LGM Magdalenian-associated ancestry by performing admixture modeling using *qpAdm*, including WHG and Goyet_Q2 as putative sources instead of Iberian_HG. In this framework, Goyet_Q2 was required as an additional third component, contributing ~ 7% to the Cova_das_Lapas_N genetic profile (*p* = 0.0873; Fig. 2C; Additional file 3: Table S11A). In contrast, in comparisons involving Cova_das_Lapas_C and Torre_Velha_C, Goyet_Q2 was not required but improved the overall fit. Notably, the models with the largest *p*-values for Cova_das_Lapas_N/C included both WHG and Iberian_HG (Fig. 2C; Additional file 3: Table S12A), but we note the latter also carries Goyet_Q2 ancestry. This observation suggests Neolithic and Chalcolithic populations in Portugal carried both local hunter-gatherer ancestry and European hunter-gatherer ancestry. Interestingly, *qpAdm* modeling indicated a higher Goyet_Q2 component in Torre_Velha_C than Cova_das_Lapas_C (Fig. 2C; Additional file 3: Table S12A), which is consistent with the aforementioned northeastern-southwestern ancestry gradient of greater Goyet_Q2-like affinity (Additional file 2: Figs. S11, S12). This finding is further supported by a negative correlation between latitude and Goyet_Q2 ancestry, indicating a subtle decline in Magdalenian-associated ancestry at higher latitudes in Iberia. This trend was non-significant in the Neolithic (*β* = − 0.002, *p* = 0.471) but became significant in the Chalcolithic (*β* = − 0.011, *p* = 0.033). Particularly, the low R2 (0.175) in Chalcolithic Iberia suggests additional demographic or environmental factors likely influenced this ancestry’s distribution (Fig. 2D; Additional file 3: Tables S11A, S12A). Notably, this spatial pattern was only detectable when analyzing sites where Goyet_Q2 ancestry persisted as a discernible component. Including all Iberian sites—particularly those where Goyet_Q2 was undetectable—removed the statistical significance of the latitudinal cline likely due to noise (Neolithic: *β* = 0.002, *p* = 0.675; Chalcolithic: *β* = − 0.008, *p* = 0.141). The evidence thus suggests that the observed pattern reflects a true latitudinal decline in the persistence of Magdalenian-associated ancestry. Of note, ancestry proportions and model fit in *qpAdm* can be highly variable when calculated from archeological sites represented by single individuals and thus should be interpreted with caution.

When modeling ancestry proportions for different geographically defined Neolithic Iberian groups (Additional file 2: Fig. S13; Additional file 3: Table S11B), we detected the lowest Goyet_Q2 ancestry in northern and northeastern Neolithic Iberia, particularly near the Pyrenees, while the highest proportion (~ 12%) was detected in northwestern Iberia, followed by central Iberia. Additionally, Iran_GanjDareh_N was required as a fourth ancestry source in western Iberia, where Cova_das_Lapas_N is located. The requirement of Iran_GanjDareh_N may not necessarily be indicative of direct ancestry from Neolithic Iran, but rather a signal that could represent a deep ancestral component carried alongside farmer ancestry. This component does not appear consistently in other Neolithic Iberian regions, which may point to either local heterogeneity in early farming groups or later gene flow events in western Iberia. This finding highlights the need for further sampling and analyses in Neolithic Portugal. We then performed admixture modeling for geographically defined Chalcolithic Iberian groups and observed similar patterns of higher Goyet_Q2-like ancestry proportions in southwestern Chalcolithic Iberia (Additional file 2: Fig. S14; Additional file 3: Table S12B). Interestingly, northern and central Iberia required a Steppe-related genetic source represented by Russia_Samara_EBA_Yamnaya as an additional source, while western Iberia could only be modeled with the inclusion of Morocco_Iberomaurusian, suggesting interactions from Steppe-related and African populations, respectively.

**Fig. 3:**
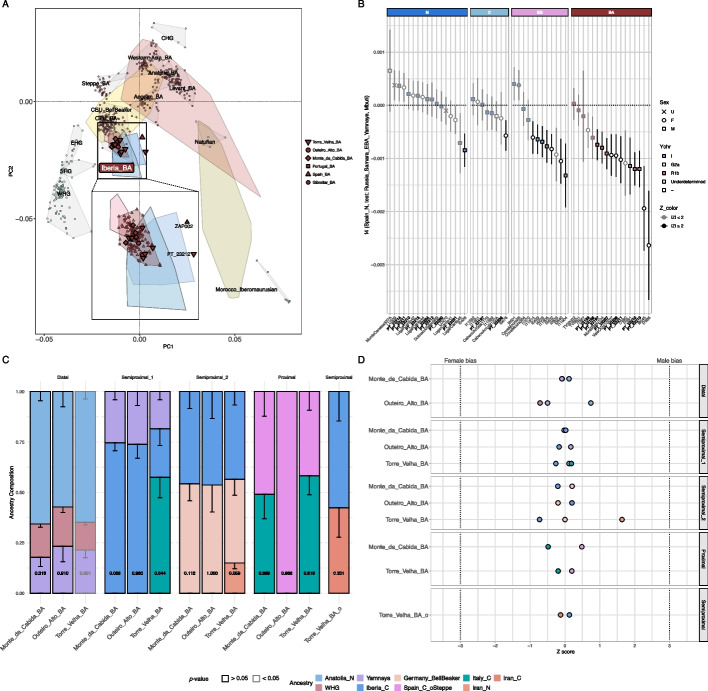
**A** PCA of present-day West Eurasians and North Africans (overlaid colored polygons represent geographical clusters) focusing on the Bronze Age. The ancient individuals from Iberia and other regions were projected onto the first two principal components. Colors correspond to different temporal periods, as shown in 1B. **B** *f*_*4*_-statistics of the form *f*_*4*_ (Spain_N, Test; Russia_Samara_EBA_Yamnaya, Mbuti), where *Test* includes Portuguese individuals from the Neolithic (N), Chalcolithic (C), Chalcolithic Bell Beaker culture (BB), and Bronze Age (BA). Bold labels in the *x*-axis represent individuals from the present study. The *y*-axis shows the *f*_*4*_-statistic values, with results displayed as the mean ± 1-SD. Markers represent the genetic sex, colors indicate Y-chromosome haplogroup lineages, and the black strokes denote Z-scores > 2. **C** Ancestry proportions (*y*-axis) for Monte_da_Cabida_BA, Outeiro_Alto_BA, Torre_Velha_BA and Torre_Velha_BA_o based on different admixture modeling frameworks (*x*-axis), *p*-values are provided inside each column and error bars indicate 1-SD. **D** *qpAdm* Z scores between autosomes and the X chromosome showing no signal for Steppe-related ancestry male bias. If sex bias existed in any of the modeled ancestry sources, we would expect higher (female bias) or lower (male bias) proportions of this ancestry on the X chromosome compared to the autosomes, as the paternal contribution to the X chromosome is only one-third that of the autosomes (fathers contribute an X chromosome exclusively to their daughters). A significantly higher proportion of ancestry on the autosomes would indicate a male-driven contribution

In order to explore the Steppe-related ancestry in Neolithic and Chalcolithic Portugal, we computed *f*_4_-statistics of the form *f*_4_(Spain_N, Test; Russia_Samara_EBA_Yamnaya, Mbuti) (Fig. 3B). Neither individual from Cova_das_Lapas_N/C nor Torre_Velha_C showed significant *f*_4_-values, which was further supported by ADMIXTURE and *qpAdm* analyses (Additional file 2: Fig. S4; Additional file 3: Tables S11A, S14). In contrast, one Neolithic individual displayed barely significant negative *f*_4_-values, likely resulting from an underestimation of the age of the sample. Additionally, most Chalcolithic individuals from archeological sites associated with the Bell Beaker culture showed substantially significant negative *f*_4_-values, which were further supported by ADMIXTURE (Additional file 2: Fig. S4), but not by *qpAdm* (Additional file 3: Table S12B).

To further explore this pattern, we calculated *f*_4_-statistics of the form *f*_4_(Portugal_C, test; Russia_Samara_EBA_Yamnaya, Mbuti), where Portugal_C represents previously reported and newly generated Chalcolithic individuals without Bell Beaker cultural association, and *Test* includes both previously reported and newly generated individuals from Neolithic Portugal (Portugal_N) as well as Bell Beaker-associated Chalcolithic Portugal (Portugal_BB; Additional file 2: Fig. S15). We observed non-significant positive *f*_4_-values for Portugal_N (Z = ~ 1.004) and marginally significant negative *f*_4_-values for Portugal_BB (Z = − 2.884). These results suggest that the earliest presence of Steppe-related ancestry in Portugal occurred during the Chalcolithic at low levels, specifically in Bell Beaker-associated sites, as previously proposed [30]. However, this ancestry had a limited demographic impact, as further evidenced by PCA (Fig. 2B), ADMIXTURE (Additional file 2: Fig. S4), and *qpAdm* ancestry modeling for Portugal_C and Portugal_BB (Additional file 3: Table S14), as well as the persistence of local uniparental markers (Additional file 3: Tables S5-6), and an MDS plot based on 1 – *f*_3_(Mbuti; Ind1, Ind2) which includes Chalcolithic and Bronze Age Iberians (Additional file 2: Fig. S16C).

#### Bronze age

A total of eleven ancient individuals (genetically identified as seven males and four females; Additional file 3: Table S2) were newly sequenced from three southern Portuguese Bronze Age archeological sites, including Monte da Cabida 3 (*n* = 4), Torre Velha 3 (*n* = 6), and Outeiro Alto (*n* = 1), hereafter referred to as Monte_da_Cabida_BA, Torre_Velha_BA, and Outeiro_Alto_BA, respectively. Of note, two individuals from Monte_da_Cabida_BA were previously published [64], but here we provide new sequencing data from the same individuals using the Twist Bioscience “Twist Ancient DNA” reagent kit (Additional file 2: Fig. S17; Additional file 1).

A parent–offspring relationship between a female-male pair from Torre_Velha_BA (PT_23208 and PT_23207) was identified (Additional file 2: Fig. S17-18; Additional file 3: Table S9), both sharing the same mtDNA haplogroup X2c2 (Additional file 3: Table S5). This pair clusters separately from the rest of the population in the MDS plot based on 1-* f*_*3*_(Mbuti; Ind1, Ind2) (Additional file 2: Fig. S16A). Additionally, this analysis revealed that individuals from the Chalcolithic and Bronze Age periods sampled at Torre Velha 3 cluster together, suggesting population continuity across time.

Genome-wide analysis showed that Monte_da_Cabida_BA, Torre_Velha_BA, and Outeiro_Alto_BA cluster with previously published Bronze Age Iberians (Iberia_BA), coinciding with a shift of BA towards Bronze Age Central Europeans in the PCA (Fig. 3A). In addition, we observed geographical population structure in Bronze Age Iberia, with archeological sites in close proximity exhibit higher shared genetic drift, computed by outgroup *f*_*3*_-statistics of the form *f*_*3*_(*X*, Test; Mbuti), where *X* represents the sites, and *Test* rotates through distinct Bronze Age Iberian groups (Additional file 2: Fig. S19).

This shift observed in PCA aligns with the presence of Steppe-related ancestry across the three studied sites as per ADMIXTURE analysis (Additional file 2: Fig. S4), which is further supported by significantly negative *f*_4_-statistics of the form *f*_4_(Spain_N, Test; Russia_Samara_EBA_Yamnaya, Mbuti) (Fig. 3B). Notably, we detected a higher Steppe-related affinity in the newly analyzed individuals from Torre_Velha_BA and Monte_da_Cabida_BA compared to individuals from the same archeological sites previously published [13, 36].

Steppe-related ancestry patterns were consistent across the Iberian Peninsula and showed a broad demographic impact, as evidenced by ADMIXTURE (Additional file 2: Fig. S4), *f*_*4*_-statistics of form *f*_*4*_(Portugal_C, Portugal_BA; Russia_Samara_EBA_Yamnaya, Mbuti) (Additional file 2: Fig. S15), *qpAdm* (Additional file 3: Table S14), and an MDS plot based on 1- *f*_3_(Mbuti; Ind1, Ind2) with Chalcolithic and Bronze Age Iberians (Additional file 2: Fig. S16B). However, we observed a slight decrease towards the south and southwest of Iberia, supported by *f*_*4*_-ratios (Additional file 2: Fig. S20). Moreover, compared to many mainland European populations, individuals from Iberia—particularly those from Portugal—exhibit relatively low affinities to Steppe-related ancestry, as indicated by *f*_3_-statistics. This pattern is comparable to that observed in other western European groups (Additional file 2: Fig. S21). These results suggest a diffusion route of Steppe-related ancestry across Iberia, with the south and southwest of the Peninsula corresponding to the latest point of arrival.

This Bronze Age ancestry shift coincides with a turnover in Y-chromosome R1b lineages in Portugal (Fig. 3B; Additional file 3: Table S6). In addition, we also detected a persistence of mtDNA haplogroups associated with the LGM Iberian refugium and the spread of Neolithic farmers into Europe from Southwestern Asia (Additional file 3: Table S5), but none of the most frequent Eastern European mtDNA haplogroups. Importantly, despite these observations, we found no evidence of sex bias migration of Steppe-related ancestry (Fig. 3D; Additional file 3: Table S18), and thus no indication of a male-driven genetic transition [13], as previously suggested [37].

To investigate the genetic profiles of Bronze Age Portuguese sites, we implemented a series of ancestry modeling frameworks that incorporate source populations with increasing degrees of temporal and geographic proximity. Firstly, we performed a distal framework aimed at testing whether the genetic profiles could be explained solely with WHG/Iberian_HG and Anatolia_N or whether Russia_Samara_EBA_Yamnaya was required as an additional source. The genetic profile of the previously published site of Casas_Velhas_BA (southwestern Portugal) could be explained solely by WHG and Anatolia_N (*p* = 0.8085). While the inclusion of Russia_Samara_EBA_Yamnaya as an additional source was not required for Outeiro_Alto_BA (*p* = 0.1408) and Gruta_do_Medronhal_BA (central Portugal; *p* = 0.3739), it substantially improved the model fit for both sites, contributing to ~ 23% (*p* = *0.9098*) and ~ 40% (*p* = 0.9874), respectively. Furthermore, Russia_Samara_EBA_Yamnaya was required as a third ancestry component for Monte_da_Cabida_BA (~ 18%; *p* = 0.3156) and all other previously published sites (~ 19%). Notably, this distal model did not fit Torre_Velha_BA (*p* < 0.05*;* Fig. 3C; Additional file 3: Table S15).

To better capture more regional patterns of admixture, we implemented semiproximal models that used more temporally and geographically closer source populations. In semiproximal framework 1 (Semiproximal_1), we modeled genetic profiles using Iberia_C and Russia_Samara_EBA_Yamnaya to evaluate whether the Steppe-related ancestry was transmitted through intermediary groups already present in Chalcolithic Iberia. This framework explained the genetic ancestry of Outeiro_Alto_BA (*p* = 0.9799), Monte_da_Cabida_BA (*p* = 0.0676), and all the previously published sites. Notably, the model fit for Monte_do_Vale_do_Ouro_2_BA and Monte_da_Cabida_BA improved when including an additional African or Mediterranean source, respectively (Fig. 3C; Additional file 3: Table S16).

A second semiproximal framework (Semiproximal_2) assessed the contribution of Central European populations in Iberia by including Iberia_C and Germany_BellBeaker—representing groups with documented Steppe-related ancestry that may have contributed to Iberian Bronze Age gene pools through Bell Beaker-mediated migration. This framework also explained the genetic ancestry of Outeiro_Alto_BA (*p* = 0.9996), Monte_da_Cabida_BA (*p* = 0.1122) and Monte_do_Gato_de_Cima_3_BA (southern Portugal; *p* = 0.5843), but not that of Gruta_do_Medronhal_BA (central Portugal; *p* < 0.05) and Monte_do_Vale_do_Ouro_2_BA (southern Portugal; *p* < 0.05). As in Semiproximal_1, the model fit for Monte_da_Cabida_BA improved when including an additional Mediterranean source (Fig. 3C; Additional file 3: Table S16). Notably, Casas_Velhas_BA could be solely explained with Iberia_C (*p* = 0.6212), without requiring any Steppe-related ancestry in either semiproximal framework (Additional file 3: Table S16).

In both semiproximal frameworks, Torre_Velha_BA remained an outlier, with none of the two-way models providing adequate fits. We therefore implemented three-way models that added Mediterranean or North African sources to account for more complex admixture histories (Fig. 3C; Additional file 3: Table S16). Torre_Velha_BA was best fit by a three-source model incorporating Iberia_C (~ 24%), Russia_Samara_EBA_Yamnaya (~ 18%), and Italy_C (57%) in Semiproximal_1 (*p* = 0.6439); and Iberia_C, Germany_BellBeaker, and several ancient Central Mediterranean Punic populations or ancient and present-day North African populations in Semiproximal_2. However, we found no evidence of gene flow into Torre_Velha_BA from populations related to any of these proxies (Additional file 2: Fig. S22), nor any excess African ancestry in Torre_Velha_BA compared to other Iberian regions (Additional file 2: Fig. S23). In fact, Torre_Velha_BA showed higher affinities with Bronze Age Central Mediterranean islands, as indicated by outgroup *f*_*3*_-statistics (Additional file 2: Fig. S24). Therefore, the best representative model for Torre_Velha_BA would include Iberia_C (~ 44%), Germany_BellBeaker (~ 41%), and Iran_N (~ 15%; *p* = 0.0594), with the latter serving as a deep genetic proxy for Eastern Mediterranean ancestry potentially associated with maritime interactions [13].

To further refine the ancestry profiles of sites with more complex genetic signals, we implemented a proximal framework, using Iberia_C_oSteppe—representing Chalcolithic northern Iberians already carrying Steppe-related ancestry—as a local ancestry proxy, alongside several temporally and geographically close populations to detect more recent gene flow. Torre_Velha_BA was best modeled with Iberia_C_oSteppe and Italy_C (*p* = 0.6190), a pattern also observed in Monte_da_Cabida_BA (*p* = 0.8691) and Monte_do_Gato_de_Cima_3_BA (*p* = 0.9705). In contrast, Outeiro_Alto_BA and the other sites were explained solely by Iberia_C_oSteppe (Fig. 3C; Additional file 3: Table S17).

An outlier (PT_23212) in the Torre_Velha_BA group was identified in PCA (Fig. 3A), MDS plots based on 1-* f*_*3*_(Mbuti; Ind1, Ind2) (Additional file 2: Fig. S16A), *f*_*4*_-statistics (Fig. 3C) and *f*_*3*_-statistics (Additional file 2: Fig. S24), suggesting a distinct genetic background. Despite this, no genetic ancestry from outside Iberia was significantly detected in this outlier when calculating *f*_*4*_*(*Torre_Velha_BA_o, Iberia_BA; test, Chimp/Mbuti) and *f*_*4*_*(*Torre_Velha_BA_o, Torre_Velha_C; test, Chimp/Mbuti) with *Test* including Eurasian and African populations from the Chalcolithic and Bronze Age as well as posterior time periods as proxies (Additional file 2: Fig. S25). The admixture modeling of a semiproximal framework in *qpAdm* determined the best-fitting model to include Iberia_C (~ 58%) and Iran_C (~ 42%; *p* = 0.2013; Additional file 3: Table S16).

### Iron Age and Roman Period

No new samples from the Iron Age were available for study due to the widespread practice of cremation during this period [65]. Genome-wide analysis of previously published Iron Age individuals from Spain (Spain_IA) showed that they clustered closely with Iberia_BA in the PCA (Additional file 2: Fig. S27). However, we also observed Eastern Mediterranean connections in Greek-associated sites (Additional file 2: Fig. S27) and an increase in Steppe-related ancestry (Additional file 2: Fig. S4).

**Fig. 4:**
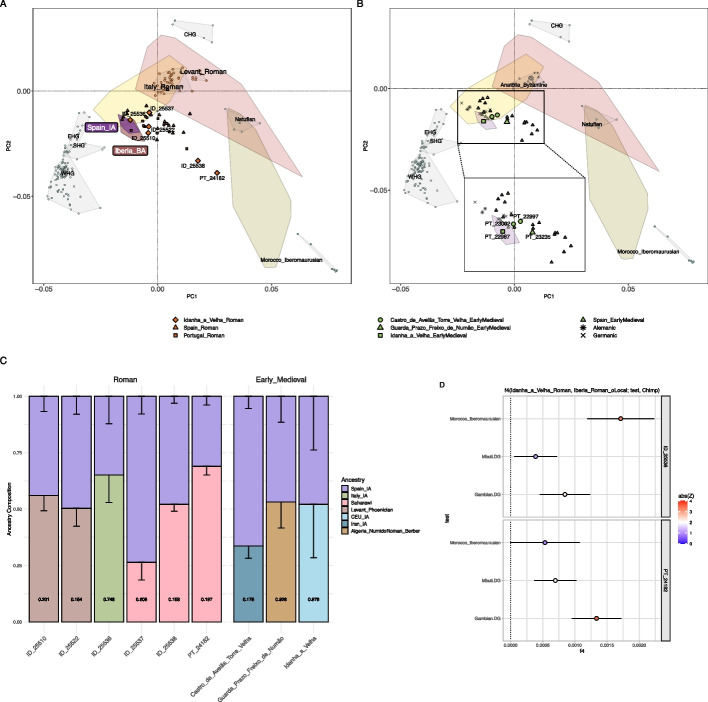
PCA of present-day West Eurasians and North Africans (overlaid colored polygons represent geographical clusters). The ancient individuals from Iberia and other regions were projected onto the first two principal components. Colors indicate different temporal periods as shown in 1B, with a focus on **A** the Roman and **B** Early Medieval periods. **C** Ancestry proportions using different admixture modeling frameworks for Idanha_a_Velha_Roman and Early Medieval archeological sites; *p*-values are provided inside each column and error bars indicate 1-SD. **D** *f*_4_-statistics of the form *f*_4_(Idanha_a_Velha_Roman, Iberia_Roman_oLocal; Test, Chimp) with *Test* including ancient and present-day African populations as proxies for the individuals clustering with present-day Africans in PCA (ID_25538 and PT_24182). The *x*-axis shows the *f*_*4*_-statistic values, with results displayed as the mean ± 1-SD

We next analyzed six unrelated individuals (genetically assigned as one male and five females) from the historical village of Idanha-a-Velha (central-eastern Portugal) (Additional file 2: Fig. S28; Additional file 3: Table S2), hereafter referred to as Idanha_a_Velha_Roman. Only a few Iberian individuals from the Roman period (Iberia_Roman) clustered with Spain_IA (Fig. 4A; Additional file 2: Fig. S29). Among these, we observed individual ID_25536, which carried the local H1e1c mtDNA haplogroup (Fig. 4A). However, *f*_*4*_-statistics of the form *f*_*4*_(ID_25536, Iberia_Roman_oLocal; Test, Chimp) revealed gene flow from Mediterranean populations. Here, *Iberian_Roman_oLocal* represents a group of previously published unadmixed Iberians from the Roman period with local ancestry, and *Test* includes Eurasian and African populations from the Roman period or their proxies (Additional file 2: Fig. S30C). This finding was further supported by *qpAdm* as the best fitting model required Spain_IA and Italy_IA as ancestry sources (Fig. 4C; Additional file 3: Table S19).

In the PCA, we also observed that most Iberia_Roman individuals do not cluster with Spain_IA, coinciding with a displacement in the PCA, where the former moved towards present-day North Africans and Southwestern Asians, as well as ancient Italy_Roman and Levant_Roman (Fig. 4A; Additional file 2: Fig. S29). In fact, a total of three female individuals from Idanha_a_Velha_Roman (ID_25510, ID_25522, and ID_25537) fell within this cluster. When performing *f*_*4*_-statistics of the form *f*_*4*_(Ind, Iberia_Roman_oLocal; Test, Chimp), we observed no external gene-flow for ID_25522 and ID_25537 (Additional file 2: Fig. S30B;D). However, we observed significant positive *f*_*4*_-statistics values for ID_25510 when African proxies were used (Additional file 2: Fig. S30A). In fact, all three of these females were best modeled with a local source (Spain_IA or Iberian_Roman_oLocal) and an additional Eastern Mediterranean or North African source (Fig. 4C; Additional file 3: Table S19), in agreement with their respective local, Eastern Mediterranean, and North African mtDNA haplogroups (K1b1a2, H1ah1, M1a3a, respectively). Interestingly, our analysis revealed no evidence for sex-biased admixture (Additional file 2: Fig. S31; Additional file 3: Table S20).

The individuals PT_24182 and ID_25538 did not cluster with Iberia_Roman in the PCA and instead shifted towards present-day North Africans (Fig. 4A; Additional file 2: Fig. S29). We observed significant positive *f*_*4*_-statistics values of the form *f*_*4*_(Ind, Iberia_Roman_oLocal; Test, Chimp) when African proxies were used (Fig. 4D; Additional file 2: Fig. S30E-F), suggesting gene-flow from Africa. These findings were further supported by *qpAdm* analysis, where all models required both an Iberian source (Spain_IA or Iberian_Roman_oLocal) and an African proxy (Saharawi or Morocco_Iberomaurusian) (Fig. 4C; Additional file 3: Table S19). Notably, we found no evidence of sex bias for Iberian or African ancestries (Additional file 2: Fig. S31; Additional file 3: Table S20). Furthermore, both individuals carried mtDNA haplogroups that are most frequently found in present-day North Africans (X1c and T1a6; Additional file 3: Table S5).

### Early Medieval Period

We sequenced a total of four unrelated ancient individuals (genetically identified as one male and three females) from the Early Medieval Period (Additional file 3: Table S2; Additional file 2: Fig. S32), including Idanha-a-Velha (Idanha_a_Velha_EarlyMedieval; *n* = 1), the necropolis of Prazo in Freixo de Numão, Guarda (Guarda_Prazo_Freixo_de_Numão_EarlyMedieval; *n* = 1), and the necropolis of Torre Velha in Castro de Avelãs (Castro_de_Avelãs_Torre_Velha_EarlyMedieval; *n* = 2). This period was marked by the collapse of the Roman Empire and the historically recorded migrations of Visigoth and Suebi peoples from Central Europe.

In the PCA, the Early Medieval Portuguese individuals from this study clustered with contemporary Spanish populations but exhibited less diversity, positioning them closer to Spain_IA. In contrast, most Early Medieval Spanish individuals showed a lesser shift towards present-day Southwestern Asians compared to Iberian_Roman. Rather, they displayed high divergence on PC1, positioning them between present-day North Africans and clustering with present-day and ancient Central Europeans (Fig. 4B).

Idanha_a_Velha_EarlyMedieval (PT_22987) showed a clear genetic affinity with CEU_IA and German_Saxon populations as computed with *f*-statistics of the form *f*_*3*_(Idanha_a_Velha_EarlyMedieval, Test; Chimp) and *f*_*4*_(Idanha_a_Velha_EarlyMedieval, X; Test, Mbuti); where *Test* includes Eurasian and African populations from the Early Medieval period or close proxies, and *X* represents different Iberian and Central European Iron Age, Roman, and Early Medieval populations (Additional file 2: Figs. S33A, S34). While this individual carried the local mtDNA haplogroup T2b3 (Additional file 3: Table S5) and their ancestry could be solely explained by Spain_IA or Iberia_Roman_oLocal in both distal and semiproximal frameworks in *qpAdm* (*p* = 0.6075 and *p* = 0.1013, respectively), the best-fitting models were obtained by including CEU_IA in the distal framework (*p* = 0.9756) and Alemanic in the semiproximal framework (*p* = 0.5885; Fig. 4C; Additional file 3: Table S21).

Even though *f*-statistics for Guarda_Prazo_Freixo_de_Numão_EarlyMedieval (PT_23235) indicated no significant gene flow from other populations (Additional file 2: Figs. S33B, S35), this individual harbored a W5 mtDNA haplogroup that is mostly found in present-day Germany, the Benelux, the British Isles, Norway, and Poland (Additional file 3: Table S5). Moreover, we were able to model PT_23235 with Spain_IA and a North African proxy (Algeria_NumidoRoman_Berber) in a distal framework (*p* = 0.9277). In contrast, in a semiproximal framework, the model fit with Iberia_Roman_oLocal improved with Algeria_NumidoRoman_Berber (*p* = 0.9467; Fig. 4C; Additional file 3: Table S21), although this ancestry source was not required (*p* = 0.4445).

As for the two individuals (one male—PT_23002; one female—PT_22997) from Castro_de_Avelãs_Torre_Velha_EarlyMedieval, both revealed possible gene flow from Eastern Mediterranean populations when computing *f*-statistics (Additional file 2: Figs. S33C, S36). This result is further supported by *qpAdm* analysis, as the best fitting model in a distal framework included Spain_IA and Iran_IA (*p* = 0.1778; Fig. 4C; Additional file 3: Table S21). In contrast, all semiproximal models failed (Additional file 3: Table S21). Interestingly, PT_23002 carried an extremely rare mtDNA haplogroup (H44b) that had not been previously reported in ancient populations and only found in present-day people from the Western Black Sea (Additional file 3: Tables S5-6) [66].

### Islamic and Christian Conquest periods

The Late Medieval period was marked by political and cultural divisions in Iberia, with the south remaining under Islamic rule for longer, while Christian conquest gradually advanced from the north. In this context, we analyzed a total of nine individuals (five genetic males and four genetic females; Additional file 3: Table S2) from an Islamic necropolis in Santarém (central Portugal; *n* = 3); and two Islamic archeological sites in Loulé (southern Portugal), namely Quinta da Boavista (*n* = 4) and Hospital da Misericórdia (*n* = 2). These sites are hereafter referred to as Santarém_Islamic, Quinta_da_Boavista_Islamic, and Hospital_da_Misericórdia_Islamic, respectively. We also analyzed four individuals from Christian burial contexts (two genetic males and two genetic females; Additional file 3: Table S2), including remains from the necropolis of Torre Velha in Castro de Avelãs (northeastern Portugal; *n* = 3) and the necropolis of Prazo in Freixo de Numão, Guarda (central Portugal; *n* = 1). Similarly, these sites are hereafter referred to as Castro_de_Avelãs_Torre_Velha_Conquest and Guarda_Prazo_Freixo_de_Numão_Conquest, respectively. We note that one individual from Castro_de_Avelãs_Torre_Velha_Conquest (PT_22994) was previously reported to carry an extra copy of the X chromosome, consistent with Klinefelter’s Syndrome (XXY; Additional file 3: Table S2) [67].

**Fig. 5:**
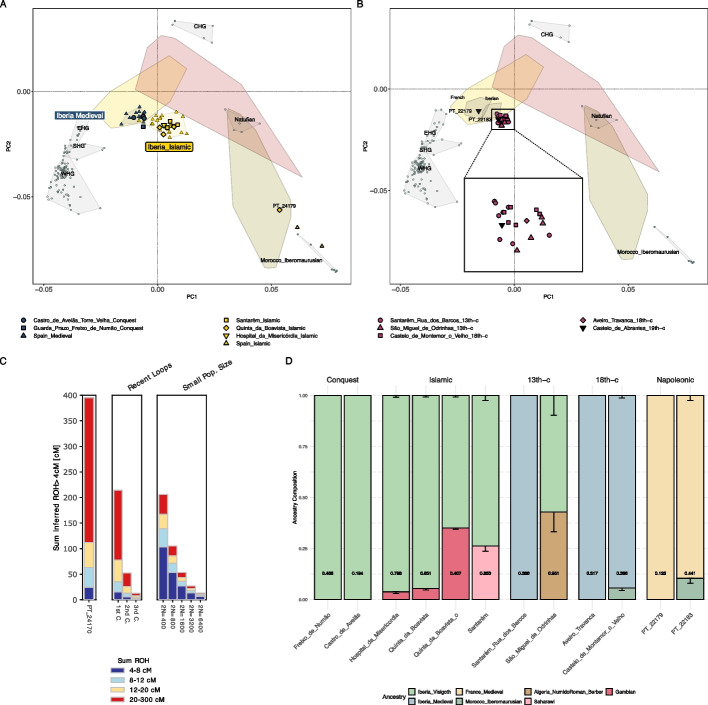
PCA of present-day West Eurasians and North Africans (overlaid colored polygons represent geographical clusters) with ancient individuals from Iberia and other regions projected onto the first two principal components. Colors indicate different temporal periods as shown in 1B, with a focus on the **A** Late Medieval period with Islamic and Christian associated sites, and **B** thirteenth to nineteenth century Portugal. **C** ROH analysis for PT_24170 from Santarém_Islamic, exhibiting cumulative ROH lengths exceeding 4 cM. **D** Ancestry proportions for Portuguese individuals from the Islamic and Christian Late Medieval period, thirteenth century, eighteenth century, and nineteenth century, *p*-values are provided inside each column and error bars indicate 1-SD

While none of the individuals were genetically related (Additional file 2: Figs. S32, S37), one individual from Santarém_Islamic (PT_24170) showed a ROH profile compatible with high levels of consanguinity. Specifically, it is very likely that PT_24170 is the offspring of a sibling pair (Fig. 5C; Additional file 2: Fig. S38), which represents the first documented case in ancient Iberia.

We observed that ancient Islamic individuals, including samples from this and previously published studies, formed a distinct cluster in the PCA, shifting towards present-day North Africans when compared to contemporary Iberians from Christian burial contexts (Fig. 5A). Notably, one outlier from Quinta_da_Boavista_Islamic (PT_24179), hereafter referred to as Quinta_da_Boavista_Islamic_o, clustered with present-day North Africans (Fig. 5A) and shifted towards present-day Sub-Saharan populations (Additional file 2: Fig. S39A).

Further supporting the differentiation between these two clusters in the PCA, we calculated outgroup *f*_*3*_-statistics of the form *f*_*3*_ (Iberia_Islamic/Iberia_Medieval, Test; Chimp), where *Test* represents several African proxies. Iberia_Islamic exhibited higher shared genetic drift with these proxies than Iberia_Medieval (Additional file 2: Fig. S40A). Moreover, we computed *f*_*4*_-ratios of the form *f*_*4*_(Han, Chimp; Iberia_Islamic/Iberia_Medieval, Y)/*f*_*4*_(Han, Chimp; test, Y), where *Y* includes different ancient Iberian populations (Iberia_N, Spain_IA, and Iberia_Visigoth), and observed significant gene flow from each African proxy into Iberia_Islamic, with no significant gene flow into Iberia_Medieval in any scenario (Additional file 2: Fig. S40B-D).

To determine whether each Portuguese Islamic site experienced gene flow from Africa, we computed *f*_*4*_-statistics of the form *f*_*4*_(Test, Spain_IA; X, Chimp), where *Test* represents each site and *X* includes Eurasian and African populations from the Islamic period or close proxies (Additional file 2: Fig. S41). Significant positive *f*_*4*_-values were generally observed across the sites when using African proxies (e.g., Gambian, Mbuti or Morocco_Iberomaurusian). Additionally, outgroup *f*_*3*_-statistics of the form *f*_*3*_(Test, Mbuti; Chimp) showed that Santarém_Islamic exhibited the highest shared drift with Mbuti, followed by Quinta_da_Boavista_Islamic_o, Hospital_da_Misericórdia_Islamic, and finally, Quinta_da_Boavista_Islamic (Additional file 2: Fig. S39B). While Santarém (ninth century) is located farther north than the two sites in Loulé (twelfth–thirteenth centuries), the site has a comparatively older date that is compatible with an early Islamic settlement (Additional file 1).

Admixture modeling using a distal framework with Spain_IA and an African proxy showed robust model support for all sites except for the outlier Quinta_da_Boavista_Islamic_o. However, the inclusion of an additional Eastern Mediterranean component improved all models and was required to explain the genetic ancestry of the outlier (Additional file 3: Table S22). Interestingly, using a semiproximal framework with Iberia_Visigoth—representing a local population containing previous Eastern Mediterranean and African ancestries present in the Peninsula—Hospital_da_Misericórdia_Islamic, Quinta_da_Boavista_Islamic, and the outlier individual required an additional Sub-Saharan African ancestry represented by present-day Gambians (Fig. 5D; Additional file 3: Table S23). While the first two sites exhibit low levels of Sub-Saharan African ancestry, the outlier individual shows ~ 40%. Conversely, Santarém_Islamic displayed significant North African ancestry (~ 26%), using Saharawi as a proxy. These results are further supported by the presence of African mtDNA and Y-chromosome haplogroups (Additional file 3: Tables S5-6). While the uniparental markers for these individuals were previously reported [68] (AF Maurer, personal communication, 2024), our newly sequenced data for Santarém_Islamic provides enhanced haplogroup resolution.

To investigate patterns of gene flow in Portuguese individuals associated with Christian burial contexts and contemporary to the previously described Islamic burial sites, we computed *f*_*4*_-statistics of the form *f*_*4*_(X, Y; Test, Mbuti), where *X* represents Guarda_Prazo_Freixo_de_Numão_Conquest or Castro_de_Avelãs_Torre_Velha_Conquest, *Y* represents various Iberian groups, and *Test* includes different Eurasian and African populations from the Medieval period or their proxies (Additional file 2: Figs. S42-43). We observed that the cladality of the *f*_*4*_-statistics was maintained, which is concordant with admixture modeling of a semiproximal framework, as both sites can be modeled as Iberian_Visigoth (*p* = *0.4562* and *p* = 0.1945, respectively; Fig. 5D; Additional file 3: Table S25). Moreover, all individuals carried local mtDNA (X2b, V, H4a1a, and H10f, the latter two being quite rare; Additional file 3: Table S5) and Y-chromosome haplogroups (Additional file 3: Table S6), indicating a degree of genetic continuity from Early Medieval populations. Nevertheless, we observed a marginally significant *f*_*4*_-value when Spain_IA was used as *Y* and Gambian as *Test* in Guarda_Prazo_Freixo_de_Numão_Conquest (Additional file 2: Fig. S43A), which was further supported by admixture modeling of a distal framework including Spain_IA and an African proxy—contributing up to ~ 25% (*p* = 0.5424; Additional file 3: Table S24). In this framework, Castro_de_Avelãs_Torre_Velha_Conquest was best modeled with Spain_IA, Western_Asia_IA and Mozabite (*p* = 0.1184). These results suggest a pattern of ancestry proportions similar to Roman and Early Medieval populations, indicating a possible genetic continuity.

To test the genetic distances between the Early Medieval and post-Christian Conquest individuals—as some were from the same archeological sites—we computed an MDS plot based on 1-* f*_*3*_(Mbuti; Ind1, Ind2) (Additional file 2: Fig. S44A). We observed that all but one sample from Castro_de_Avelãs_Torre_Velha_Conquest (PT_23237) clustered closely together, indicating no major genetic differences between the two periods. Moreover, while Idanha_a_Velha_EarlyMedieval aligned closely with individuals from Castro_de_Avelãs_Torre_Velha across both time periods, we detected considerable genetic differences between the two individuals from different periods at Guarda_Prazo_Freixo_de_Numão. Similar patterns were observed when including previously published individuals from the same time periods from Spain in an MDS plot based on 1 - *f*_3_(Mbuti; Ind1, Ind2) (Additional file 2: Fig. S44B). However, some Spanish individuals, particularly form the south, exhibited greater genetic differences, likely due to the persistence of African and Mediterranean diversity from Roman times as well as the longer occupation of Islamic forces in southern Spain.

### Kingdom of Portugal

We analyzed a total of nineteen ancient individuals dating from after the establishment of the Kingdom of Portugal in the twelfth century, including thirteenth century individuals from the archeological sites of Rua dos Barcos, in Santarém (central Portugal; six genetic males and three genetic females) and the Christian necropolis of São Miguel de Odrinhas (western Portugal; two genetic males and two genetic females); as well as eighteenth century individuals from Castelo de Montemor-o-Velho (central Portugal; four genetic females and two genetic males) and the church of Travanca in Santa Maria da Feira, Aveiro (northwestern Portugal; one genetic male) (Additional file 3: Table S2). These sites are hereafter referred to as Santarém_Rua_dos_Barcos_13th-c, São_Miguel_de_Odrinhas_13th-c, Castelo_de_Montemor_o_Velho_18th-c and Aveiro_Travanca_18th-c, respectively.

Kinship analysis (Additional file 2: Figs. S45-47) at São_Miguel_de_Odrinhas_13th-c revealed complex familial relationships among the analyzed individuals. Specifically, one female (PT_24164) and one male (PT_24163) carrying the H1 mtDNA haplogroup (Additional file 3: Table S5) shared a first-degree relationship, being identified as mother and son (Additional file 3: Table S10). The male individual also shared a third-degree relationship with another male (PT_24165) carrying a different mtDNA (T2b3) but the same Y-chromosome (J2b1b1) haplogroups (Additional file 3: Tables S5-6). PT_24165, however, is not related to PT_24164, suggesting PT_24163 and PT_24165 could be paternal cousins, great-uncle and great-nephew, or paternal half-uncle and nephew.

We found that Portuguese populations from the thirteenth to the eighteenth centuries maintained genetic continuity with local inhabitants of the Medieval period, as shown in the PCA (Fig. 5B).

Admixture modeling of sites dated to the thirteenth century—Santarém_Rua_dos_Barcos_13th-c and São_Miguel_de_Odrinhas_13th-c—required Spain_IA and Algeria_NumidoRoman_Berber as ancestry sources in a distal framework (*p* = 0.0781 and *p* = 0.6364, respectively; Additional file 3: Table S26). However, we also found good model fits for both sites when including Spain_IA, Western_Asia_IA and Saharawi (*p* = 0.4668 and *p* = 0.4138, respectively). Alternatively, using a semiproximal framework (Additional file 3: Table S27) with Iberia_Visigoth or Iberia_Medieval—representing more temporally closely local populations containing previous Eastern Mediterranean, African, and Central European ancestries present in the Peninsula—Santarém_Rua_dos_Barcos_13th-c did not require any additional source (*p* = 0.2336 and *p* = 0.3883, respectively), whereas São_Miguel_de_Odrinhas_13th-c required an additional African proxy, such as Algeria_NumidoRoman_Berber (*p* = 0.9508 and *p* = 0.9218, respectively). These results could indicate a persistence of African ancestry over time or the integration of Morisco populations into the genetic pool of the broader Portuguese population.

In the eighteenth century, admixture modeling with a distal framework for Castelo_de_Montemor_o_Velho_18th-c required Spain_IA and Algeria_NumidoRoman_Berber as ancestry sources (*p* = 0.7092; Additional file 3: Table S28), but the best model fit included Spain_IA, Iran_IA, and Mozabite (*p* = 0.7275). Notably, Aveiro_Travanca_18th-c could only be modeled using a semiproximal framework with Iberia_Medieval or Portugal_13^th^-c (Additional file 3: Table S29) (*p* = 0.3171 and *p* = 0.6436, respectively), while Castelo_de_Montemor_o_Velho_18th-c was best modeled with Iberia_Medieval and an additional African proxy in this framework. In fact, we could model both sites solely using present-day Spanish populations (*p* = 0.2975 and *p* = 0.1601, respectively).

The persistence of African ancestry in post-Islamic periods was further supported by the analysis of uniparental markers showing the presence of mtDNA (U6d3a and U6a3b) and Y-chromosome (E1b1b1b1a1 and E1b1b1a1b1a10a1a3) haplogroups commonly found in North Africa but present at low frequencies in present-day Iberians (Additional file 3: Table S5-6).

### Napoleonic period

Finally, we analyzed two genetic male individuals (Additional file 3: Table S2) buried at Castelo de Abrantes in Santarém (central Portugal), dating from the occupation of Napoleon’s forces in Portugal, hereafter referred to as Castelo_de_Abrantes_19th-c.

Interestingly, we found one individual (PT_22179) clustering with present-day French in the PCA (Fig. 5B), which was supported by the cladality of *f*_*4*_(PT_22179, France_Medieval; test, Mbuti), where *Test* included present-day Spanish and Iberians, as well as other present-day and ancient Eurasians and Africans (Additional file 2: Fig. S48A). Moreover, ancestry modeling with *qpAdm* showed PT_22179 could be explained using French_Medieval as a single source (Fig. 5D; Additional file 3: Table S30).

In contrast, the PCA for the other individual (PT_22183) showed clustering with ancient Portuguese from the thirteenth and eighteenth centuries but was also indicative of a possible admixture between French and African ancestries. This was supported by *f*_*4*_-statistics (Additional file 2: Fig. S48B) and ancestry modeling with French_Medieval and African proxies as sources (Fig. 5D; Additional file 3: Table S30). Moreover, PT_22183 carried a mtDNA haplogroup of West/Central African origin (L2b3a, Additional file 3: Table S5) that is also commonly found in Europe due to historical migrations, and a Y-chromosome haplogroup (I2a1b1a1b1a1a1a) commonly found in Eastern and Southeastern Europe (Additional file 3: Table S6).

## Discussion

The genetic profile of Cova_das_Lapas_N is in agreement with previous findings in the Iberian Peninsula, which also found admixture between local hunter-gatherer populations and migratory groups related to Anatolian farmers [13, 15, 27, 36, 54]. Furthermore, we observed that Neolithic Portugal retained higher proportions of hunter-gatherer ancestry compared to other parts of Iberia, suggesting substantial genetic contributions of these groups despite the cultural transition into agricultural practices.

We also detected a persistence of mtDNA haplogroups from Upper Paleolithic and Mesolithic Iberia linked to LGM refugia and postglacial re-expansions, as well as haplogroups associated with Early Neolithic migrations from Southwestern Asia [69, 70]. Specifically, we were only able to identify I2a Y-chromosome sub-haplogroups among the male individuals from Cova_das_Lapas_N. This lineage has been previously connected to Central European hunter-gatherers who admixed with East Mediterranean Neolithic farmers during the westward expansion of the Cardial culture along the Mediterranean in the Neolithic [54]. These East Mediterranean Neolithic farmers carried the Y-chromosome haplogroup G2a (absent in Cova_das_Lapas_N) and mtDNA haplogroup K1a (present in Cova_das_Lapas_N). Although this admixture occurred approximately 2000 years earlier in Central Europe, the genetic evidence in Cova_das_Lapas_N suggests that this pattern of admixture persisted over time and spread geographically. The site, dating back to the Middle/Late Neolithic (*ca.* 3,300 BCE), postdates the Cardial culture period in Portugal (5500–5000 BCE), but exhibits a stronger shared genetic drift with Neolithic populations from the Mediterranean coast associated with the Cardial culture.

Furthermore, we found some evidence for patrilocality in Cova_das_Lapas_N, as previously suggested for other Neolithic archeological sites in Europe [27, 71–73]. Specifically, we identified two distinct Y-chromosome sub-haplogroups from the same clade, in contrast to high mtDNA haplogroup diversity; paternal first- and second-degree familial relationships; and a higher kinship coefficient among unrelated males compared to unrelated females and male–female pairs. We note, however, that the limited sample size and the scarcity of published genomes from Neolithic Portugal make it difficult to reliably measure Y-chromosome diversity—seemingly lower than in Spain (Additional file 3: Table S31)—and thus we remain cautious in our interpretation. Importantly, the study of grave goods and preliminary anthropological analysis in Cova_das_Lapas_N suggest a complex management of the burial site (Additional file 1; AM Silva, personal communication, 2024), reinforcing the idea that permanent interactions between Neolithic communities—rather than mere admixture—played a crucial role in shaping these Neolithic populations. These interactions involved the exchange of goods, information flow, acculturation, and probably female exogamy, consistent with the evidence for patrilocality.

We observed that the earliest presence of Steppe-related ancestry in Portugal occurred during the Chalcolithic, albeit at low levels and specifically in Bell Beaker-associated sites, as previously proposed [30]. Notably, however, males from these contexts lack the Steppe-associated Y-chromosome haplogroup R1b-DF27, raising intriguing questions about the dynamics of admixture. One possibility is that Steppe-related ancestry arrived in these communities, at least initially, through female-mediated gene flow—potentially linked to practices of female exogamy, as suggested by evidence from later Early Bronze Age Iberian groups [74]. Nevertheless, we caution that the lack of R1b-DF27 does not necessarily imply sex-biased admixture: stochastic sampling effects, genetic drift (given the lower effective population size of the Y-chromosome compared to the autosomes), or even male-mediated admixture involving individuals not carrying R1b-DF27 lineages could equally explain this pattern. Further sampling and direct radiocarbon dating of these sites and individuals may help clarify these dynamics.

Furthermore, the lower proportion of Steppe-related ancestry in Bell Beaker-associated individuals from Portugal compared to those from Central Europe could challenge the hypothesis of a Portuguese origin of the Bell Beaker culture. Nonetheless, we note that genetic ancestry alone does not indicate cultural diffusion directionality and that Steppe-related ancestry is only one of the proxies for Bell Beaker cultural transmission. In fact, our results point to a regional genetic continuity and population persistence in Chalcolithic Portugal. We identified the same uniparental markers in Cova_das_Lapas_C as in the earlier Cova_das_Lapas_N population as well as admixture proportions between hunter-gatherer and Anatolian-related farmer ancestries comparable to those observed during the Neolithic. Accordingly, the lack of a detectable resurgence of hunter-gatherer ancestry during the Chalcolithic in Portugal suggests a complete assimilation of hunter-gatherer ancestry in the Early Neolithic [25, 26, 31, 32].

This regional genetic continuity aligns with the persistence of Magdalenian-associated ancestry in Neolithic and Chalcolithic Iberia, which contrasts with its decline in northern/northeastern Iberia and other parts of continental Europe [5, 13–15, 27]. While this finding supports the region’s role as a Late Pleistocene refugium, it also underscores the role of local demographic processes, given the low variance explained by latitude alone. Hence, further sampling of Upper Paleolithic to Chalcolithic populations may shed light on finer-scale dynamics.

While Bronze Age Portugal showed strong regional ties and population continuity, as evidenced by the persistence of local mtDNA haplogroups, we identified a notable spread of Steppe-related ancestry, associated with the migration of people from the North Pontic Steppe into Central Europe, which eventually reached the Iberian Peninsula and had a broad demographic impact [13, 30, 31, 36, 74]. Nonetheless, we found a nuanced decline in Steppe-related ancestry towards the south and southwest of the Iberian Peninsula, indicating that this migration had a relatively lower impact in Portugal compared to other regions of the Peninsula. This diminishing trend in Steppe-related ancestry forms a gradient that likely reflects dilution through admixture with local Chalcolithic populations as the migrants moved southwestward from the Peninsula’s northeast [27, 36]. Moreover, despite the complete replacement of local Y-chromosome lineages by distinct R1b-DF27 Y-chromosome lineages, our admixture modeling comparisons between autosomes and the X chromosome detected no evidence of a sex bias in Steppe-related ancestry, thereby challenging the hypothesis of a male-driven Bronze Age transition in Portugal [13, 27, 37].

Interestingly, our data also revealed connections to the Mediterranean Sea during the Bronze Age, which had only been sporadically reported in southern Spain (individual ZAP002; Fig. 3A) [37]. These findings indicate the possibility of early contacts between western Iberia and Mediterranean cultures, potentially linked to maritime trade and cultural exchanges that peaked during the Iron Age, but which predate the foundation of the Phoenician city of Cádiz [41]. In fact, this genetic signal could be representing the earliest signatures of contacts with Phoenician populations, following the African coast of the Mediterranean and migrating into southwestern Iberia [41]. These contacts could have also played a role in the development of the Tartessian culture in southwestern Iberia, reflecting a broader pattern of cultural and genetic exchange across the Mediterranean and further west to the Atlantic coast that occurred as early as the Bronze Age.

Due to the scarcity of archeological remains from the Portuguese Iron Age, which is largely attributed to prevalent funerary cremation practices [65], we were unable to access new samples from this period. However, our analysis of previously published Spanish populations revealed a genetic continuity through the Iron Age, with notable connections extending to the Eastern Mediterranean and an increase in Steppe-related ancestry, a pattern that was previously reported [13].

The genetic analysis of individuals from the Roman period sampled at the historical village of Idanha-a-Velha revealed significant diversity, supporting the hypothesis that this ancient city functioned as a key crossroad within Lusitania, linking the capital cities Emerita Augusta, Bracara Augusta, and Lucus Augusti [75]. The presence of distinct African and Mediterranean ancestries highlights the impact of extensive trade, migratory networks, and cultural exchanges characteristic of Roman Iberia [76, 77]. In this period, migrant Roman citizens that were part of the social elite were buried in mausoleums of Italic tradition, as shown by architectural elements found in the area [78]. Conversely, the presence of enslaved individuals and freedmen, some with Greek names and potentially of Eastern Mediterranean or North African origin, reflects a more complex social dynamic with varying levels of integration [78], as also indicated by epigraphic evidence of military personnel involved in public works and gold mining [78]. Hence, the existence of African ancestry in Idanha-a-Velha may be linked to trade, as supported by the discovery of amphorae and fine tableware from Roman Africa, reflecting the role of the city in broader Mediterranean trade networks.

Recent paleogenomic studies have documented a genetic shift towards Anatolian and Levantine ancestries in the Imperial Roman period compared to earlier Iron Age populations in Italy, the Southern Arc and the Balkans [34, 45, 79, 80]. We found a similar ancestry shift among some individuals from Idanha_a_Velha_Roman, while others exhibit a notable shift towards North African ancestry. While we were not able to include Iron Age samples in our study, these patterns are similar, albeit less pronounced, to previous observations in Italy [80], possibly reflecting a higher degree of genetic continuity from Iron Age populations within the Roman period in Portugal compared to Italy.

In the aftermath of the fall of the Western Roman Empire, historical records document the arrival of Germanic tribes from Eastern and Central Europe, in particular Suebi and Visigoth peoples, to Gallaecia and Lusitania, two Roman provinces overlapping with contemporary Portuguese territory [46, 47]. Accordingly, our analysis in Idanha-a-Velha identified an Early Medieval female individual with significant Central European ancestry, likely associated with the arrival of Visigoths.

In contrast, we found one individual with a predominant Iberian ancestry and a minor North African component in Guarda_Prazo_Freixo_de_Numão_EarlyMedieval, as well as two individuals from Castro_de_Avelãs_Torre_Velha_EarlyMedieval showing a mixture of local and Eastern Mediterranean ancestry. Our results align with recent evidence of North African ancestry persistence in the Visigothic period in eastern Iberia [77]. These genetic influences were likely rooted in population connections established during the Roman period. This finding highlights a more intricate pattern of genetic ancestry and population dynamics in the Iberian Peninsula during the Early Medieval period, underscoring the need for further sampling to fully resolve these complexities.

The genetic analysis of individuals from Islamic and Christian sites sampled across Portugal during the Late Medieval period revealed a more diverse genetic background in the former case, characterized by substantial Sub-Saharan and North African ancestry. This is particularly evident in Islamic contexts located in southern Portugal, probably due to the more prolonged period of Islamic presence in southern regions.

While cousin marriages were common in the Islamic period to preserve family wealth and strengthen alliances [81], the ROH profile of one individual sampled in Santarém_Islamic indicates that this individual was likely the offspring of a sibling pair—which is the first example identified in ancient Iberia. Although Islamic law explicitly prohibits sibling, parent-offspring, and certain other close relative unions (Quran 4:23), this finding may reflect rare deviations from religious norms, possibly influenced by local practices or constrained social and geographic marriage pools, which could have been a consequence of an early Islamic settlement—as recently shown [77]—further corroborated by higher proportions of African ancestry.

In contrast, the genetic diversity found in contemporary Christian contexts suggested a strong preservation of local genetic ancestry lasting from the Early Medieval period, with minimal additional external gene flow, as recently suggested [82]. Specifically, genetic analysis of individuals from Castro_de_Avelãs_Torre_Velha_Conquest revealed very limited evidence for Islamic influence in northern Portugal after the brief expansion of Islamic groups into the region. We note that Castro de Avelãs is strategically located near the Douro River, a crucial geographical barrier previously described as the *Desierto del Duero* (“*Duero Desert*”) by Sánchez-Albornoz. This region was sparsely populated and witnessed significant military activity, including a brief period of Islamic control during the campaigns of Al-Mansur, the *de facto *ruler of the late Umayyad Caliphate of Córdoba [52].

Portuguese populations from the thirteenth to eighteenth centuries exhibit a genetic continuity from the post-Islamic period, highlighting the persistence of local genetic signatures over several centuries. However, admixture modeling detected a small yet notable proportion of African ancestry, further supported by uniparental markers, documenting the lasting genetic impact of the earlier Islamic presence in the region. This suggests that the genetic influence of Islamic populations persisted long after their political power declined, potentially due to the integration of Morisco populations into the broader Portuguese population, the frequent mobility between Europe and North Africa during the Medieval and Modern periods [52], and/or subsequent waves of African migrants.

The genetic analysis of two soldiers from the Napoleonic army sampled at Castelo_de_Abrantes_19th-c provided insights into the origins of some of the French troops that occupied Portugal. The first individual exhibited a clear genetic affinity with Medieval and present-day French populations, which is consistent with the historical context, as Napoleon’s occupation force was primarily composed by soldiers from France [83]. The second individual presented a more diverse genetic background with both Medieval French and African ancestries. This individual carried a mtDNA haplogroup L2b3a, which is of African origin, being particularly prevalent in West and Central Africa. Interestingly, this observation suggests Napoleon’s ranks included soldiers of African descent, either through direct recruitment or as descendants of earlier African immigrants to France. In fact, Napoleon’s armies were known for their diverse composition, often incorporating soldiers from various parts of Europe, including those from territories under French influence or control [83], highlighting the extensive reach of Napoleon’s military campaigns.

## Conclusions

In this study, we reconstructed the genetic history of Portugal from the Neolithic to the contemporary period, revealing a complex and dynamic population history.

We identified early admixture between local hunter-gatherers and Anatolian-related farmers in Neolithic Portugal, with a northeastern–southwestern gradient of increasing Magdalenian-associated ancestry persistence. This genetic signature extended through the Chalcolithic, when the earliest evidence of Steppe-related ancestry could be detected in Portugal, significantly increasing in the Bronze Age. Our data revealed contacts between western Iberia and the Mediterranean Sea during the Bronze Age, which represents genetic evidence associated with cultural exchanges and maritime trade predating the arrival of Phoenicians in the Iberian Peninsula.

The genetic analysis of the Roman period highlighted the important role of migration, and the strengthening of trade networks as evidenced by genetically diverse populations, including ancestry from Europe, Africa, and the Mediterranean. This trend further enriched in the Early Medieval period, with the arrival of Suebi and Visigoth migrants from Central Europe.

During the Islamic and Christian Conquest period, northern Portugal exhibited strong genetic continuity from the Early Medieval period, while the south of the country revealed significant admixture with African populations. These observations align with historical records of greater cultural and political influences of Islam in the south of the Iberian Peninsula compared to northern regions. The enduring presence of African ancestry into the post-Medieval period also reflects the lasting impact of these cultures on the genetic make-up of the contemporary Portuguese population.

Overall, our study highlights the intricate genetic history of Portugal, shaped by centuries of migration, cultural exchange and interaction, with significant contributions from diverse populations. Crucially, we also detect patterns of continuity that reflect the geographical isolation of the region, located at the westernmost edge of continental Eurasia and constituting an effective *cul-de-sac* that deserves further study in the future.

## Methods

### Experimental design

#### Archeological samples

A total of 94 individuals from Portugal dated to a time span from ~ 3000 BCE to ~ 1800 CE were analyzed for the presence of ancient DNA. A detailed description of the archeological context, sites, and individuals is reported in Additional file 1 and summarized in Additional file 3: Table S1.

### Laboratory facilities

Pre-amplification experiments took place at the ultra-clean laboratory facilities of the Australian Centre for Ancient DNA (ACAD). Stringent laboratory procedures were implemented to minimize contamination and uphold the genetic data’s high-quality standards [84, 85]. All post-amplification experiments were conducted in standard molecular biology laboratories at the University of Adelaide, followed by subsequent bioinformatics workflows executed on the University of Adelaide’s High-Performance Computer.

### DNA Extraction and Library Preparation

We preferentially sampled petrous bones and teeth. Details of sampled skeletal elements are listed in Additional file 3: Table S1. Before DNA extraction, the skeletal samples underwent sterilization using UV, bleach, ethanol, and/or scraping the bone surface to minimize contamination. Approximately 0.1 g of bone powder was utilized for DNA extraction. A method optimized for degraded DNA [86] was employed to retrieve ancient DNA molecules, and subsequently, partially UDG-treated [87] double-indexed double-stranded DNA libraries were generated [88]. Quality controls and DNA quantification were carried out using Qubit (Thermo Fisher) and TapeStation (Agilent). DNA-sequencing libraries were sent for sequencing on a NovaSeq 6000 platform at the Kinghorn Centre for Clinical Genomics (Sydney, NSW, Australia).

### Data processing

Raw data underwent processing using the aDNA analysis workflow package nf-core/eager version 2.4.6 [89]. Merged read mates were aligned to the GRCh37d5 reference genome using *bwa aln* with parameters *-l 1024 -n 0.01 -o 2* [90]. Trimming of 2 nt from the terminal ends of all retained reads was performed using the *trimBam* function of *bamUtil* (https://github.com/statgen/bamUtil). Standard quality filters (mapping quality ≥ q25 and base quality ≥ Q30) were applied, and reads were deduplicated using the *MarkDuplicates* function from Picard.

### Genetic sex determination

Processed shotgun sequencing data was used to determine genetic sex. Relative depth of coverage on the X- and Y-chromosomes were calculated using SexDetERRmine (https://github.com/nf-core/modules/tree/master/modules/nf-core/sexdeterrmine) with default quality cut-off values for -q30 and -Q30 (Additional file 3: Table S2).

### Authentication and quality control

DamageProfiler [91] was used to assess aDNA authenticity, calculating fragment size distributions and post-mortem damage rates at the read termini (Additional file 3: Table S1). We also calculated endogenous DNA proportions after mapping, filtering, and deduplication (Additional file 3: Table S1). The nuclear contamination function from ANGSD [92] was used to calculate the Maximum Likelihood and Method of Moments contamination estimates according to Method 1 and 2 (Additional file 3: Table S2). Males displaying unexpected levels of heterozygosity on the X chromosome were excluded from downstream analyses. All female individuals with at least 10 SNPs showed heterozygosity consistent with expectations. For females and males with insufficient data for reliable nuclear contamination estimates—such as those with very few or zero usable SNPs—we instead relied on mtDNA-based contamination estimates, see “Mitochondrial DNA” section.

### Library enrichment

A total of 83 libraries met authenticity quality thresholds and underwent enrichment. Over-amplification was conducted to achieve the 1000 ng required for enrichment. For each library, the PCR reaction mix consisted of 5–10 µl of library, 25 µl of KAPA HiFi HotStart ReadyMix (Roche), 5 µl each of 10 µM IS5 and IS6 primers [87], and ultrapure water in a total volume of 50 µl. PCR amplification was performed with an initial denaturation and polymerase activation at 98 °C for 2 min, 15 cycles of 98 °C for 20 s, 56 °C for 30 s, 72 °C for 45 s, and a final extension at 72 °C for 5 min. DNA purification was performed using 1.2 × AmpureXP beads with two 80% ethanol washes. The DNA was eluted in 30 µl of water.

Pooling of libraries was determined from total DNA quantification of each library, considering the endogenous content, as described in [93, 94]. Enrichment was performed using the Twist Bioscience “Twist Ancient DNA” reagent, following the manufacturer’s protocol. The post-enrichment PCR amplification was performed using KAPA HiFi HotStart ReadyMix (Roche) and IS5 and IS6 primers as described above, with a 98 °C initialization for 24 s, 7 cycles of 98 °C for 15 s, 60 °C for 30 s, 72 °C for 30 s, and a 72 °C final extension for 60 s. Some libraries underwent a reconditioning PCR to reduce heteroduplexes. Specifically, libraries were concentrated down to 5 µl and mixed with 10 µl of Herculase Buffer (Agilent), 5 µl of 2.5 nM dNTPs, 1 U of Herculase II Fusion (Agilent), 1 µl each of 10 µM IS5 and IS6 primers, and ultrapure water in a final volume of 50 µl. This solution was then reconditioned with one cycle of 95 °C for 2 min, 58 °C for 2 min, and 72 °C for 5 min. DNA purification and library quality control and quantification were performed as described above. Enriched libraries were sent for sequencing on a NovaSeq 6000 platform at the Kinghorn Centre for Clinical Genomics (Sydney, NSW, Australia).

### Genotyping

Sequencing raw data underwent processing as previously described (Additional file 3: Table S3). Pseudohaploid variant calling was executed using the Twist Bioscience “Twist Ancient DNA” SNP panel [64] with *pileupCaller* (https://github.com/stschiff/sequenceTools).

As expected, the hybridization procedure ensured efficient enrichment for DNA targets, resulting in increased endogenous DNA proportions and a decrease in the complexity of the libraries (Additional file 3: Table S3). After selecting individual samples that met the following criteria: (i) more than 35,000 SNPs covered by at least one read of the Twist SNPs panel; (ii) the presence of the misincorporation patterns characteristic of aDNA (> 3%); and (iii) absence of contamination; a total of 67 samples were retained for genome-wide data analyses.

### Uniparentally inherited markers

#### Mitochondrial DNA

The analysis of mitochondrial DNA involved merging the raw data obtained from two distinct sources: (i) data generated using the Twist Bioscience “Twist Ancient DNA” reagent [64], and (ii) data obtained with the myBaits Expert Human Affinities Prime Plus Kit by DAICEL Arbor Biosciences (Ann Harbor, MI, USA). These datasets were processed following the respective manufacturers’ protocols and utilizing the pooling technique outlined in [94]. As previously validated [64, 95], no allelic biases were present in the mitochondrial capture performed with the myBaits Expert Human Affinities Prime Plus Kit.

The raw data from both sources underwent processing using the aDNA analysis workflow package nf-core/eager version 2.4.6 [89]. Merged reads were then mapped to the mitochondrial revised Cambridge Reference Sequence (rCRS) using CircularMapper (https://github.com/apeltzer/CircularMapper) and *bwa aln* with parameters *-l 1024 -n 0.01 -o 2 -k 2* [90]. Read trimming and filtering followed the procedures outlined above (Additional file 3: Table S4). The read pileups were visually inspected using Geneious v2022.1.1 (Biomatters; https://www.geneious.com) (Additional file 3: Table S4). Mitochondrial haplogroup calling (Additional file 3: Table S5) was performed using *haplocart* [96], and contrasted using mitoverse HaploCheck version 1.3.2 [97], which also enabled the estimation of contamination levels. In Additional file 3: Table S5, columns J and K refer to “Contamination Status—HaploCheck” and “Contamination Level—HaploCheck”, respectively. “ND” refers to “Not Detectable” and is a more cautious statement, suggesting no contamination within the limits of detection. “NO” is a more conclusive statement, indicating that no contamination was detected. Hence, we concluded no contamination for the analyzed samples.

### Y-chromosome

The Y-chromosome genotype was determined using the *UnifiedGenotyper* tool from Picard v2.26.0 (Broad Institute, 2019), and inferred from the merged, trimmed BAM files obtained through the nf-core/eager pipeline. The analysis was conducted using the GRCh37d5 genome as a reference. Only the Y-chromosome SNPs identified from the Twist Bioscience “Twist Ancient DNA” SNP panel [64] were considered for analysis. The python script hGrpr2.py from *HaploGrouper* (https://gitlab.com/bio_anth_decode/haploGrouper) was used to obtain the Y-chromosome haplogroups with the default tree and the SNP file from the Ghr37 reference genome (Additional file 3: Table S6).

### Kinship estimation

BREADR v1.0.2 was used to determine kinship between pairs of individuals from the same archeological site using the default settings [98]. READv2 [99] was used to confirm results and determine resolution within first-degree relationships. We selected previously published individuals to establish a baseline of unrelated individuals. These individuals were chosen based on data generated in a similar manner (captured and partially repaired) (Additional file 3: Table S7-10). Average kinship coefficients of unrelated and related pairs for Cova_das_Lapas_N were calculated based on the READv2 results (Additional file 3: Table S8).

### Population genetic analysis

#### Dataset

We merged our final dataset genotyped using enriched data with previously published datasets of ancient and modern individuals [13–15, 19, 20, 25, 30, 31, 33–37, 45, 53–59, 61, 62, 80, 100–172, 172, 173] reported by the Reich Lab [173](https://reich.hms.harvard.edu/datasets; please see Additional file 3: Table S32 for a detailed list of individuals and the new labels used).

### PCA

We computed PCA using the *smartpca* software from the EIGENSOFT package (v7.2.1) with the *lsqproject* and *SHRINKMODE* option *YES* and an extended list of modern and ancient populations from Eurasia, Africa, and the Caucasus. The ancient individuals were projected onto PC1 and PC2.

### F-statistics

If two individuals showed evidence for a kinship relation, we removed the one with the lowest number of SNPs for population genetics analyses, but not for archeological-specific analyses. F-statistics were computed with ADMIXTOOLS v5.1 (https://github.com/DReichLab). For *f*_*4*_-statistics, we used qpDstat and the activated *f4*-mode. *f*_*3*_-statistics were calculated using qp3Pop; and *f*_*4*_-Ratios were calculated using D4RatioTest.

### Admixture modeling

As described above, relatives with the lowest number of SNPs were discarded from the analysis.

ADMIXTURE was used to define the main genetic cluster profiles. Data was pruned for linkage disequilibrium using PLINK with parameters *–indep-pairwise 50 10 0.1* and *–geno 0.999*. Four populations were selected as fixed source ancestry groups (*k* = 4) implementing a supervised ADMIXTURE model with ten replicates.

To model the genetic ancestry of the newly sequenced ancient individuals, we used the *qpAdm* program from the ADMIXTOOLS v5.1 package (https://github.com/DReichLab), with the “*allsnps: YES*” option to maximize the number of SNPs used and subsequently increase the power to discriminate different models.

We also used *qpAdm* to quantify the proportion of genetic ancestry contributed by each source population. The ancestry proportions in the target population were inferred on the basis of how the target population is differentially related to a set of reference/outgroups via the source populations.

For all the models applied here, we used a set of 12 outgroups in total (Mbuti.DG, Ethiopia_4500BP.DG, Papuan.DG, Belgium_UP_GoyetQ116_1, Czech_Vestonice, Italy_North_Villabruna_HG, Russia_MA1_HG.SG, Russia_Ust_Ishim_HG.DG, Malalmuerzo).

Best model fits, unless otherwise indicated, were defined by the lowest number of sources, the largest *p*-value, and ancestry proportions higher than twice their standard errors [174].

### Sex bias

To assess potential sex bias, we ran separate *qpAdm* models for the autosomes and the X chromosome using the parameters allsnps: YES and chrom: X, respectively. Statistical significance was assessed using Z-scores, following previous studies [37, 111, 113] (Additional file 3: Table S18; Additional file 3: Table S20).

### Runs of homozygosity

We used hapROH VERSION 0.64 with default settings to identify runs of homozygosity within the genome [175]. Only individuals with > 400,000 SNPs covered in the panel were used in this analysis (Additional file 2: Fig. S38).

## Supplementary Information

Additional file 1: Archaeological information

Additional file 2: Supplementary figures

Additional file 3: Supplementary tables

## Data Availability

The raw sequencing data and the processed aligned sequences generated for this study are available through the Sequence Read Archive (SRA), under BioProject PRJNA1248585 [176]. The corresponding genotype dataset is available on Figshare: https://doi.org/10.6084/m9.figshare.29438474.v2 [177].
