# Supplementary material for: The genetic history of Portugal over the past 5,000 years
Source: Genome Biol. 2025 Aug 18;26:248. doi: 10.1186/s13059-025-03707-2 (PMC12360031; doi:10.1186/s13059-025-03707-2)
Supplement: Supplementary file 2 — Additional file 2: Supplementary figures [file 13059_2025_3707_MOESM2_ESM.pdf]

Additional file 2: Supplementary figures

The genetic history of Portugal over the past 5,000 years

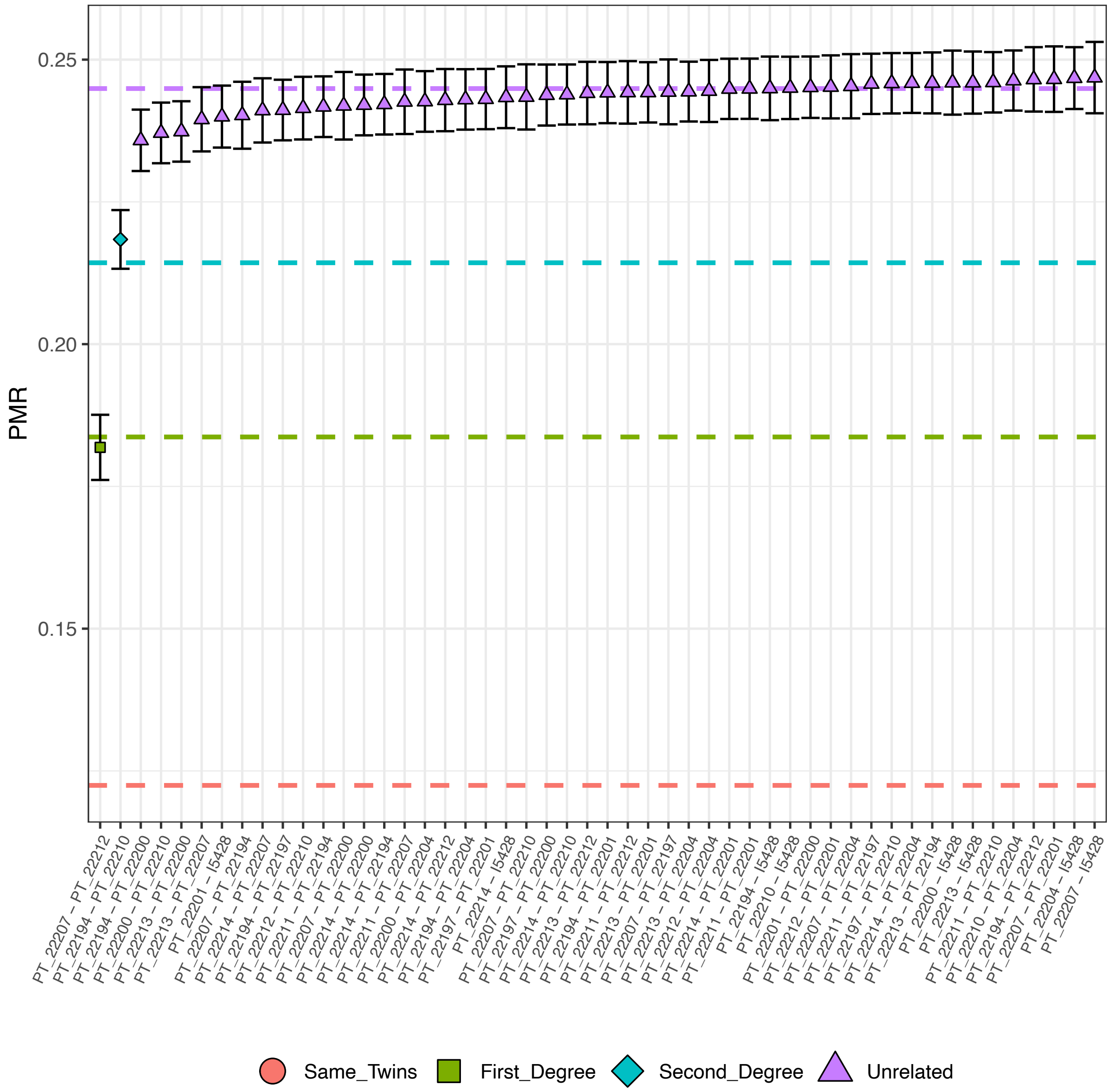

Fig. S1. Kinship analysis from Cova_das_Lapas_N/C using BREADR.

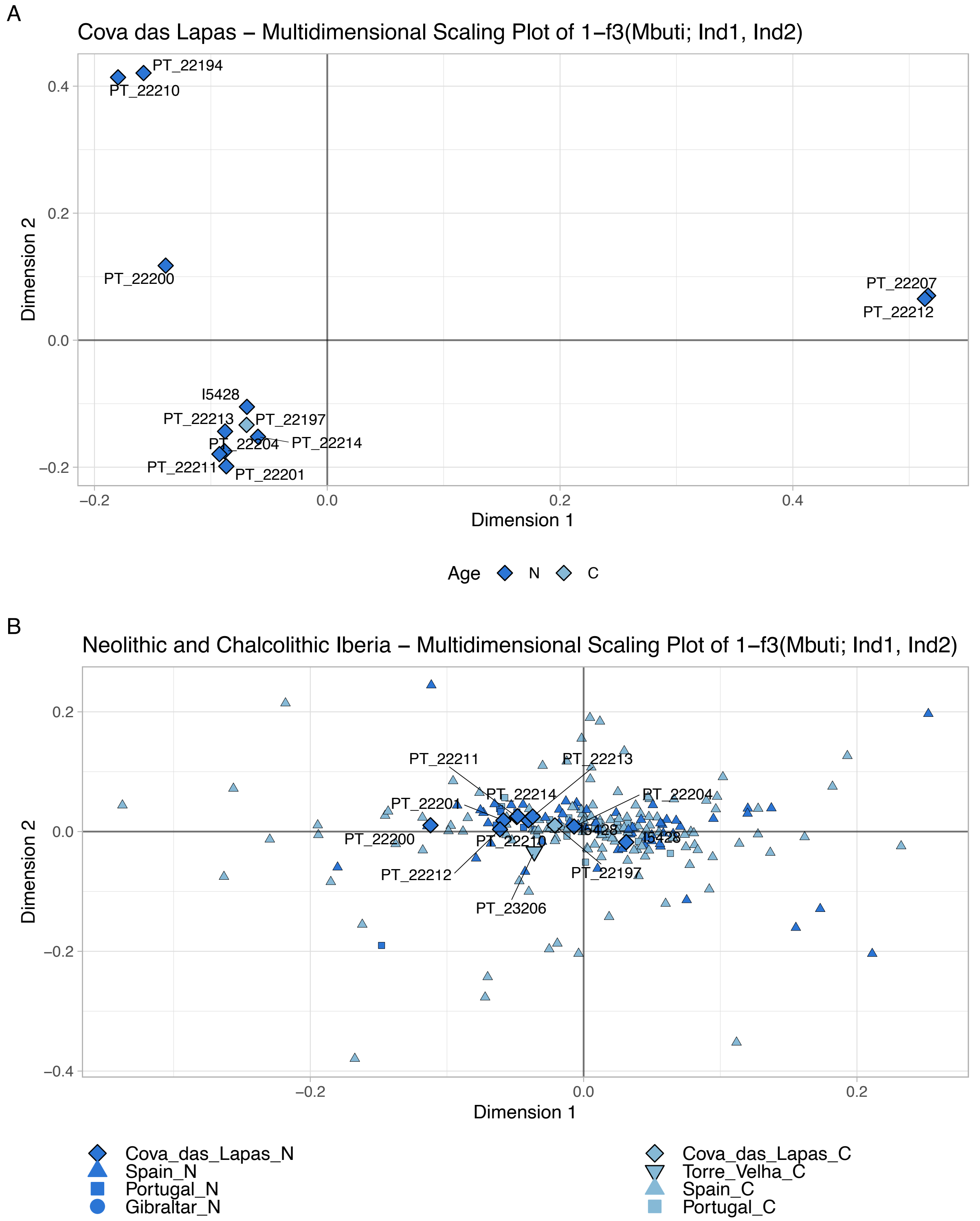

Fig. S2. Multidimensional scaling plot based on 1-f_3(Mbuti; Ind1, Ind2) for the Neolithic and Chalcolithic individuals from (A) Cova das Lapas and (B) Iberia.

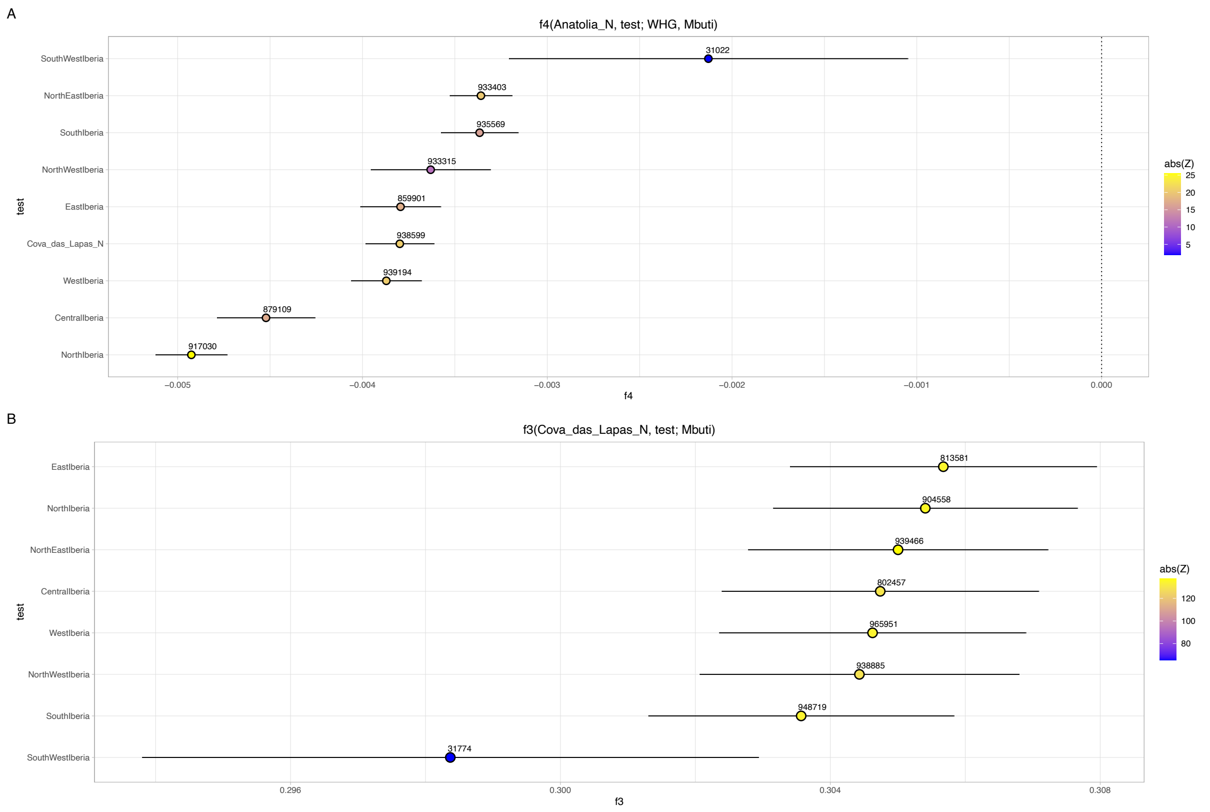

Fig. S3: The x-axes represent the f_3- or f_4-statistic values, with results displayed as the mean ± 1-SD, and colors representing Z-scores. The numbers above each dot indicate the number of SNPs used for each calculation. (A) f_4-statistics in the form f_4(Anatolia_N, Test; WHG, Mbuti) with *Test* including Cova_das_Lapas_N and different Iberian Neolithic geographical areas. (B) Outgroup f_3-statistics of the form f_3(Cova_das_Lapas, Test; Mbuti) where *Test* includes various Iberian Neolithic geographical areas.

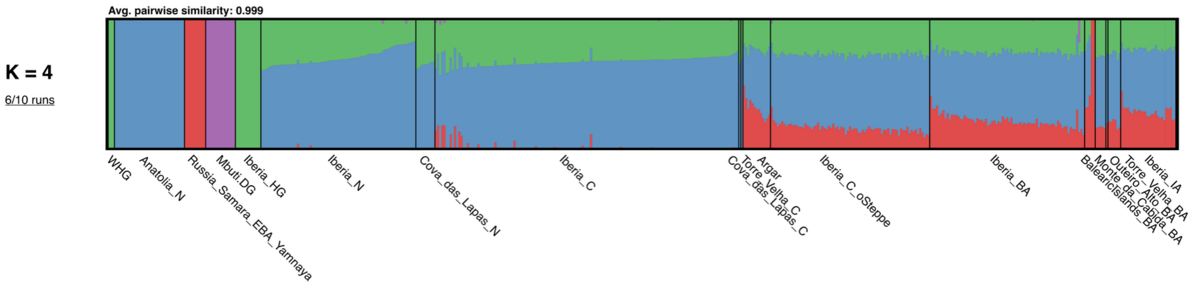

Fig. S4. Ancestry proportions estimated using supervised ADMIXTURE (k=4), with the four predefined ancestries being Western Hunter-Gatherers (WHG; green), Neolithic Southwestern Asian farmers (Anatolia_N; blue), Bronze Age Western Steppe herders (Yamnaya_BA; red) and present-day Sub-Saharan Africans (Mbuti; purple). Ancient Iberians whose ancestry proportions were estimated, include individuals from the pre-Neolithic (HG), Neolithic (N), Copper Age (C), Bronze Age (BA) and Iron Age (IA), as well as the individuals from Cova_das_Lapas_N/C, Torre_Velha_C/BA, Outeiro_Alto_BA, and Monte_da_Cabida_BA.

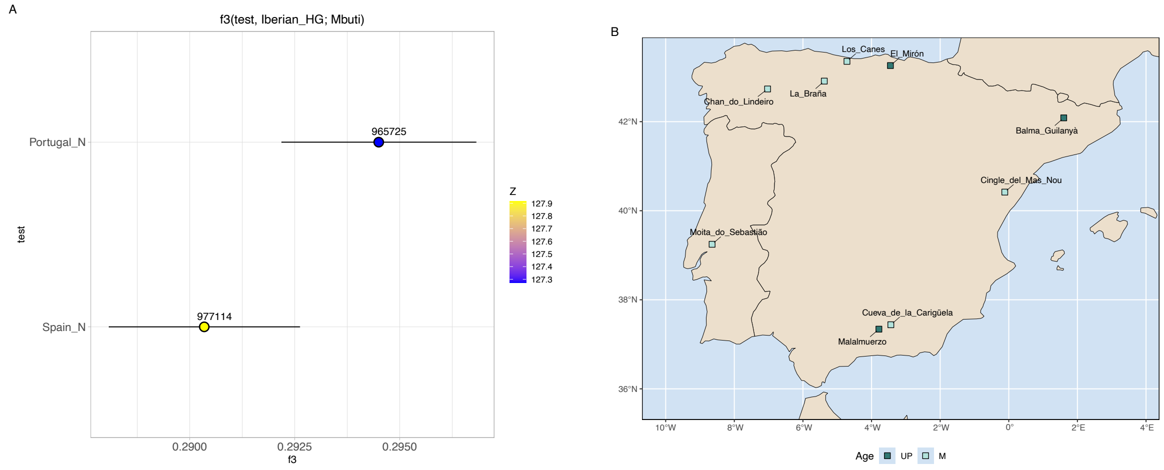

Fig. S5: (A) The x-axes represent f_3-statistic values, with results displayed as the mean ± 1-SD, and colors representing Z-scores. The numbers above each dot indicate the number of SNPs used for each calculation. f_3-statistics of the form f_3(test, Iberian_HG; Mbuti) where *Test* includes Portugal_N and Spain_N. (B) Geographical location of Iberian_HG individuals in the peninsula.

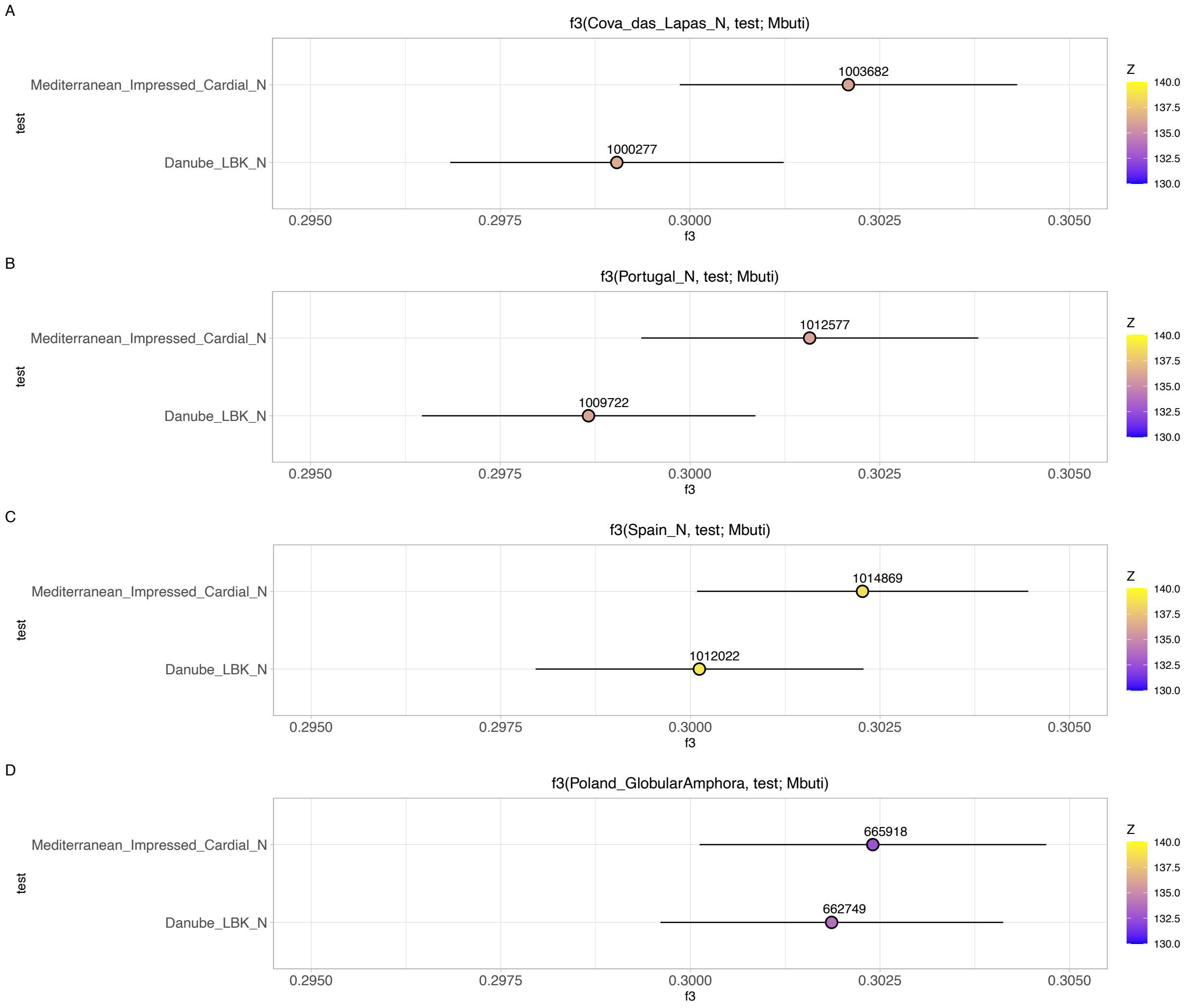

Fig. S6: The x-axes represent f_3-statistic values, with results displayed as the mean ± 1-SD, and colors representing Z-scores. The numbers above each dot indicate the number of SNPs used for each calculation. Outgroup f_3-statistics of the form f_3(X, Test; Mbuti) where *Test* includes Mediterranean_Impressed_Cardial_N or Danube_LBK_N and *X* inlcude (A) Cova_das_Lapas_N, (B) Portugal_N, (C) Spain_N, and (D) Poland_GlobularAmphora.

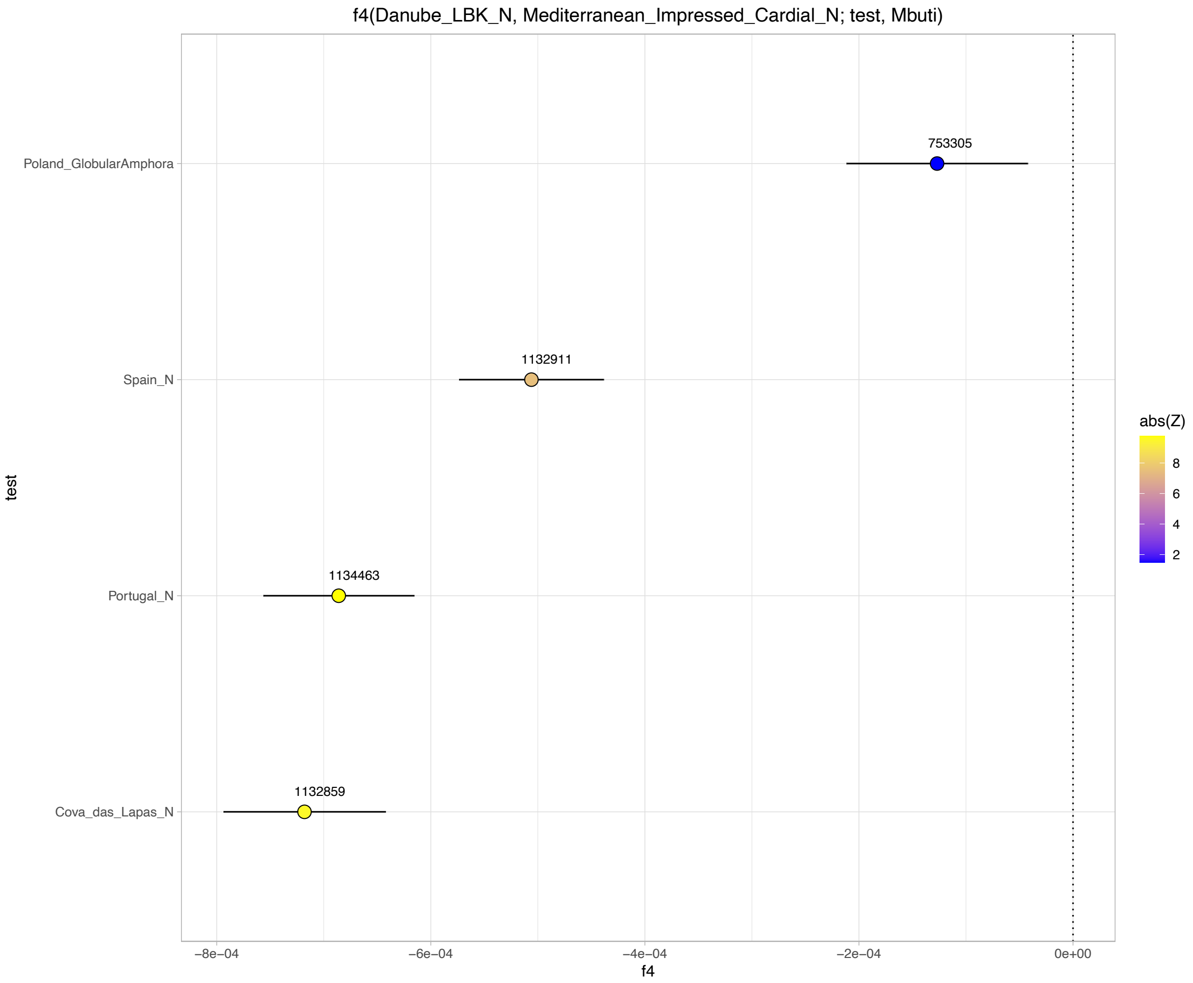

Fig. S7: The x-axes represent f_4-statistic values, with results displayed as the mean ± 1-SD, and colors representing Z-scores. The numbers above each dot indicate the number of SNPs used for each calculation. f_4-statistics of the form f_3(Danube_LBK_N, Mediterranean_Impressed_Cardial_N; test, Mbuti) where *Test* includes Cova_das_Lapas_N, Portugal_N, Spain_N and Poland_GlobularAmphora.

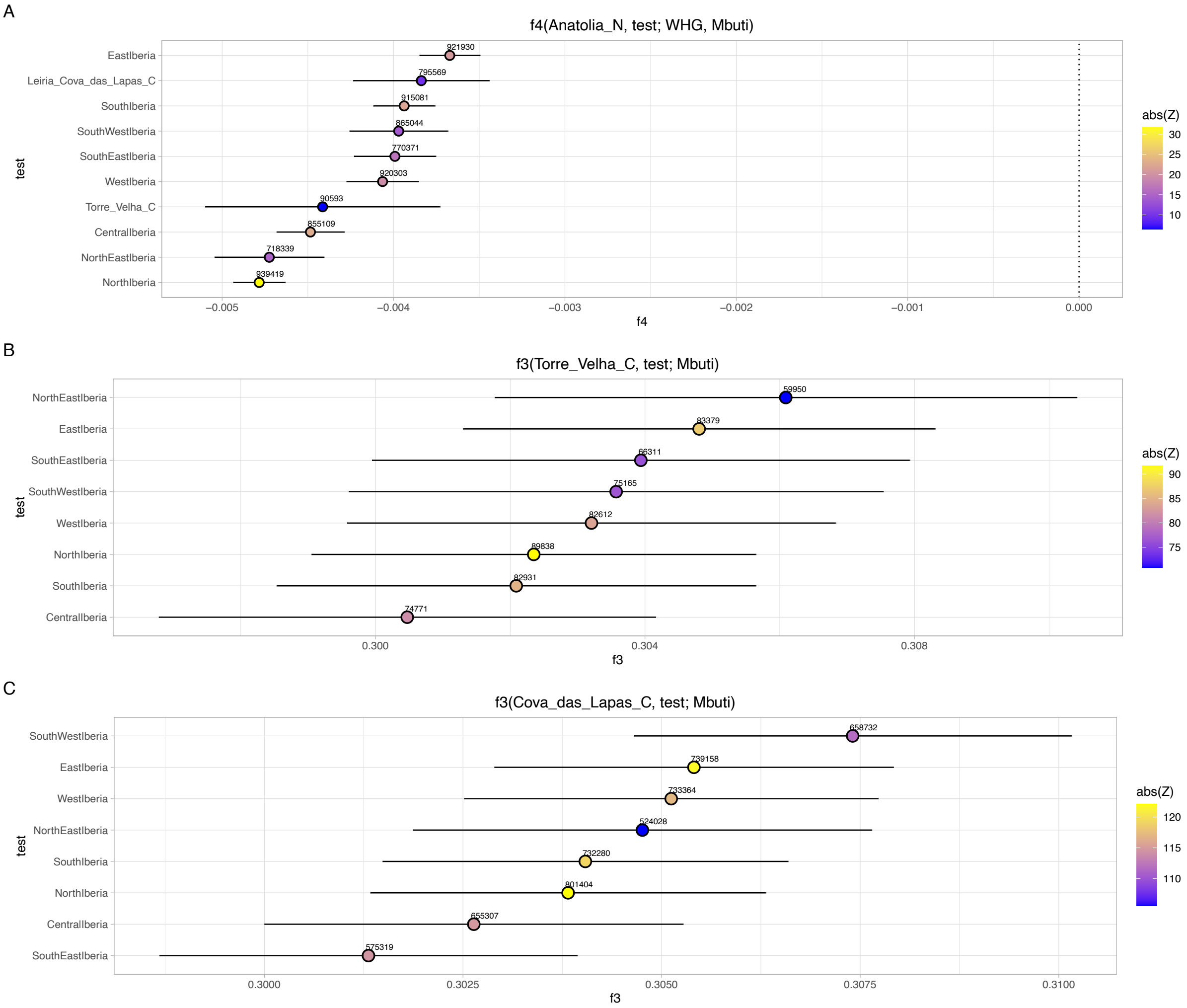

Fig. S8: The x-axes represent the f_3- or f_4-statistic values, with results displayed as the mean ± 1-SD, and colors representing Z-scores. The numbers above each dot indicate the number of SNPs used for each calculation. (A) f_4-statistics in the form f_4 (Anatolia_N, Test; WHG, Mbuti) with *Test* including Cova_das_Lapas_C, Torre_Velha_C and different Iberian Chalcolithic geographical areas. Outgroup f_3-statistics of the form (B) f_3 (Torre_Velha_C, Test; Mbuti) and (C) f_3(Cova_das_Lapas_C, Test; Mbuti), where *Test* includes various Iberian Chalcolithic geographical areas.

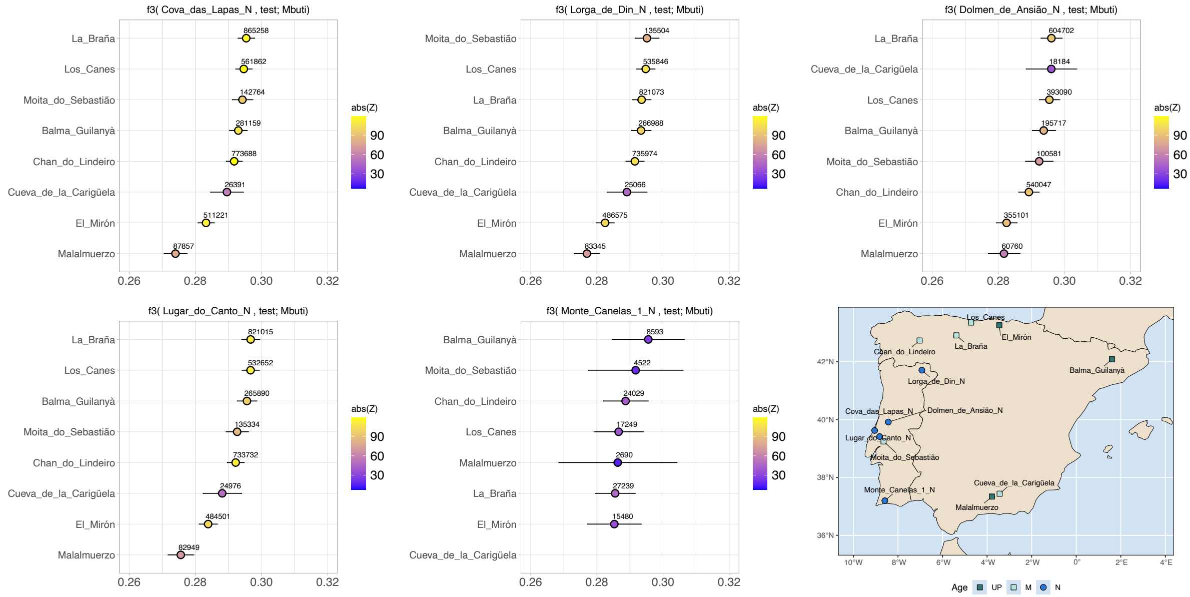

Fig. S9: Outgroup f_3-statistics of the form f_3(X, Test; Mbuti), where X represents different Neolithic archaeological sites from Portugal, and *Test* includes Iberian Upper Paleolithic and Mesolithic hunter-gatherers. The x-axis shows the f_3-statistic values, with results displayed as the mean ± 1-SD and colors representing the Z-scores. The numbers above each dot indicate the number of SNPs used for each calculation. The map represents their geographical location in the peninsula.

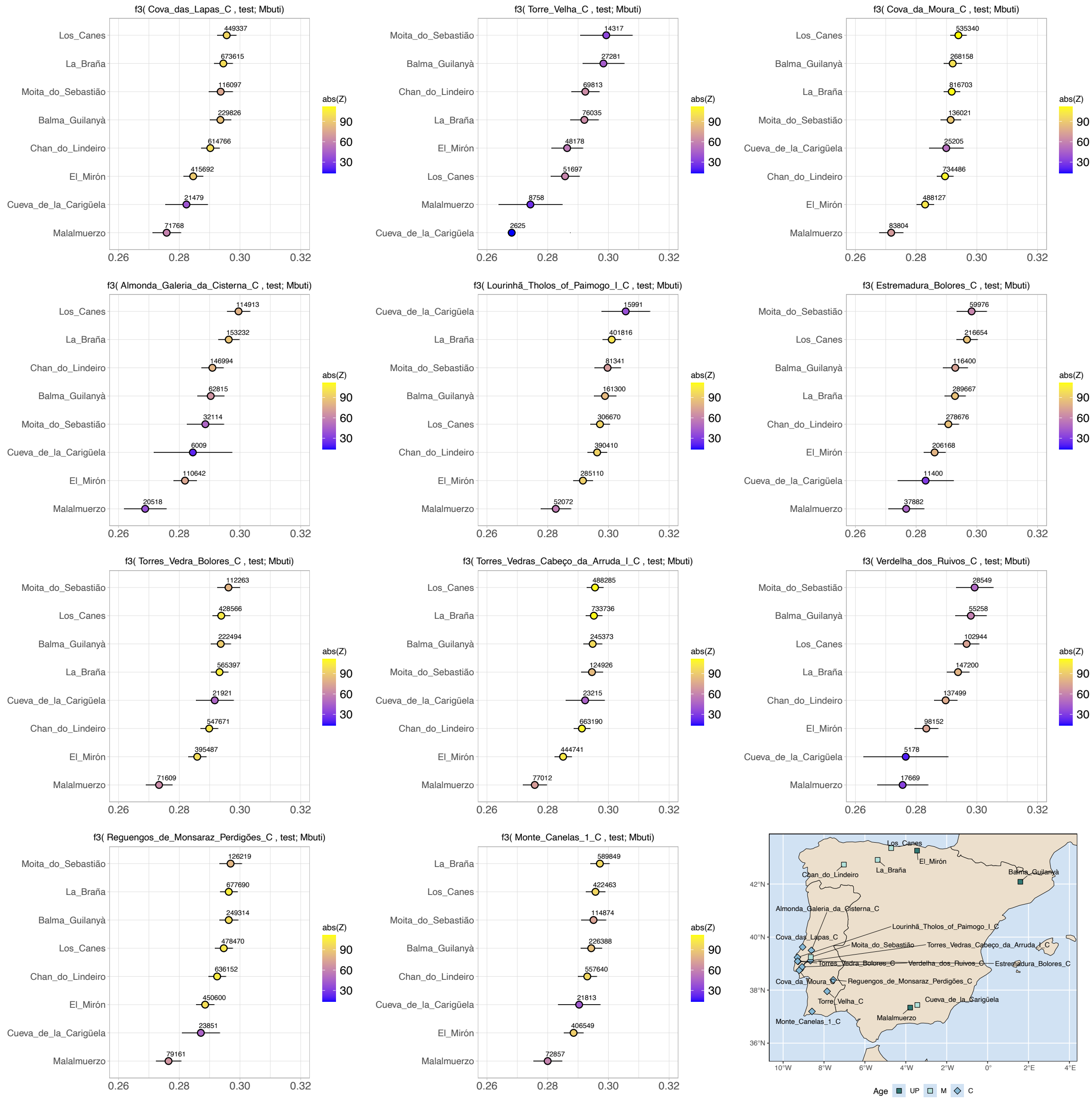

Fig. S10: Outgroup *f*3-statistics of the form *f*3(X, Test; Mbuti), where X represents different Chalcolithic archaeological sites from Portugal, and *Test* includes Iberian Upper Paleolithic and Mesolithic hunter-gatherers. The x-axis shows the *f*3-statistic values, with results displayed as the mean ± 1-SD and colors representing the Z-scores. The numbers above each dot indicate the number of SNPs used for each calculation. The map represents their geographical location in the peninsula.

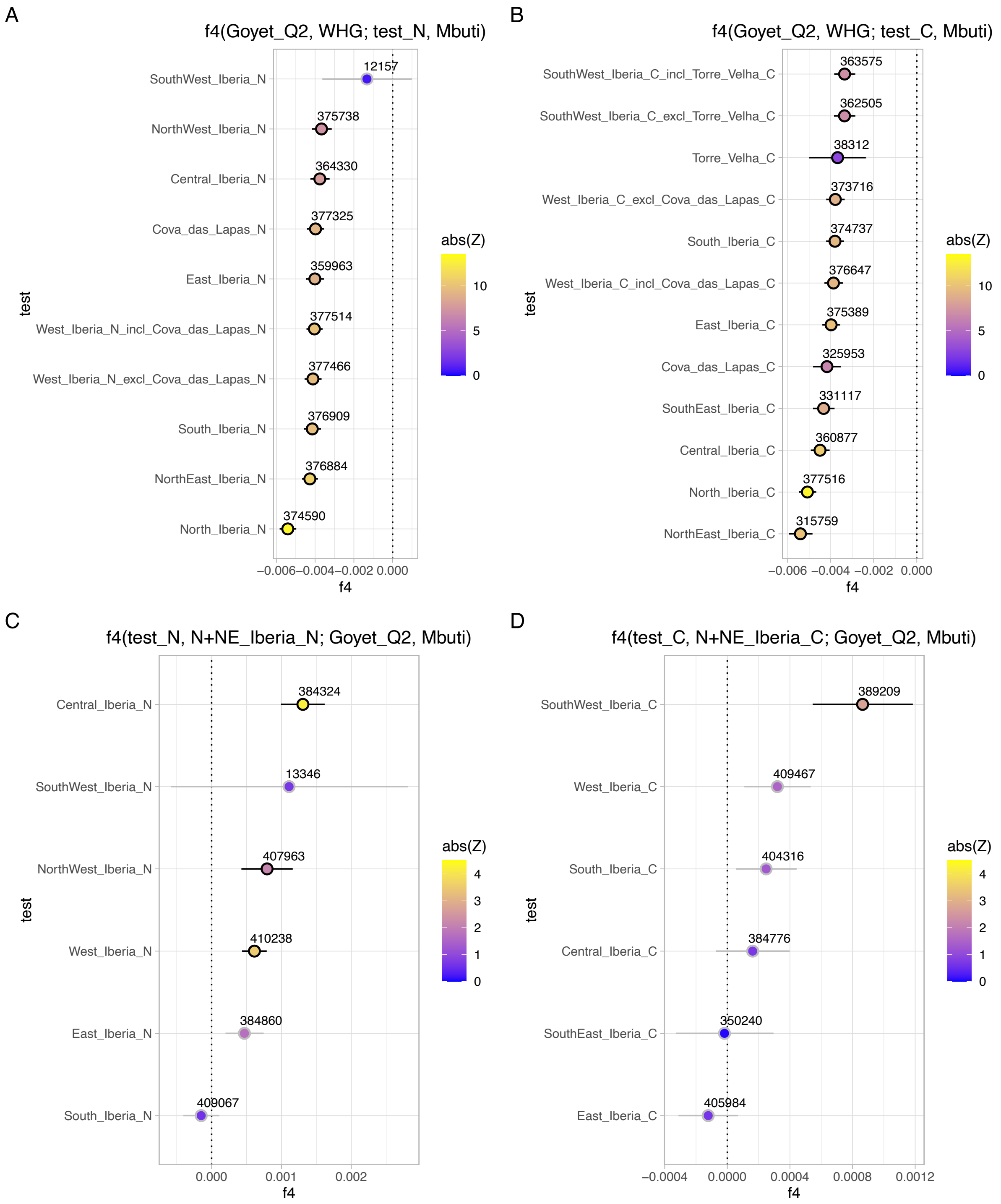

Fig. S11: The x-axes represent f_4-statistic values, with results displayed as the mean ± 1-SD, and colors representing Z-scores. The numbers above each dot indicate the number of SNPs used for each calculation. (A) f_4-statistics of the form f_4(Goyet_Q2, WHG; Test_N, Mbuti) with *Test_N* including Cova_das_Lapas_N and different Iberian Neolithic geographical areas. (B) f_4-statistics of the form f_4(Goyet_Q2, WHG; Test_C, Mbuti) with *Test_C* including Cova_das_Lapas_C, Torre_Velha_C and different Chalcolithic Iberian geographical areas. (C) f_4-statistics of the form f_4(test_N, N+NE_Iberia_N; Goyet_Q2, Mbuti). (D) f_4-statistics of the form f_4(test_C, N+NE_Iberia_C; Goyet_Q2, Mbuti).

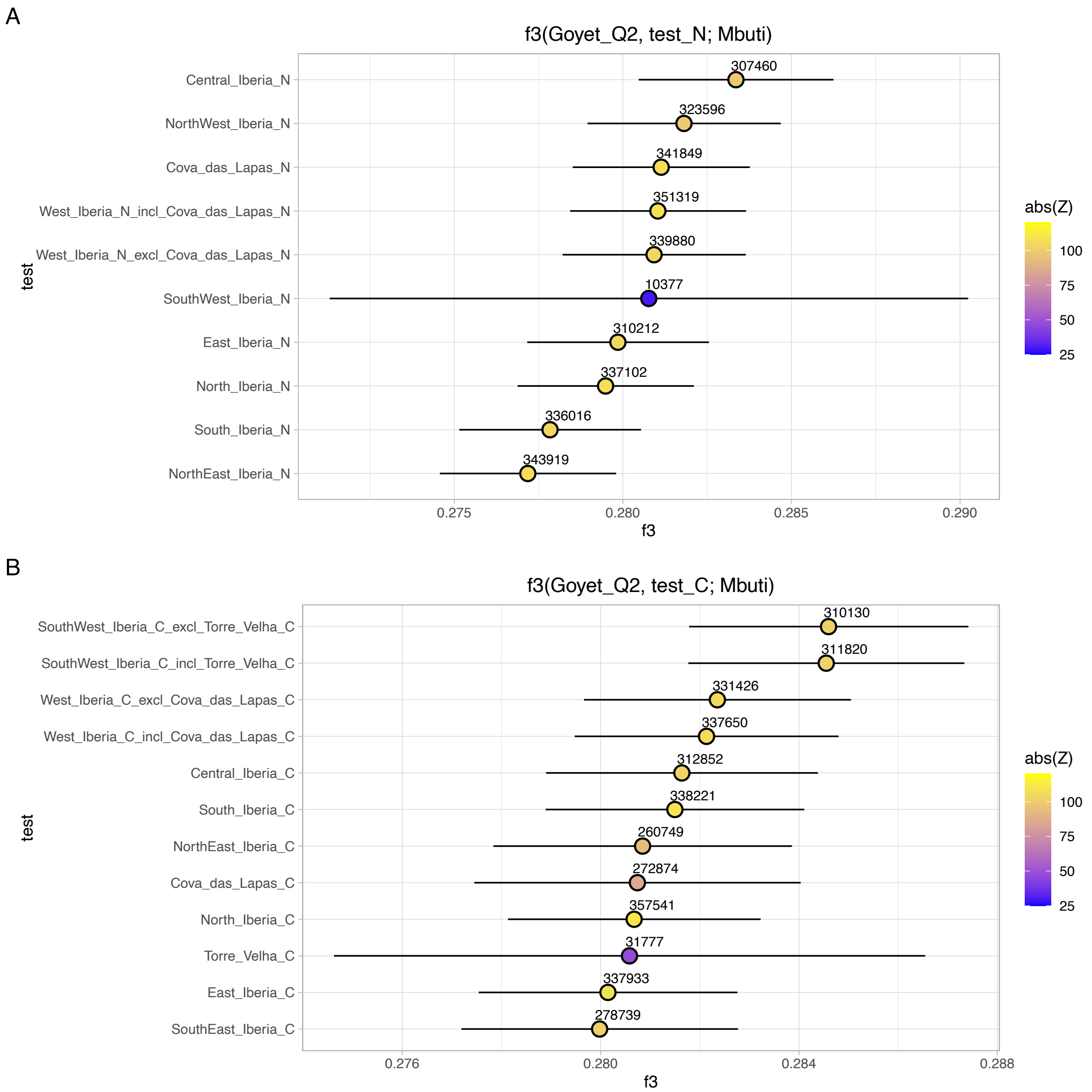

Fig. S12: The x-axes represent f_3-statistic values, with results displayed as the mean ± 1-SD, and colors representing Z-scores. The numbers above each dot indicate the number of SNPs used for each calculation. (A) Outgroup f_3-statistics of the form f_3(GoyetQ2, Test; Mbuti), where *Test* includes Cova_das_Lapas_N and various Iberian Neolithic geographical areas. (B) Outgroup f_3-statistics of the form f_3(GoyetQ2, Test; Mbuti), where *Test* includes Cova_das_Lapas_C, Torre_Velha_C and various Iberian Chalcolithic geographical areas.

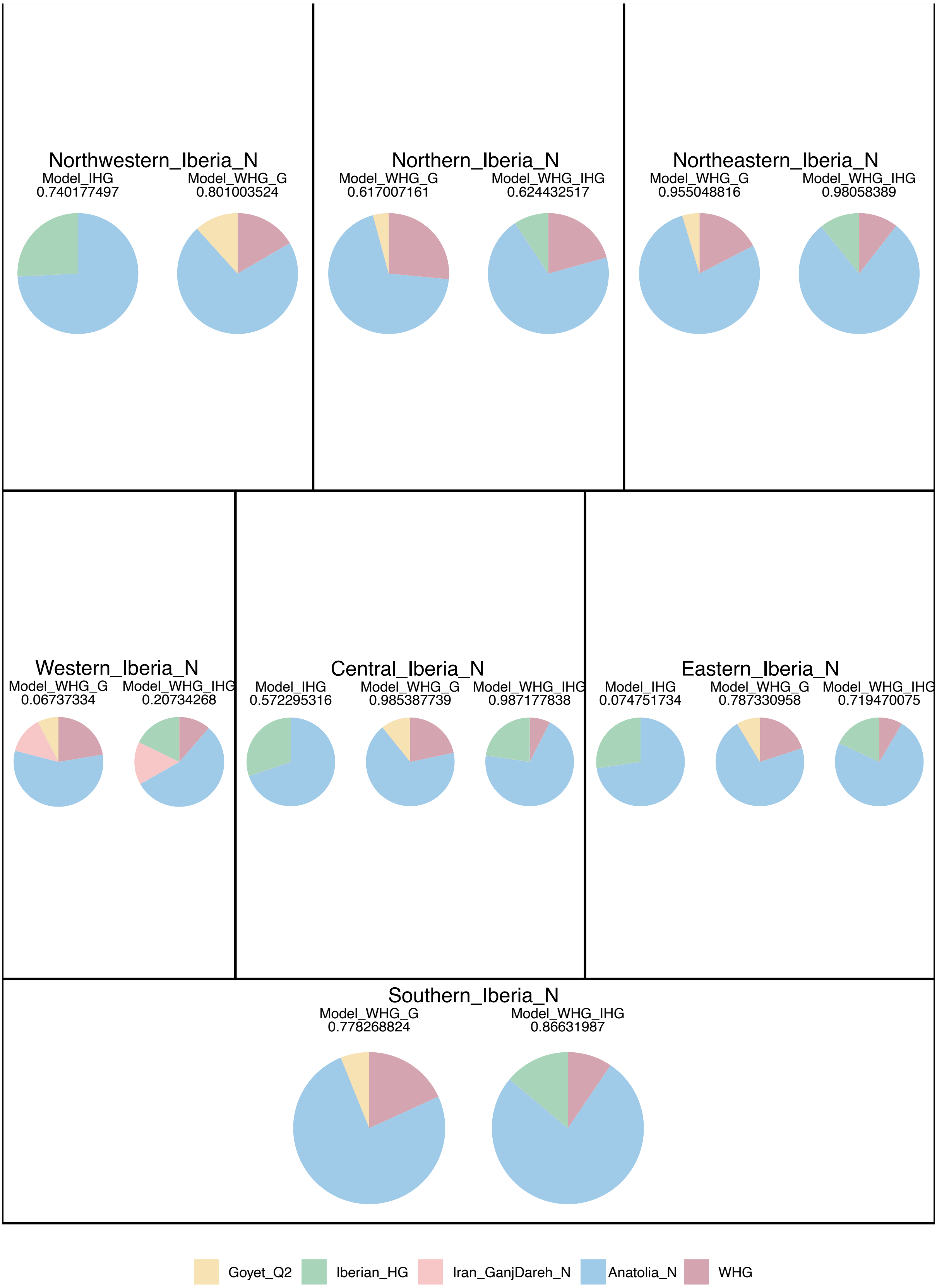

Fig. S13. Ancestry proportions for different geographically defined Neolithic Iberian groups using different admixture modeling frameworks. The p-values are provided below the model label.

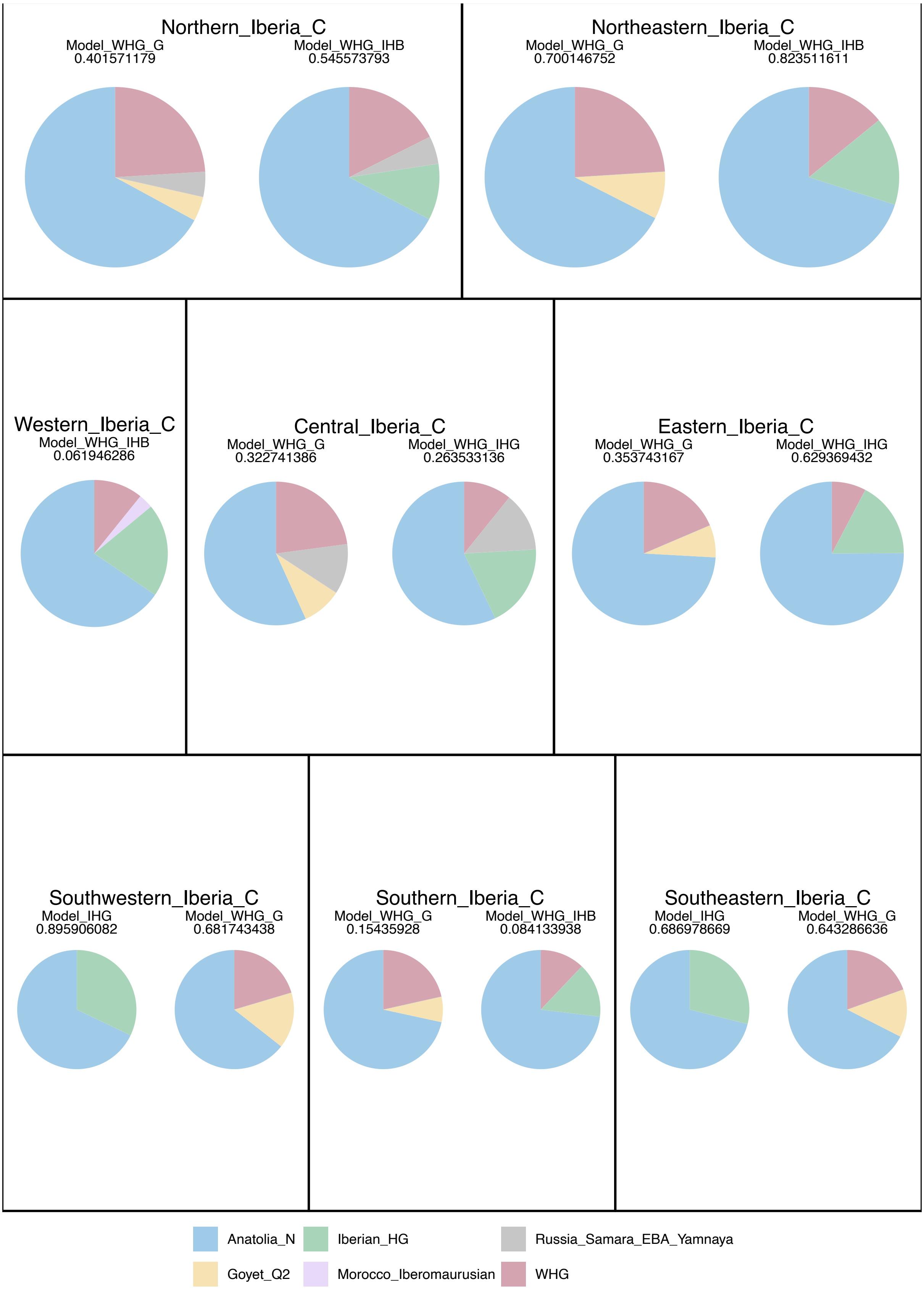

Fig. S14. Ancestry proportions for different geographically defined Chalcolithic Iberian groups using different admixture modeling frameworks. The p-values are provided below the model label.

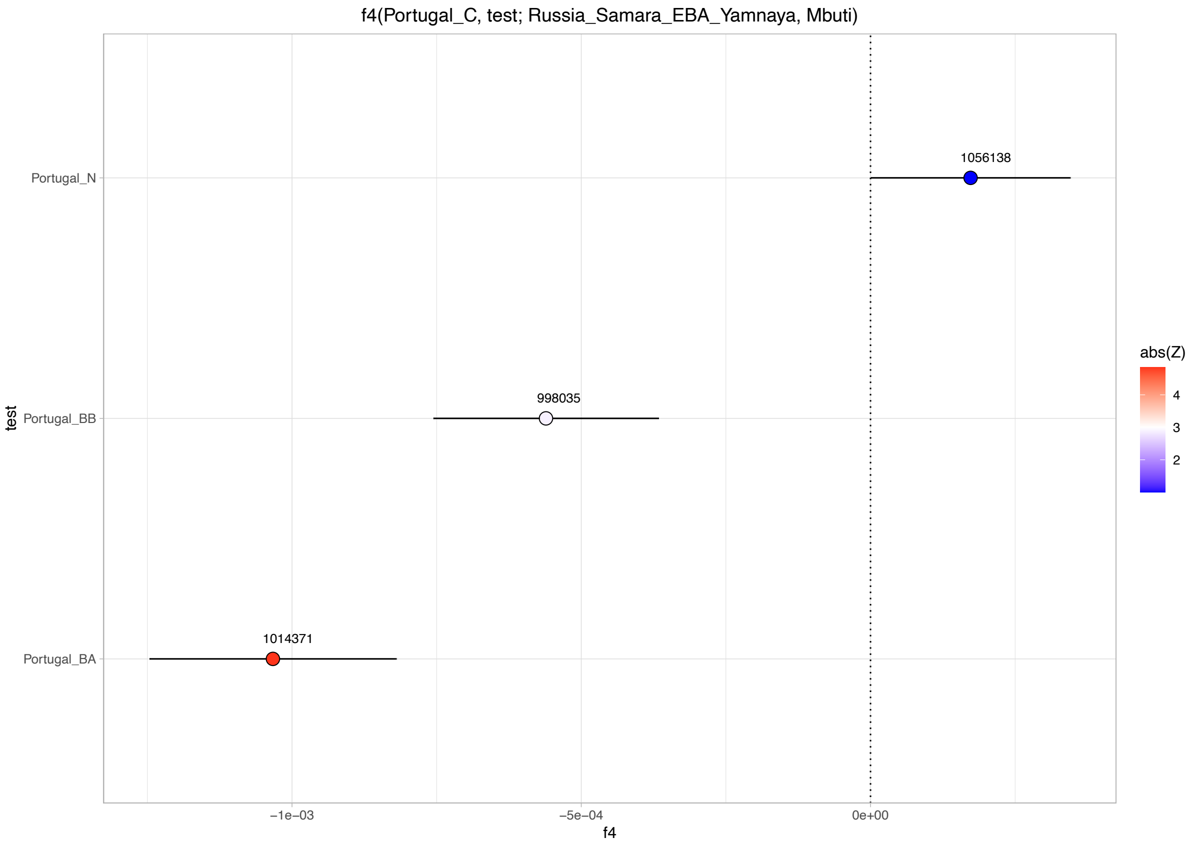

Fig. S15: The x-axes represent f_4-statistic values in the form f_4(Portugal_C, Test; Yamnaya, Mbuti), where *Test* includes Portugal_N, Portugal_BB and Portugal_BA, with results displayed as the mean ± 1-SD, and colors representing Z-scores. The numbers above each dot indicate the number of SNPs used for each calculation.

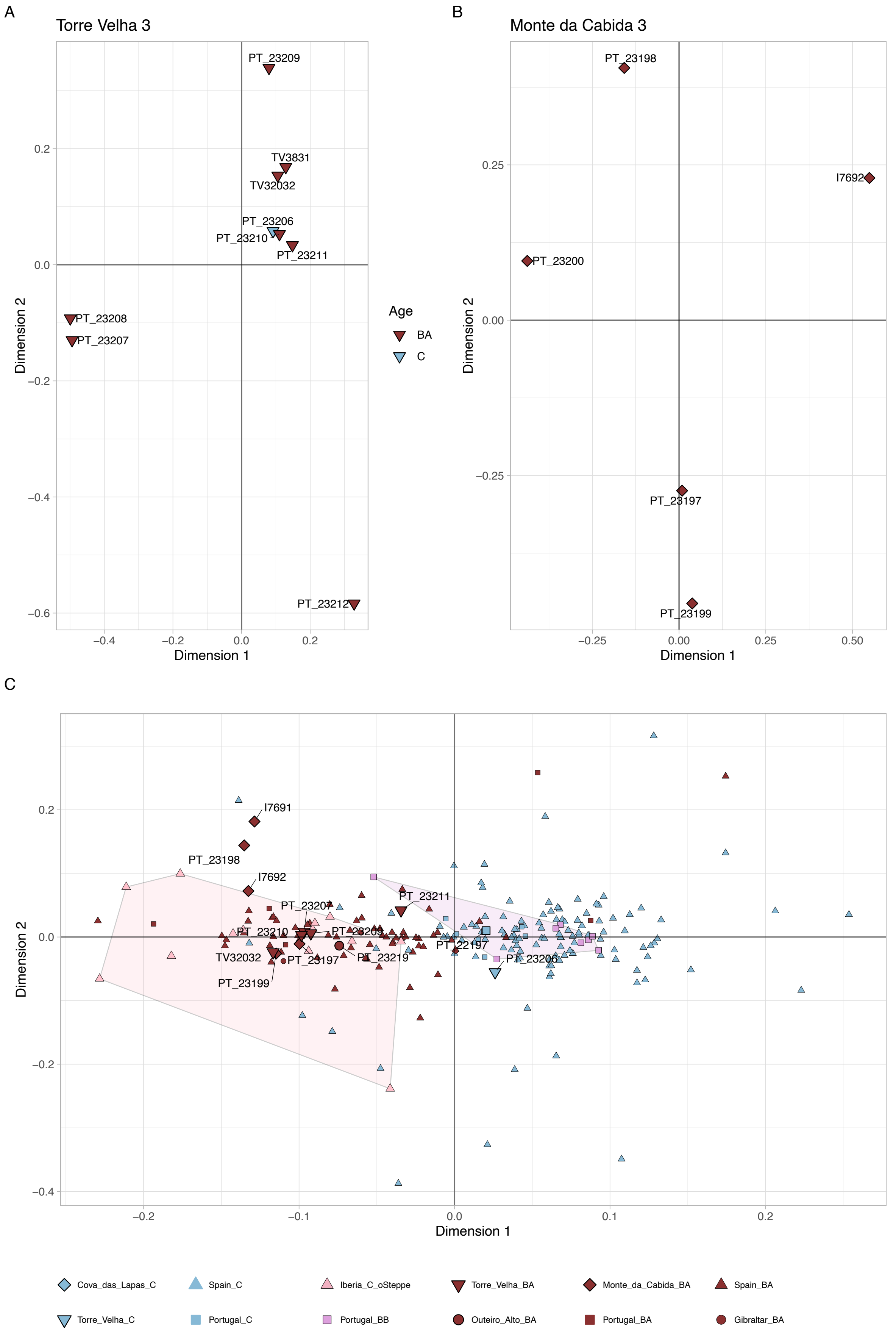

Fig. S16. Multidimensional scaling plot based on 1-f_3(Mbuti; Ind1, Ind2) for the Chalcolithic and Bronze Age individuals from (A) Torre Velha 3, (B) Monte da Cabida 3 and (C) Iberia.

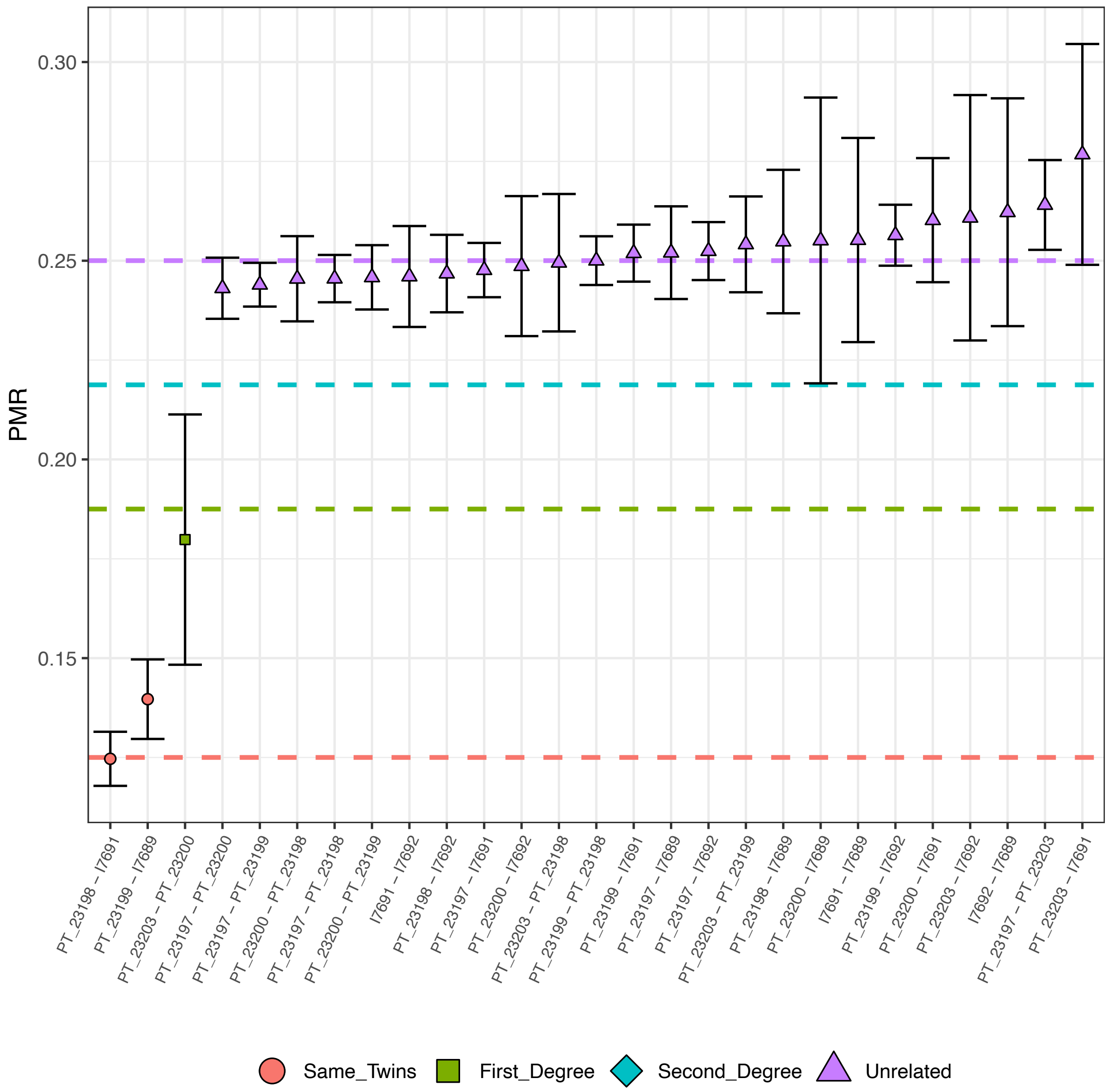

Fig. S17. Kinship analysis from Monte_da_Cabida_BA using BREADR.

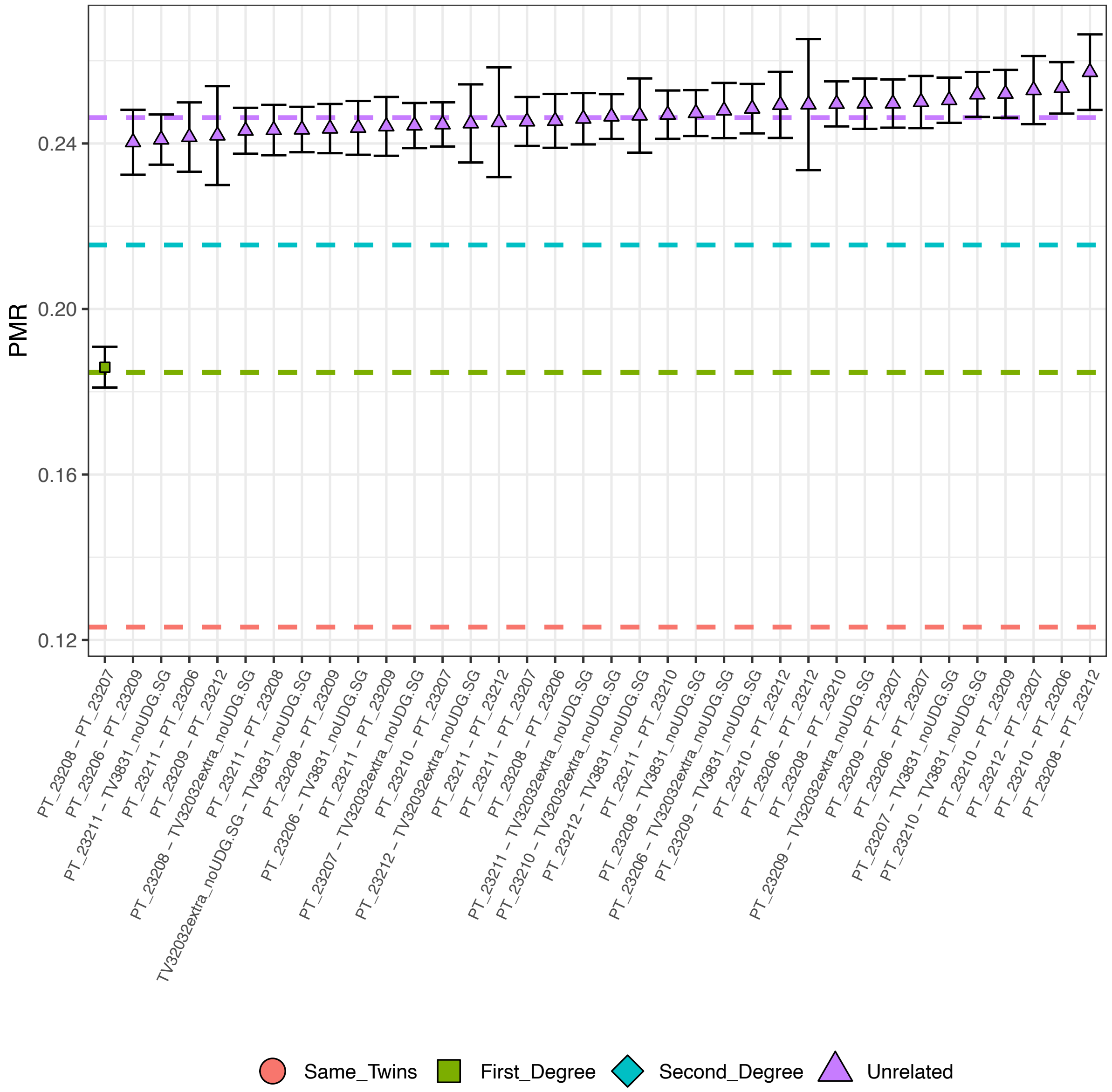

Fig. S18. Kinship analysis from Torre_Velha_C/BA using BREADR.

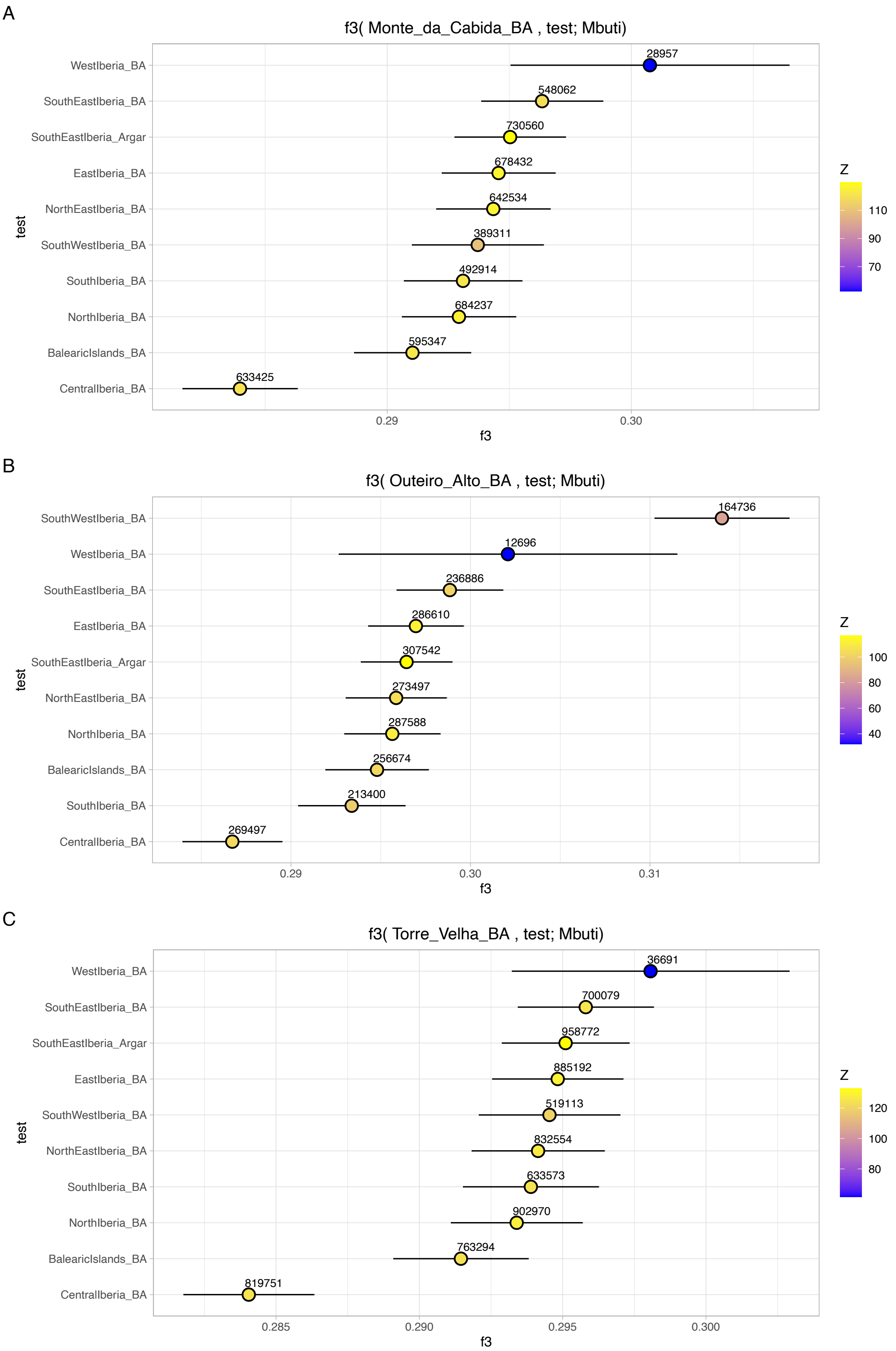

Fig. S19. The x-axes represent the f_3-statistic values, with results displayed as the mean ± 1-SD, and colors representing Z-scores. The numbers above each dot indicate the number of SNPs used for each calculation. Outgroup f_3-statistics of the form (A) f_3(Monte_da_Cabida_BA, Test; Mbuti), (B) f_3(Outeiro_Alto_BA, Test; Mbuti), and (C) f_3(Torre_Velha_BA, Test; Mbuti), where *Test* includes various Iberian Bronze Age geographical areas.

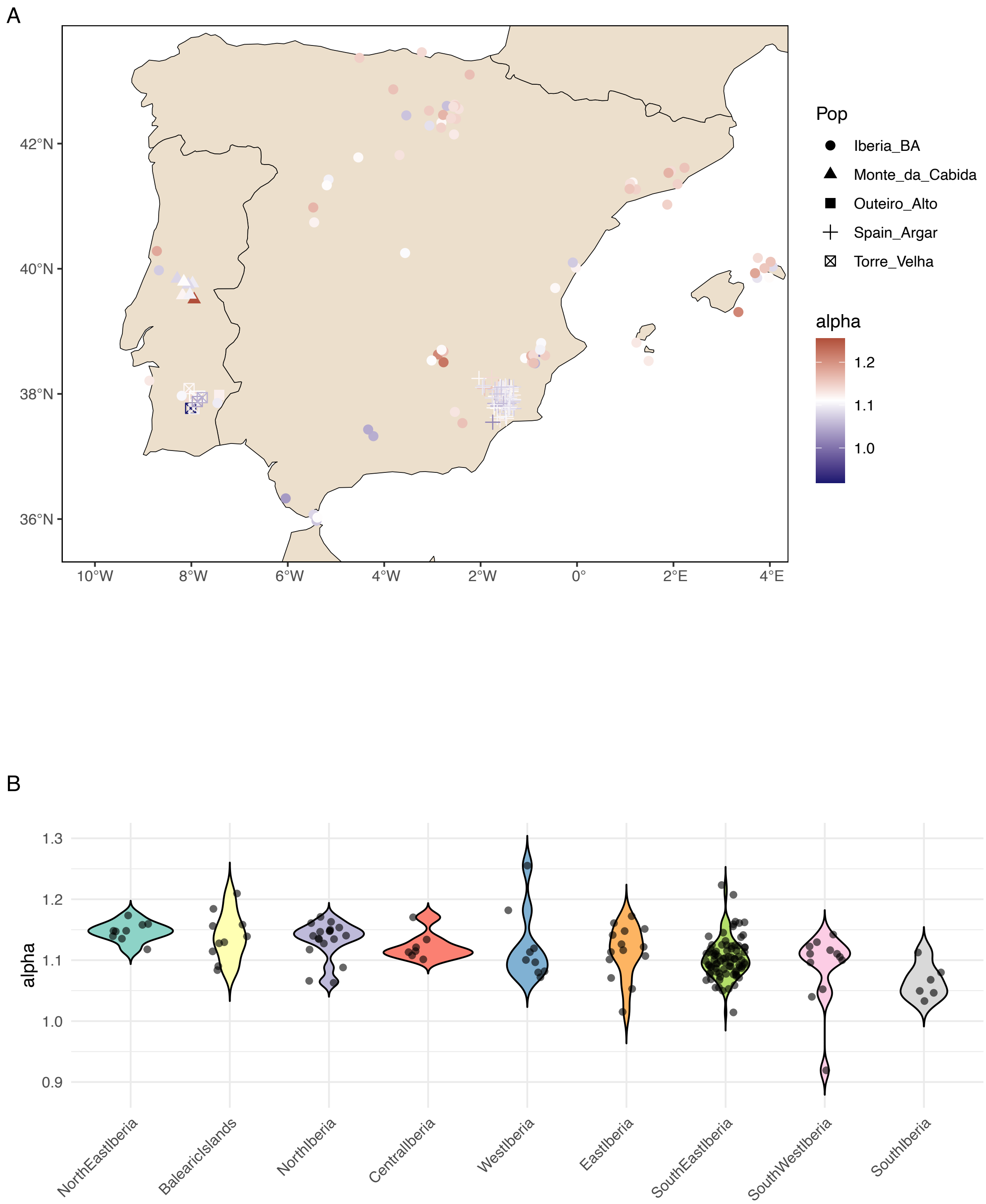

Fig. S20. *f*4-ratios (*f*4(Yamnaya_Samara, Mbuti; Test, Morocco_Iberomaurusian) / *f*4(Yamnaya_Samara, Mbuti; Anatolia_N, Morocco_Iberomaurusian)), where *Test* are (A) Iberian Bronze Age individuals as well as Torre_Velha_BA, Outeiro_Alto_BA and Monte_da_Cabida_BA, or (B) different geographically defined Bronze Age Iberian groups. Individuals with absolute Z-scores higher than twice their standard error were excluded. Most *f*4-ratios exceed 1, consistent with the expectation that *Test* has more Yamnaya_Samara ancestry than Anatolia_N, relative to Morocco_Iberomaurusian. A nuanced decrease in Yamnaya_Samara ancestry is observed southwards in Iberia.

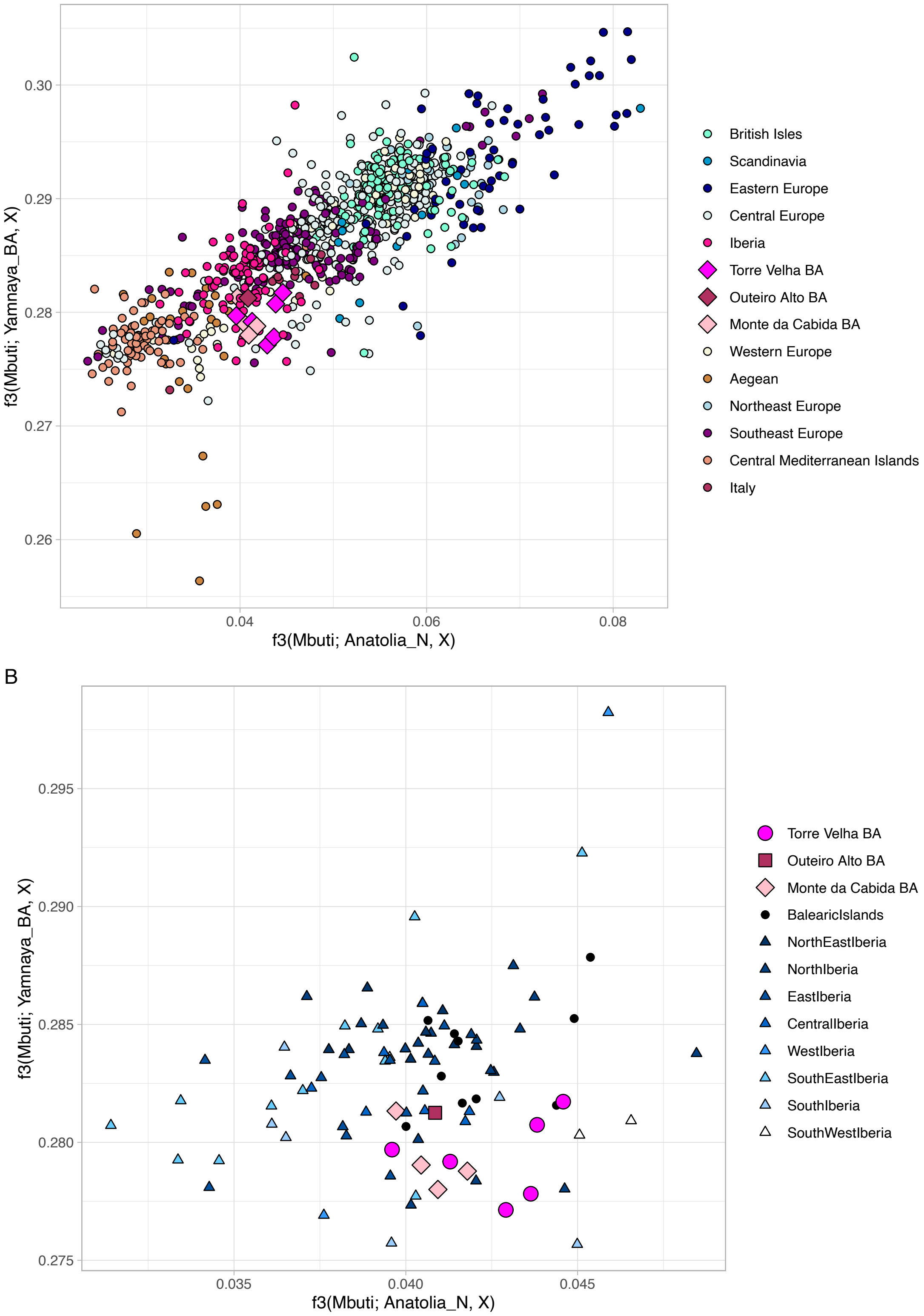

Fig. S21. Outgroup *f3*-statistics of the form f_3(Mbuti; Pop, X) showing the amount of shared drift between the ancient individuals (X) with Neolithic Anatolian Farmers (Anatolia_N; x-axis) and Steppe-related Bronze Age Eurasians (Yamnaya_BA; y-axis). (A) Clusters of Eurasian individuals are represented in shades of blue, pink, and amber from the top-left to the bottom-right. The Torre_Velha_BA, Outeiro_Alto_BA and Monte_da_Cabida_BA individuals are represented as colored diamonds. (B) Clusters of Iberian individuals are represented in shades of blue on a north-south gradient and the Torre_Velha_BA, Outeiro_Alto_BA and Monte_da_Cabida_BA individuals are represented as colored diamonds as in (A).

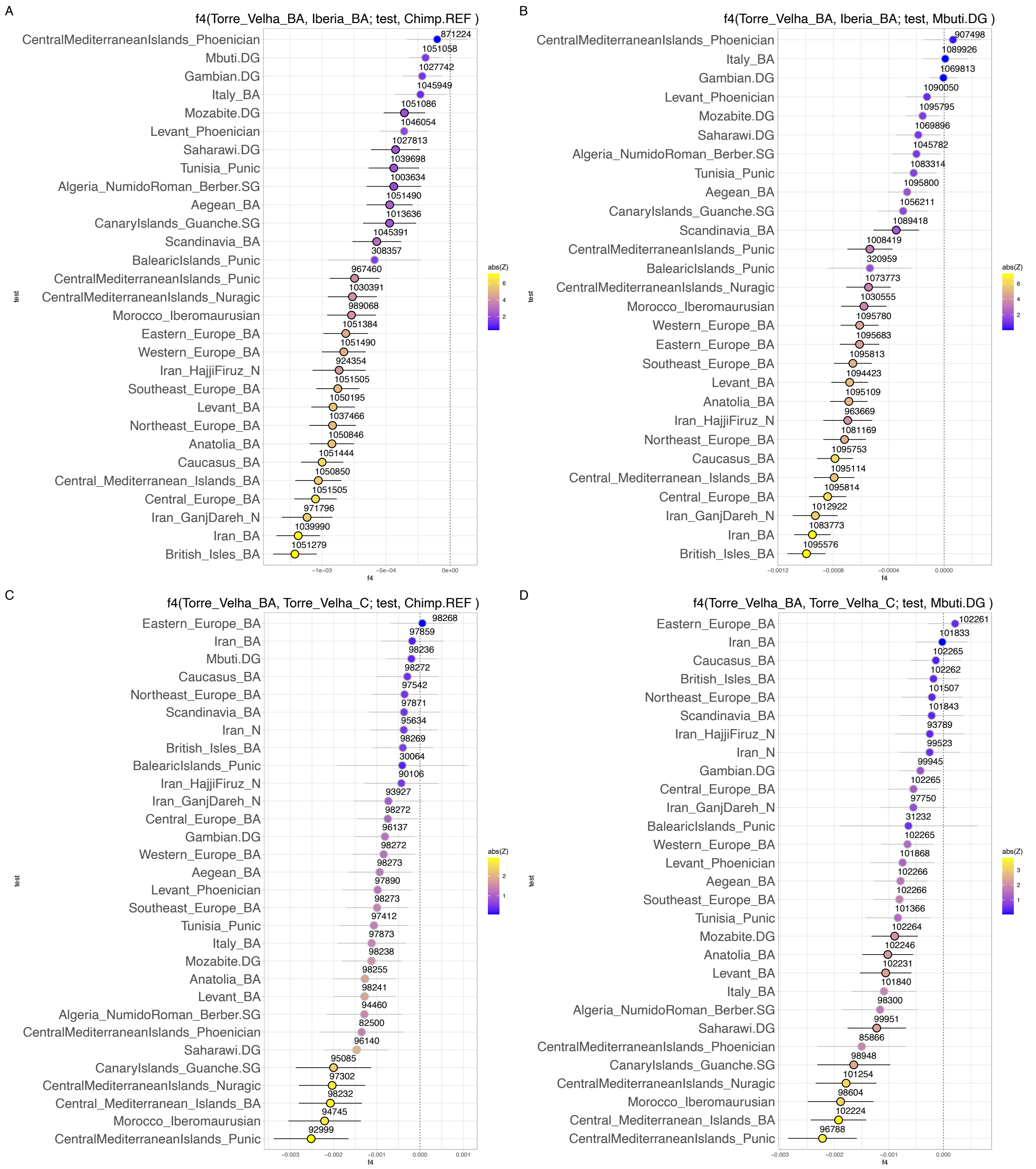

Fig. S22. The x-axes represent the f_4-statistic values, with results displayed as the mean ± 1-SD, and colors representing Z-scores. The numbers above each dot indicate the number of SNPs used for each calculation. f_4-statistic are presented as: (A) f_4(Torre_Velha_BA, Iberia_BA; Test, Mbuti) and (B) f_4(Torre_Velha_BA, Iberia_BA; Test, Chimp), and (C) f_4(Torre_Velha_BA, Torre_Velha_C; Test, Mbuti) and (D) f_4(Torre_Velha_BA, Torre_Velha_C; Test, Chimp), with *Test* including Eurasian and African populations from the Bronze Age or proxies.

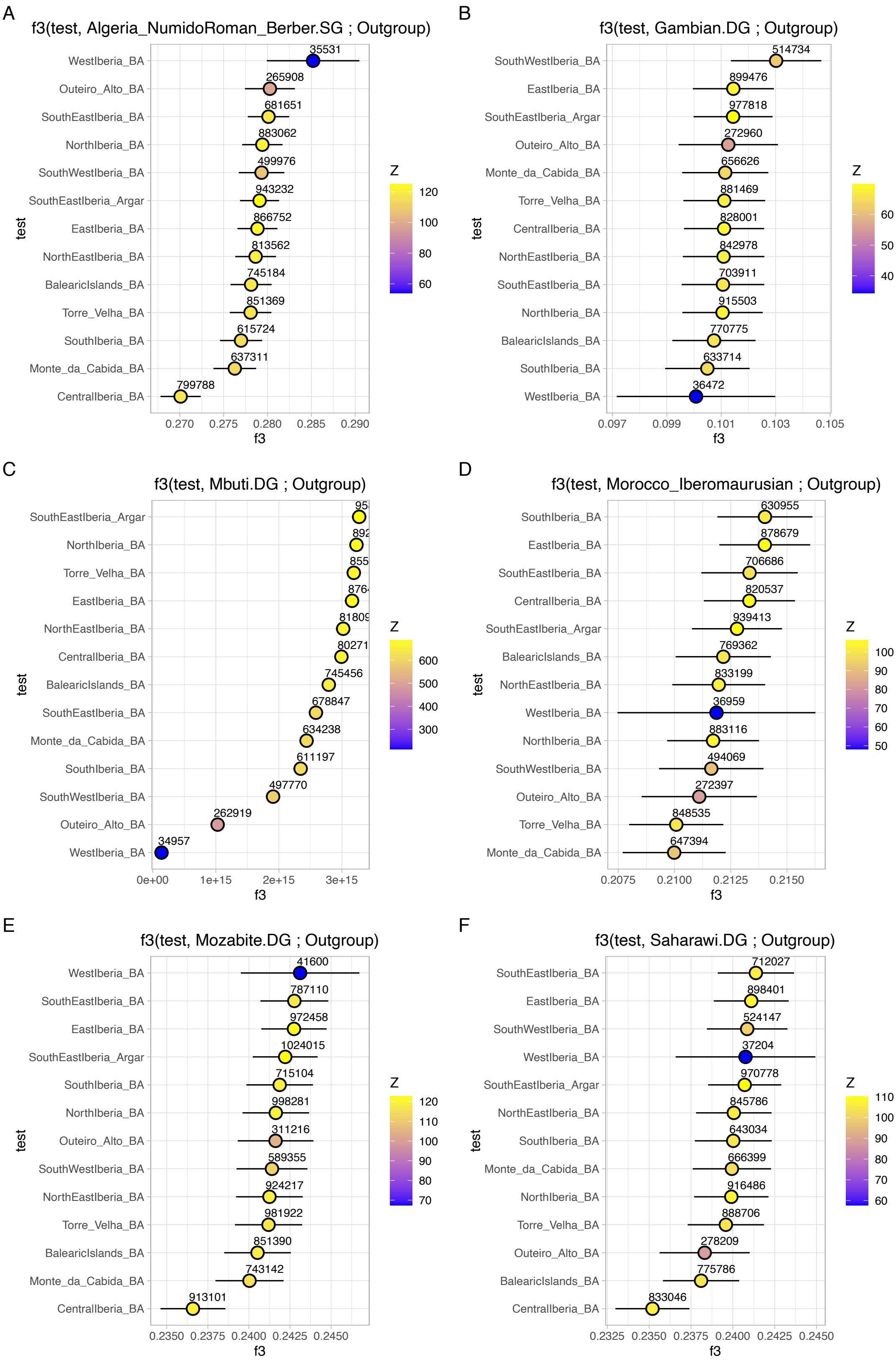

Fig. S23. The x-axes represent the *f3*-statistic values, with results displayed as the mean ± 1-SD, and colors representing Z-scores. The numbers above each dot indicate the number of SNPs used for each calculation. Outgroup f_3-statistics of the form (Test, African_Pop; Outgroup), where *Test* are Torre_Velha_BA, Outeiro_Alto_BA, Monte_da_Cabida_BA and Iberian Bronze Age geographical areas. All outgroups are Mbuti, except when Mbuti is used as *African_Pop*, then the Outgroup is Chimp.

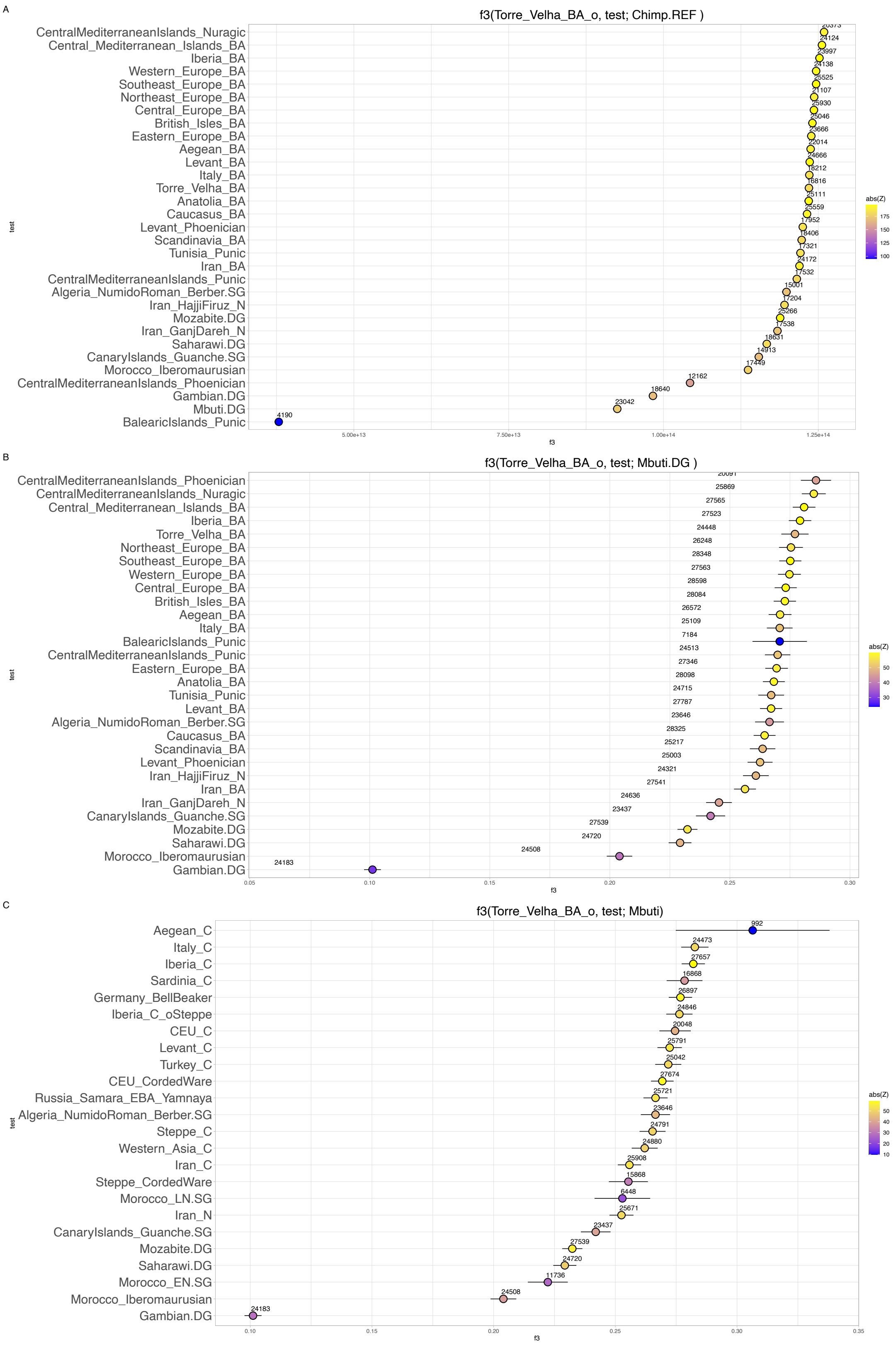

Fig. S24. The x-axes represent the f_3-statistic values, with results displayed as the mean ± 1-SD. The numbers above each dot indicate the number of SNPs used for each calculation. Outgroup f_3-statistics are presented as: (A) f_3 (Torre_Velha_BA_o, Test; Chimp) and (B) f_3 (Torre_Velha_BA_o, Test; Mbuti), with *Test* including Eurasian and African populations from the Bronze Age or proxies; and (C) f_3(Torre_Velha_BA_o, Test; Mbuti), with *Test* including Eurasian and African populations from the Chalcolithic or proxies.

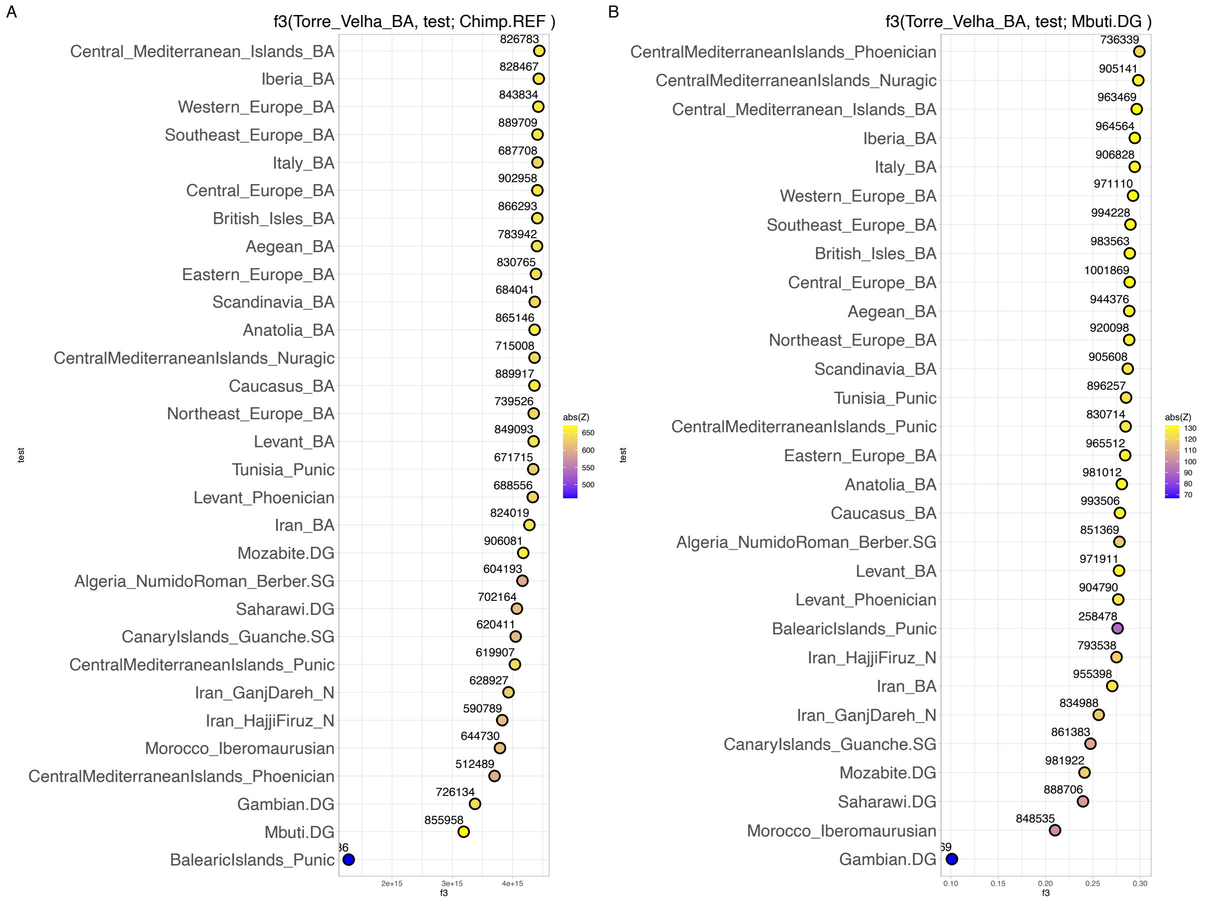

Fig. S25. The x-axes represent the f_3-statistic values, with results displayed as the mean ± 1-SD, and colors representing Z-scores. The numbers above each dot indicate the number of SNPs used for each calculation. Outgroup f_3-statistics are presented as: (A) f_3(Torre_Velha_BA, Test; Mbuti) and (B) f_3(Torre_Velha_BA, Test; Chimp), with *Test* including Eurasian and African populations from the Bronze Age or proxies.

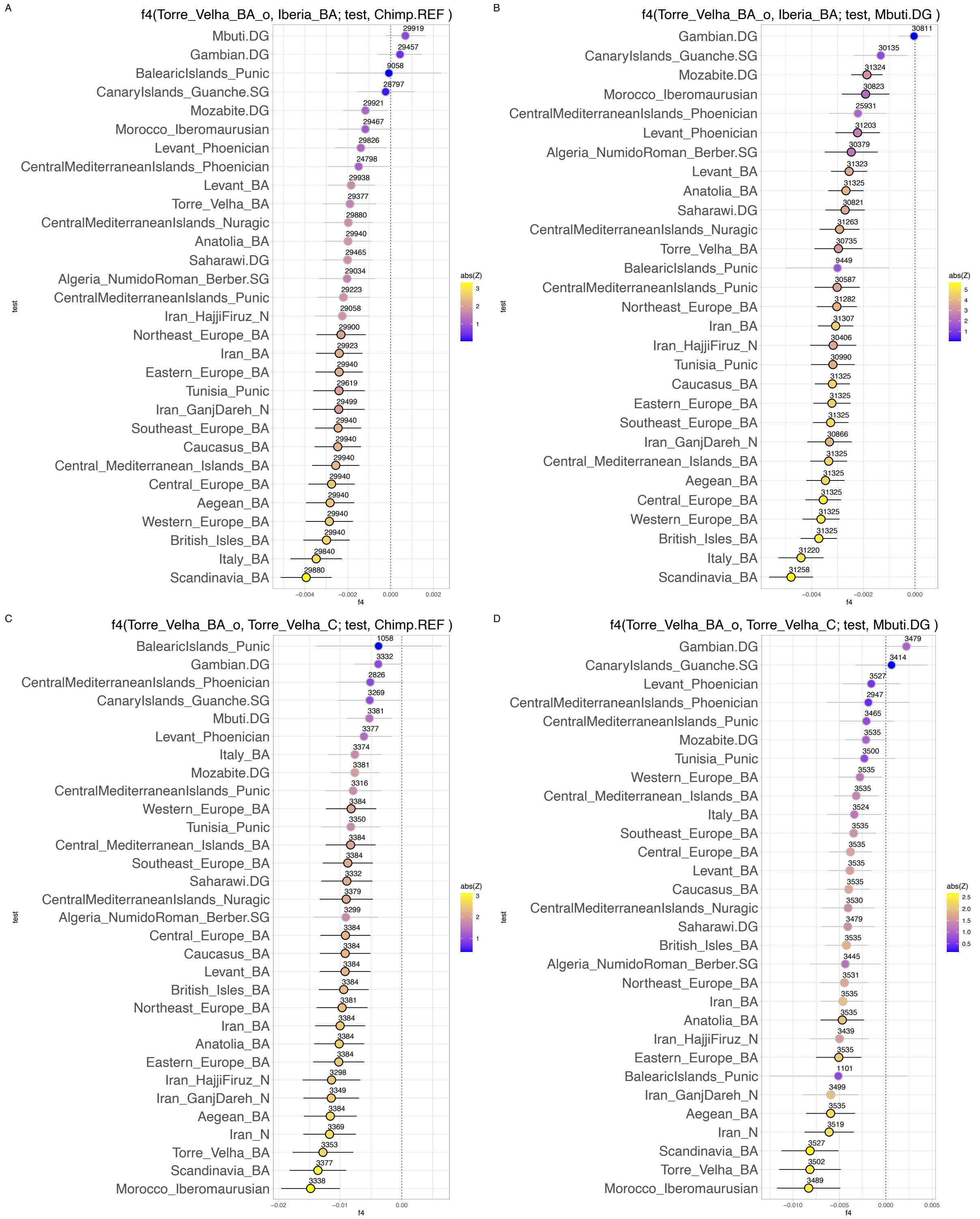

Fig. S26. The x-axes represent the f_4-statistic values, with results displayed as the mean ± 1-SD, colors representing Z-scores and black strokes Z-scores > 2. The numbers above each dot indicate the number of SNPs used for each calculation. f_4-statistics are shown as: (A) f_4(Torre_Velha_BA_o, Iberia_BA; Test, Chimp) and (B) f_4(Torre_Velha_BA_o, Iberia_BA; Test, Mbuti), and (C) f_4(Torre_Velha_BA_o, Torre_Velha_C; Test, Chimp) and (D) f_4(Torre_Velha_BA_o, Torre_Velha_C; Test, Mbuti), with *Test* including Eurasian and African populations from the Bronze Age or proxies.

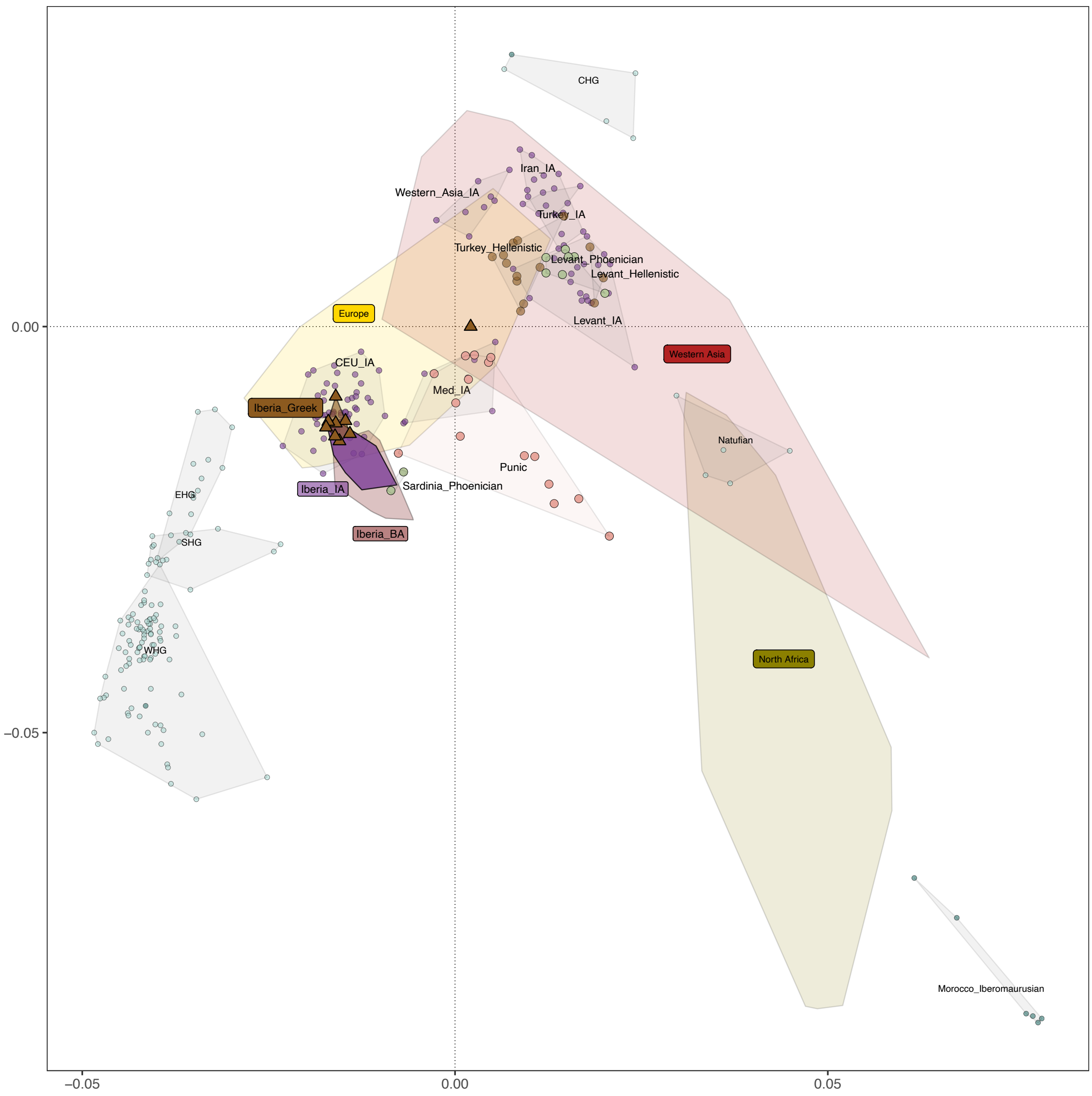

Fig. S27. PCA of present-day West Eurasians and North Africans (overlaid colored polygons represent geographical clusters) with ancient individuals from Iberia and other regions projected onto the first two principal components, focusing on the Iron Age. Colors correspond to different temporal periods, as shown in 1B as well as Punic populations in purple and Phoenician in light green.

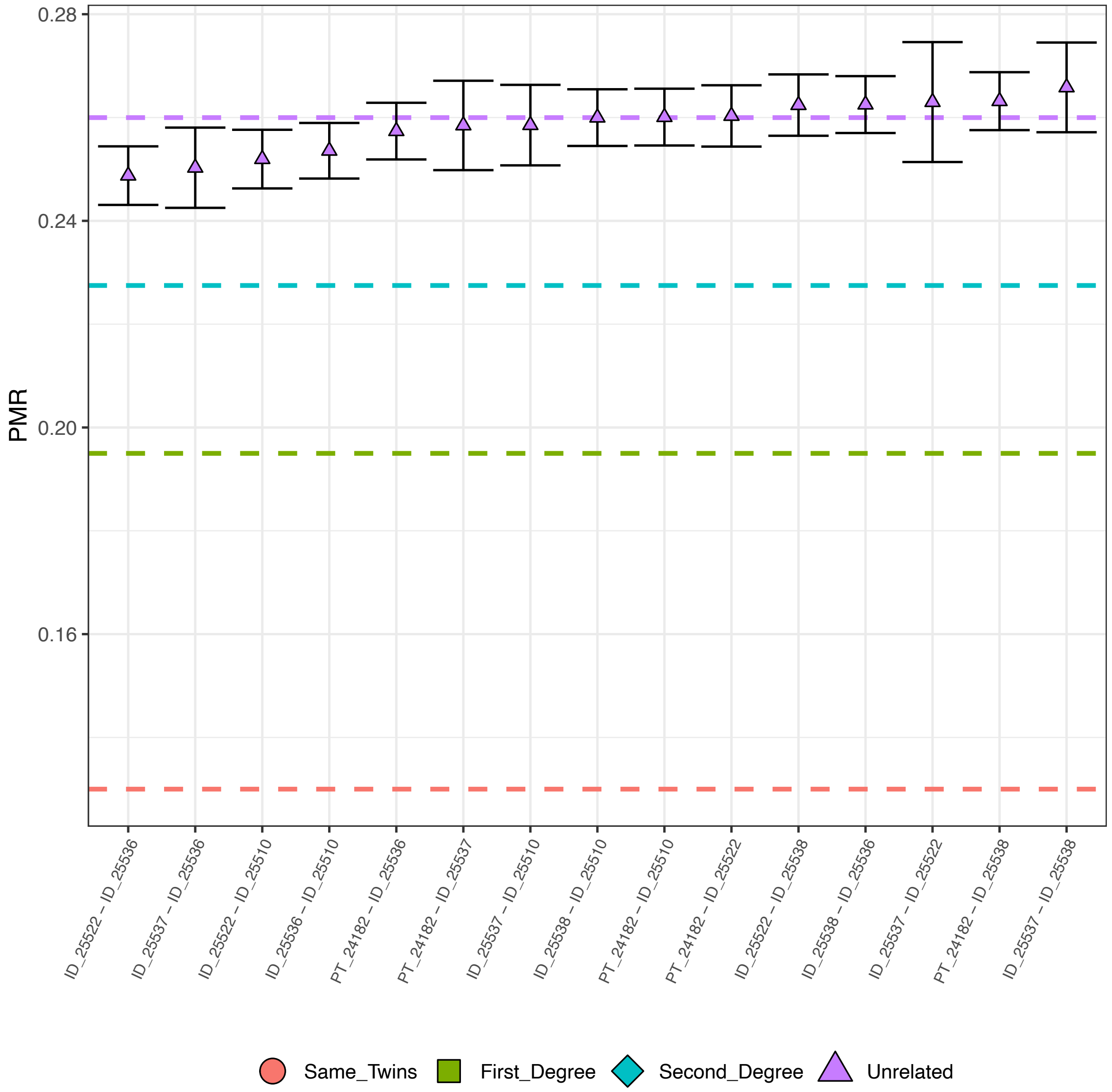

Fig. S28. Kinship analysis from Idanha_a_Velha_Roman/EarlyMedieval using BREADR.

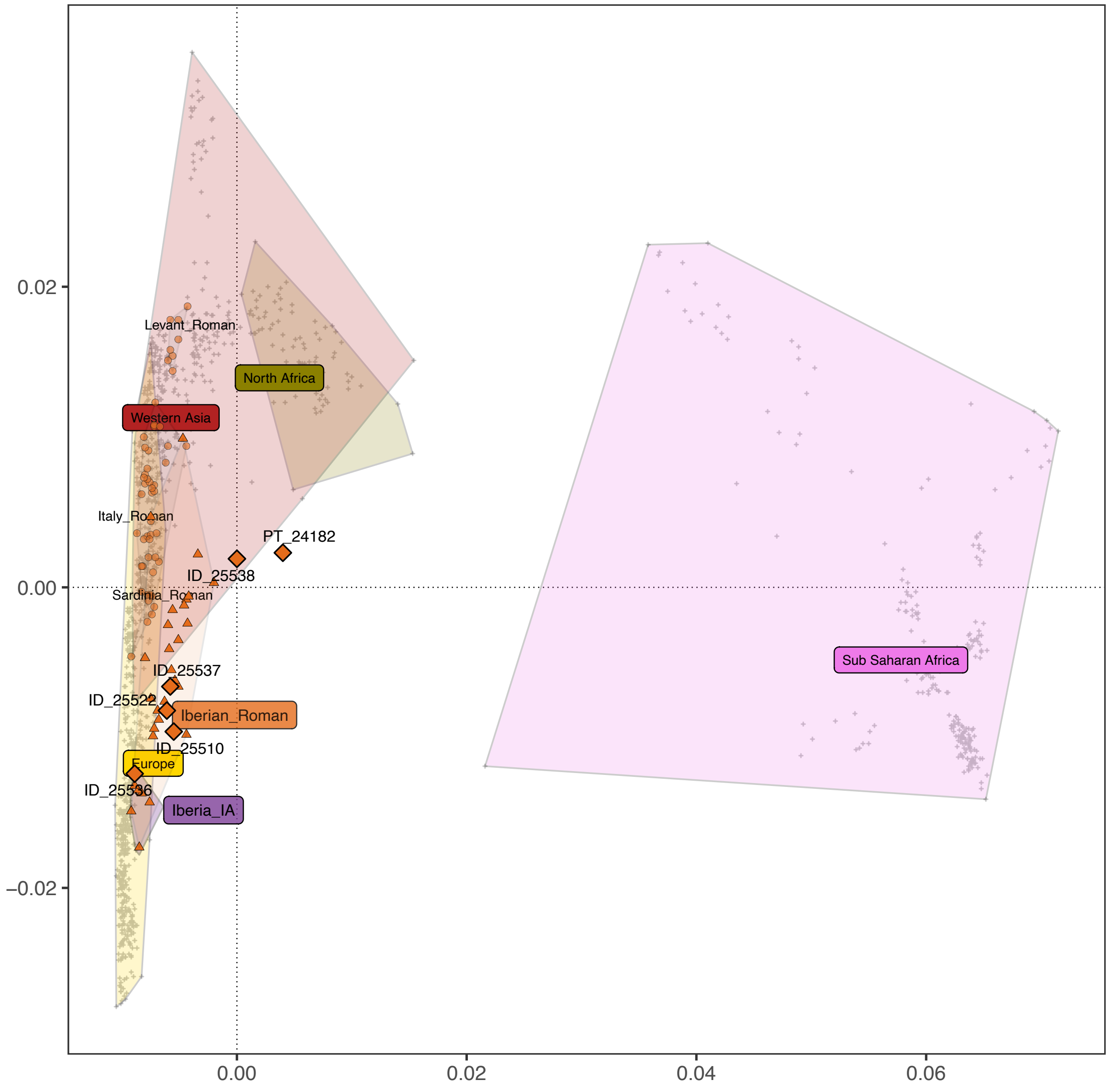

Fig. S29. PCA of present-day West Eurasians, North Africans and Sub-Saharan Africans (overlaid colored polygons represent geographical clusters) with ancient individuals from Iberia and other regions projected onto the first two principal components, focusing on the Roman period. Colors correspond to different temporal periods, as shown in 1B.

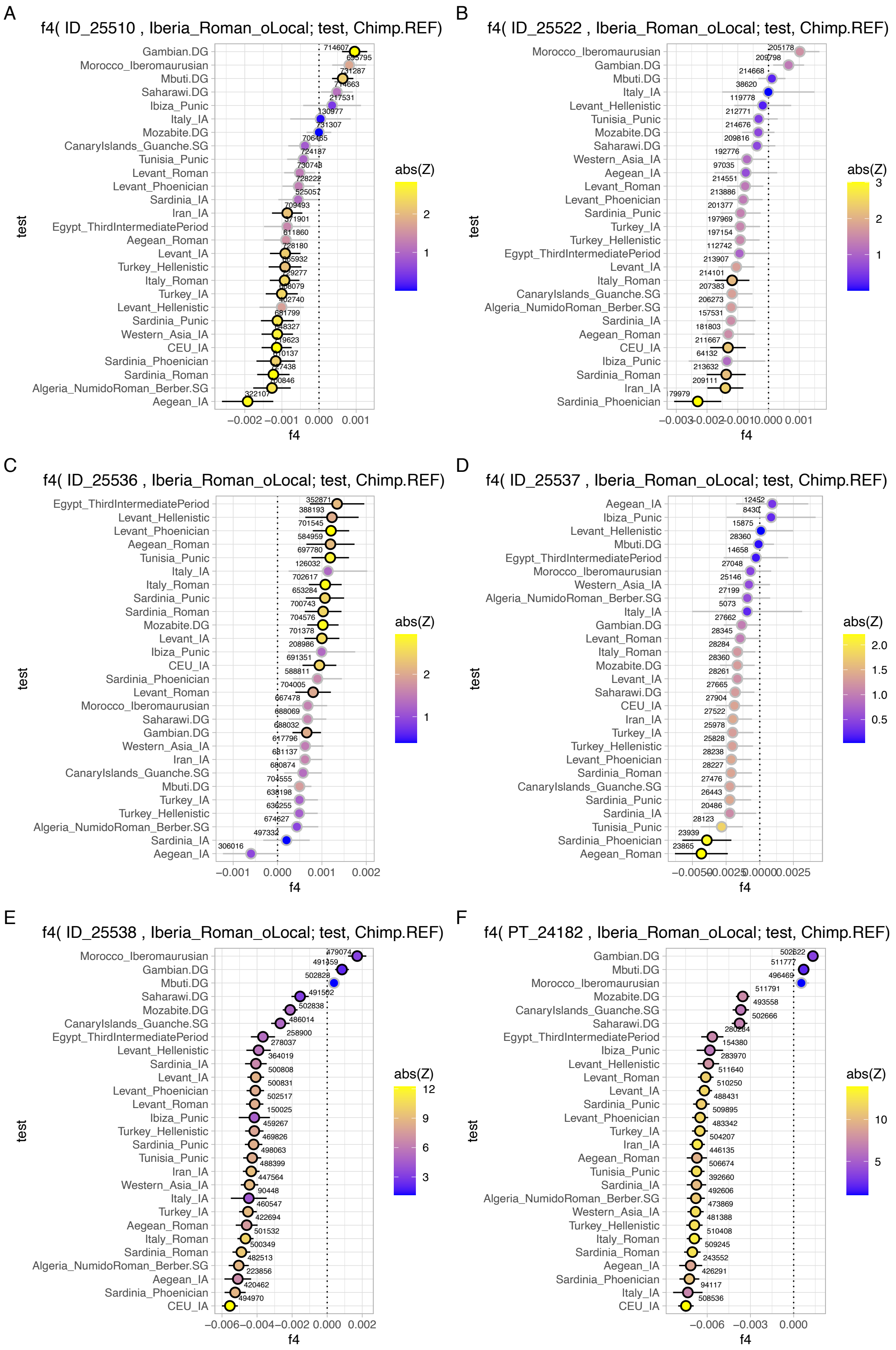

Fig. S30. The x-axes represent the f_4-statistic values, with results displayed as the mean ± 1-SD, colors representing Z-scores and black strokes Z-scores > 2. The numbers above each dot indicate the number of SNPs used for each calculation. f_4-statistics are shown as f_4(X, Iberia_Roman_oLocal; Test, Chimp) with *Test* including Eurasian and African populations from the Roman period or proxies and *X* being (A) ID_25510, (B) ID_25522, (C) ID_25536, (D) ID_25537, (E) ID_25538 and (F) PT_24182.

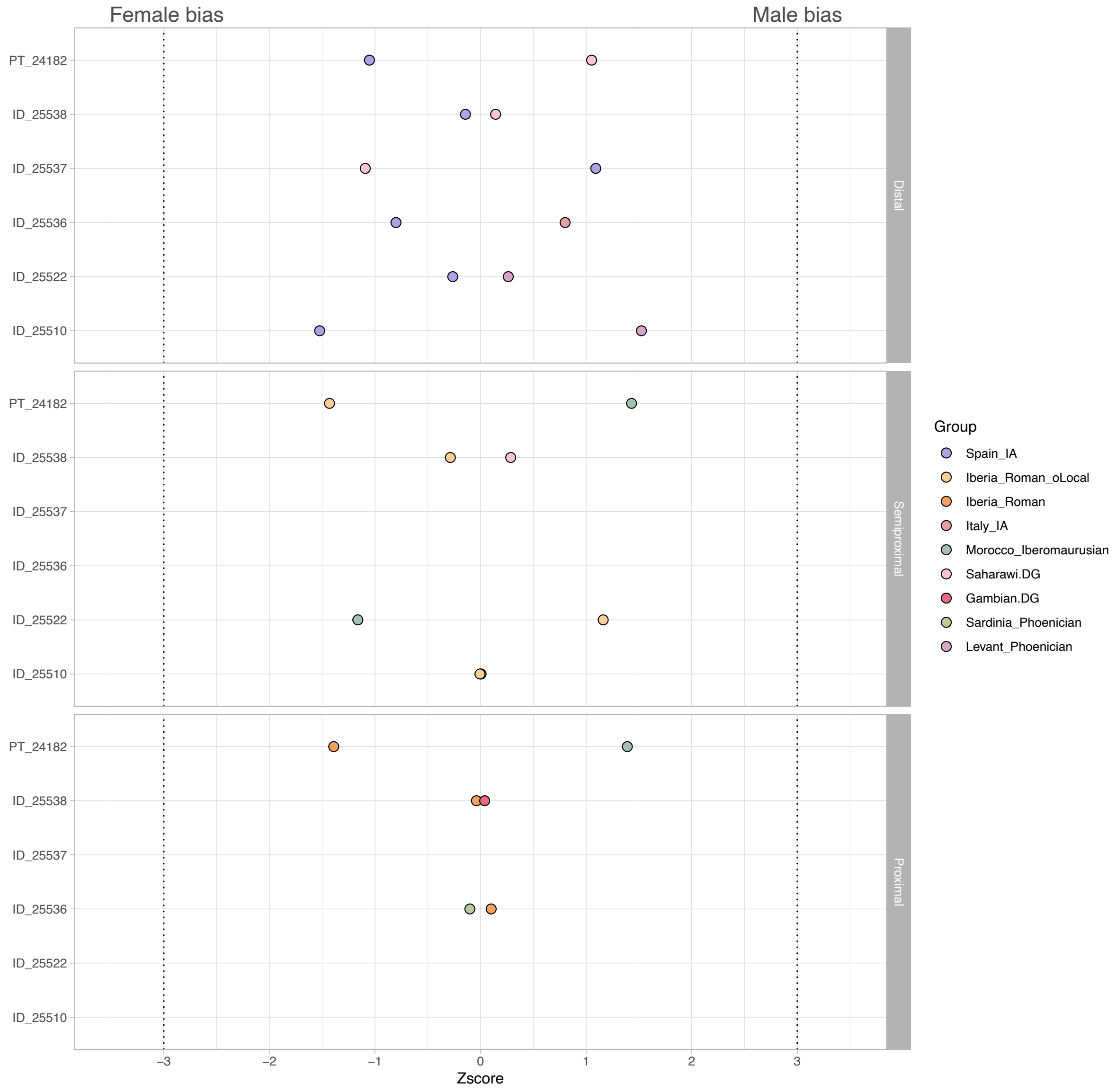

Fig. S31. *qpAdm* Z scores between autosomes and the X chromosome showing no signal for sex bias in any ancestry. Refer to Fig. 3D for further information.

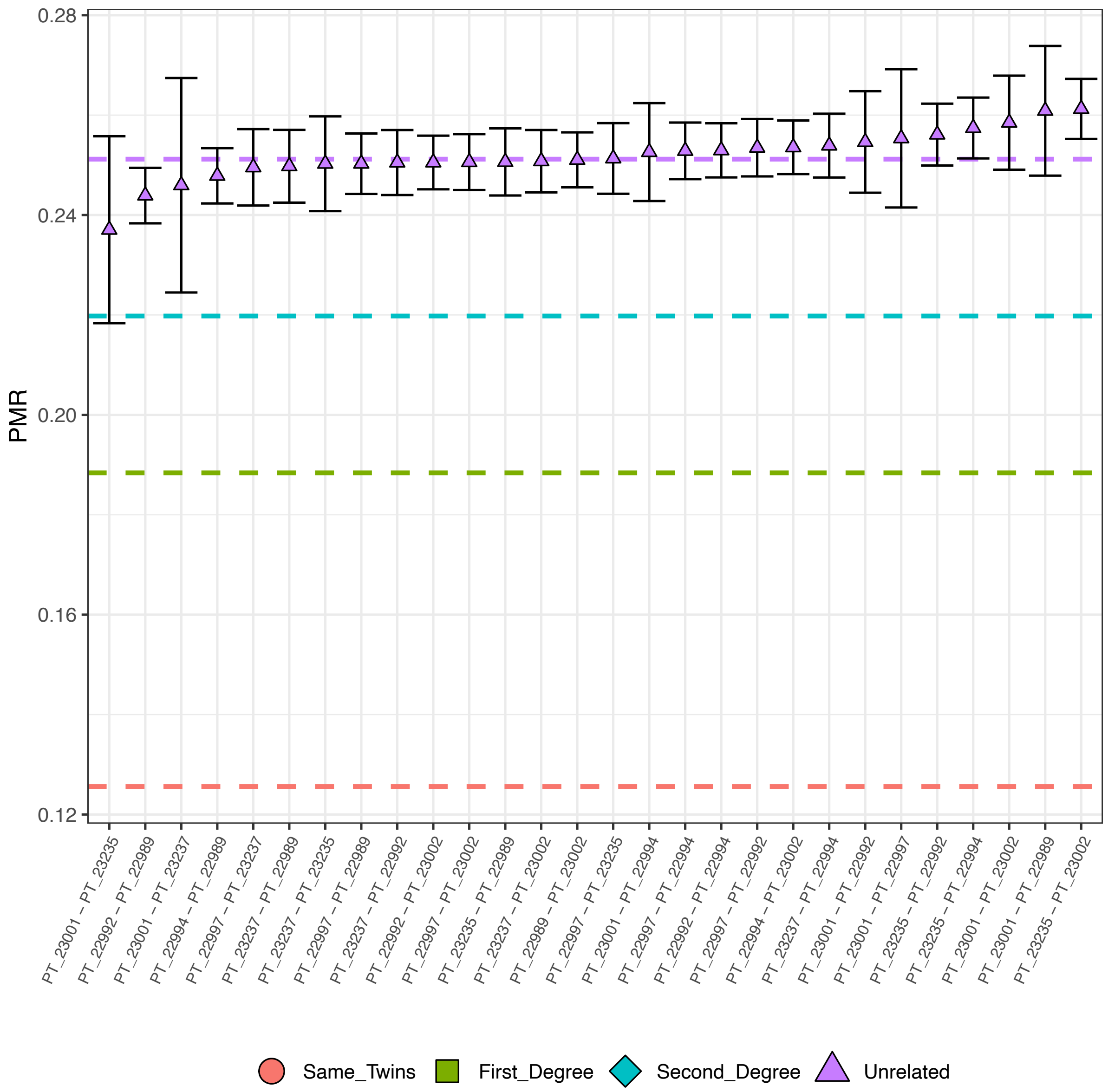

Fig. S32. Kinship analysis from Guarda_Prazo_Freixo_de_Numão (EarlyMedieval and Conquest) and Castro_de_Avelãs_Torre_Velha (EarlyMedieval and Conquest) using BREADR.

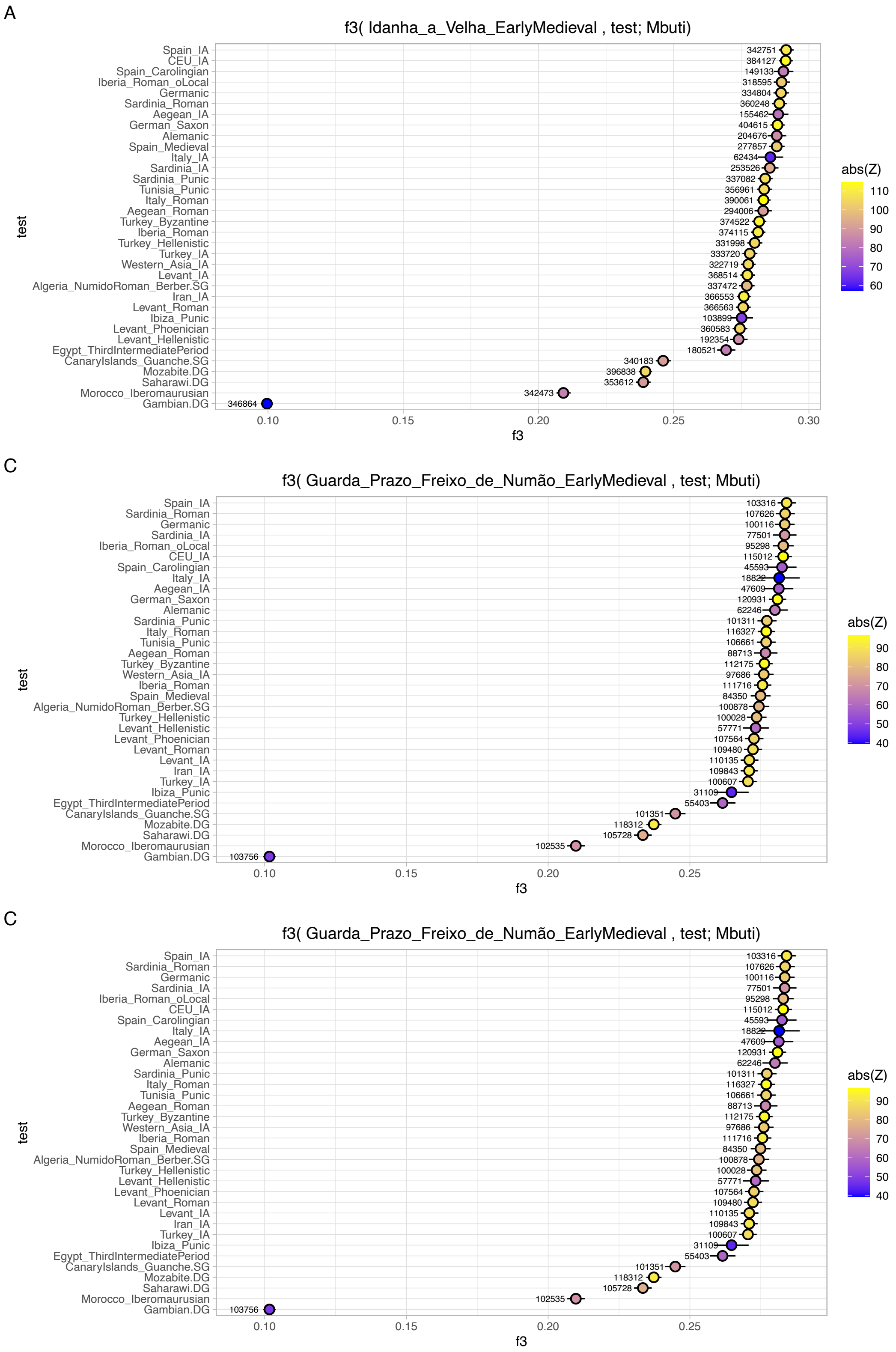

Fig. S33. The x-axes represent the f_3-statistic values, with results displayed as the mean ± 1-SD, and colors representing Z-scores. The numbers above each dot indicate the number of SNPs used for each calculation. Outgroup f_3-statistics are presented as f_3(X, Test; Chimp) with *Test* including Eurasian and African populations from the Early Medieval period or proxies and *X* being (A) Idanha_a_Velha_EarlyMedieval, (B) Guarda_Prazo_Freixo_de_Numão_EarlyMedieval and (C) Castro_de_Avelãs_Torre_Velha_EarlyMedieval.

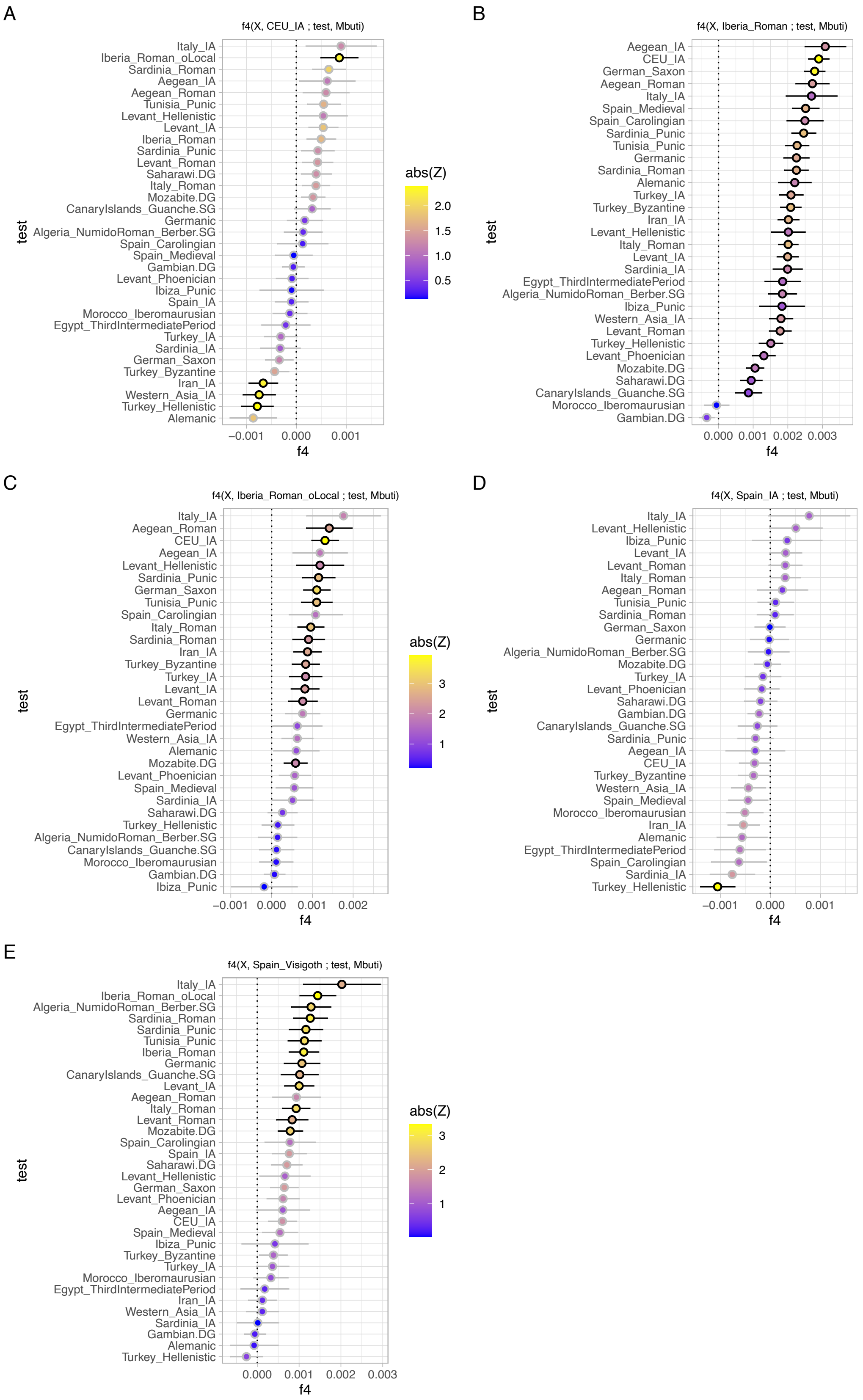

Fig. S34. The x-axes represent the f_4-statistic values, with results displayed as the mean ± 1-SD, colors representing Z-scores and black strokes Z-scores > 2. f_4-statistics are shown as f_4(X, Y; Test, Mbuti) with *Test* including Eurasian and African populations from the Visigoth period or proxies, *X* being Idanha_a_Velha_EarlyMedieval and *Y* being (A) CEU_IA, (B) Iberia_Roman, (C) Iberia_Roman_oLocal, (D) Spain_IA, (E) Spain_Visigoth.

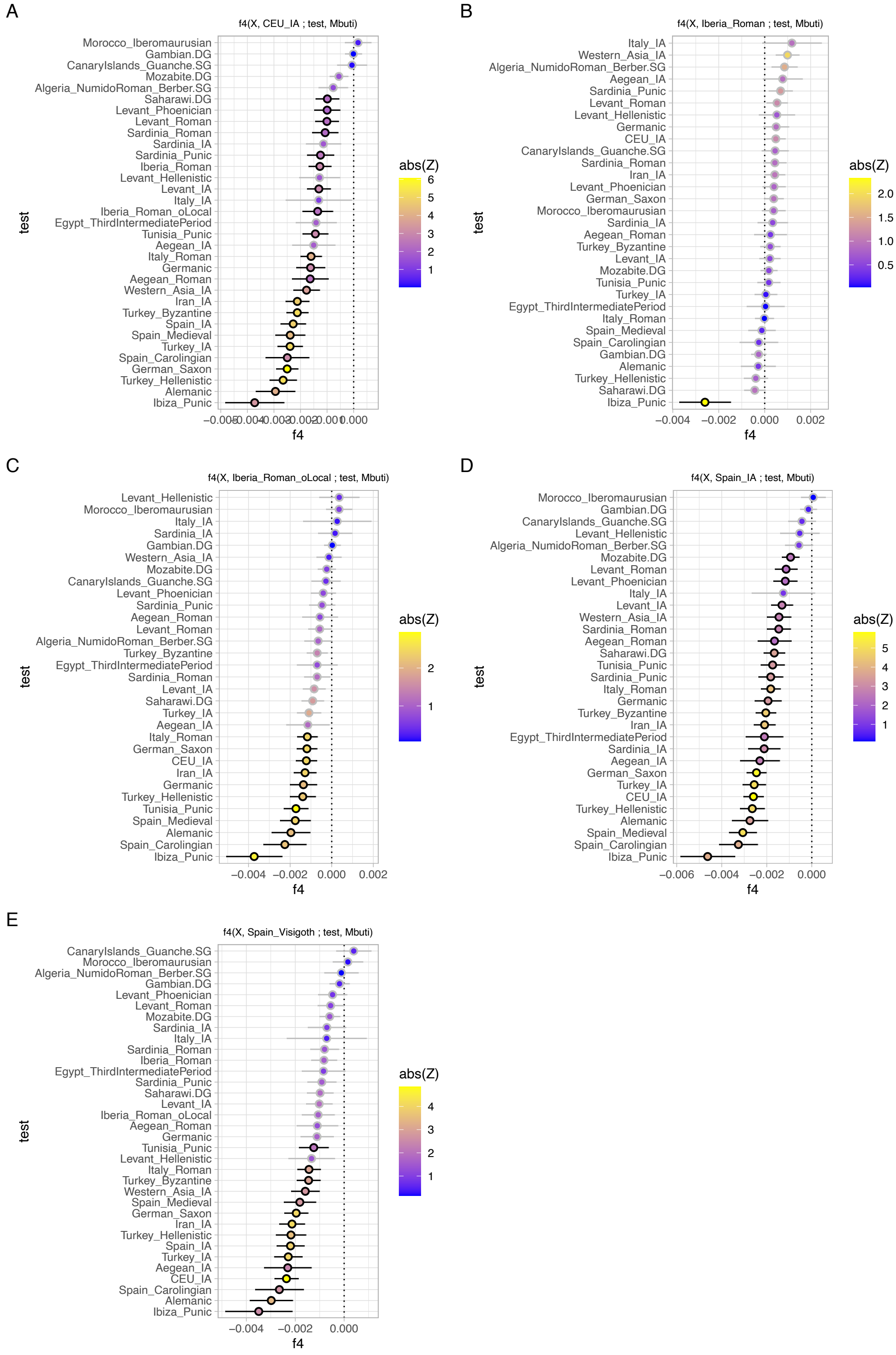

Fig. S35. The x-axes represent the f_4-statistic values, with results displayed as the mean ± 1-SD, colors representing Z-scores and black strokes Z-scores > 2. f_4-statistics are shown as f_4(X, Y; Test, Mbuti) with *X* being Guarda_Prazo_Freixo_de_Numão_EarlyMedieval, *Test* including Eurasian and African populations from the Visigoth period or proxies and *Y* being (A) CEU_IA, (B) Iberia_Roman, (C) Iberia_Roman_oLocal, (D) Spain_IA, (E) Spain_Visigoth.

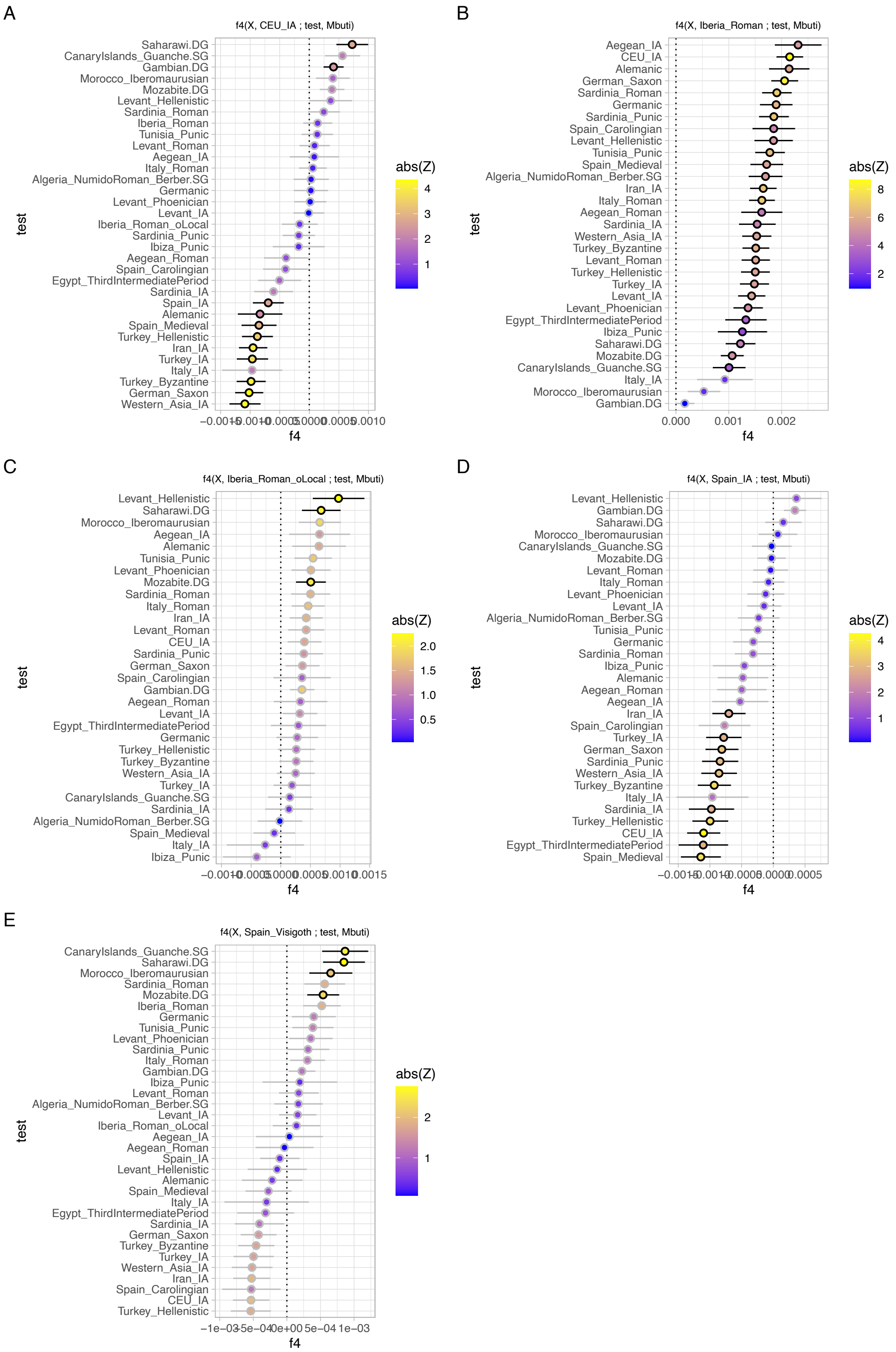

Fig. S36. The x-axes represent the f_4-statistic values, with results displayed as the mean ± 1-SD, colors representing Z-scores and black strokes Z-scores > 2. f_4-statistics are shown as f_4(X, Y; Test, Mbuti) with *X* being Castro_de_Avelãs_Torre_Velha_EarlyMedieval, *Test* including Eurasian and African populations from the Visigoth period or proxies and *Y* being (A) CEU_IA, (B) Iberia_Roman, (C) Iberia_Roman_oLocal, (D) Spain_IA, (E) Spain_Visigoth.

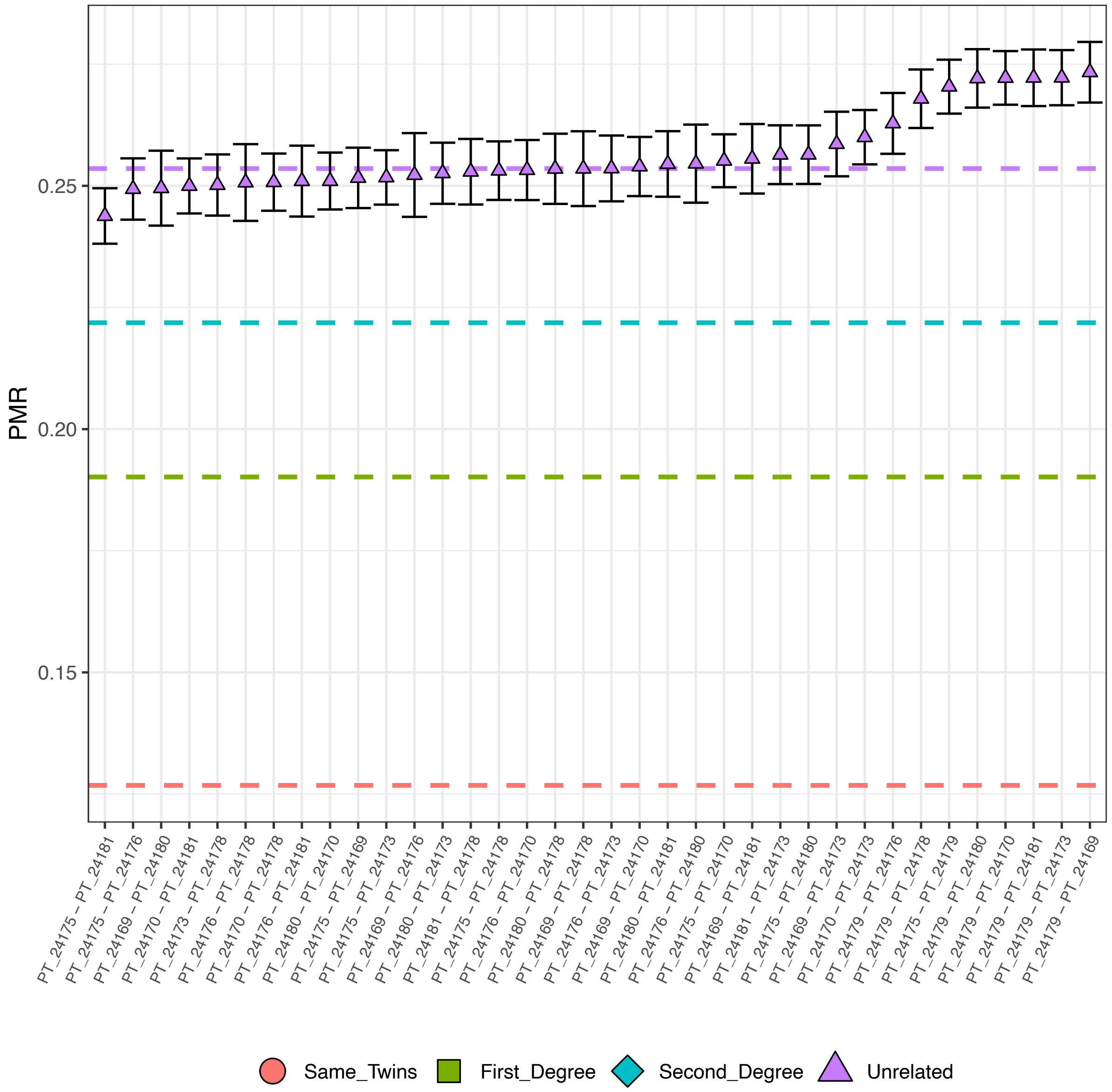

Fig. S37. Kinship analysis from Islamic Loulé and Santarém using BREADR.

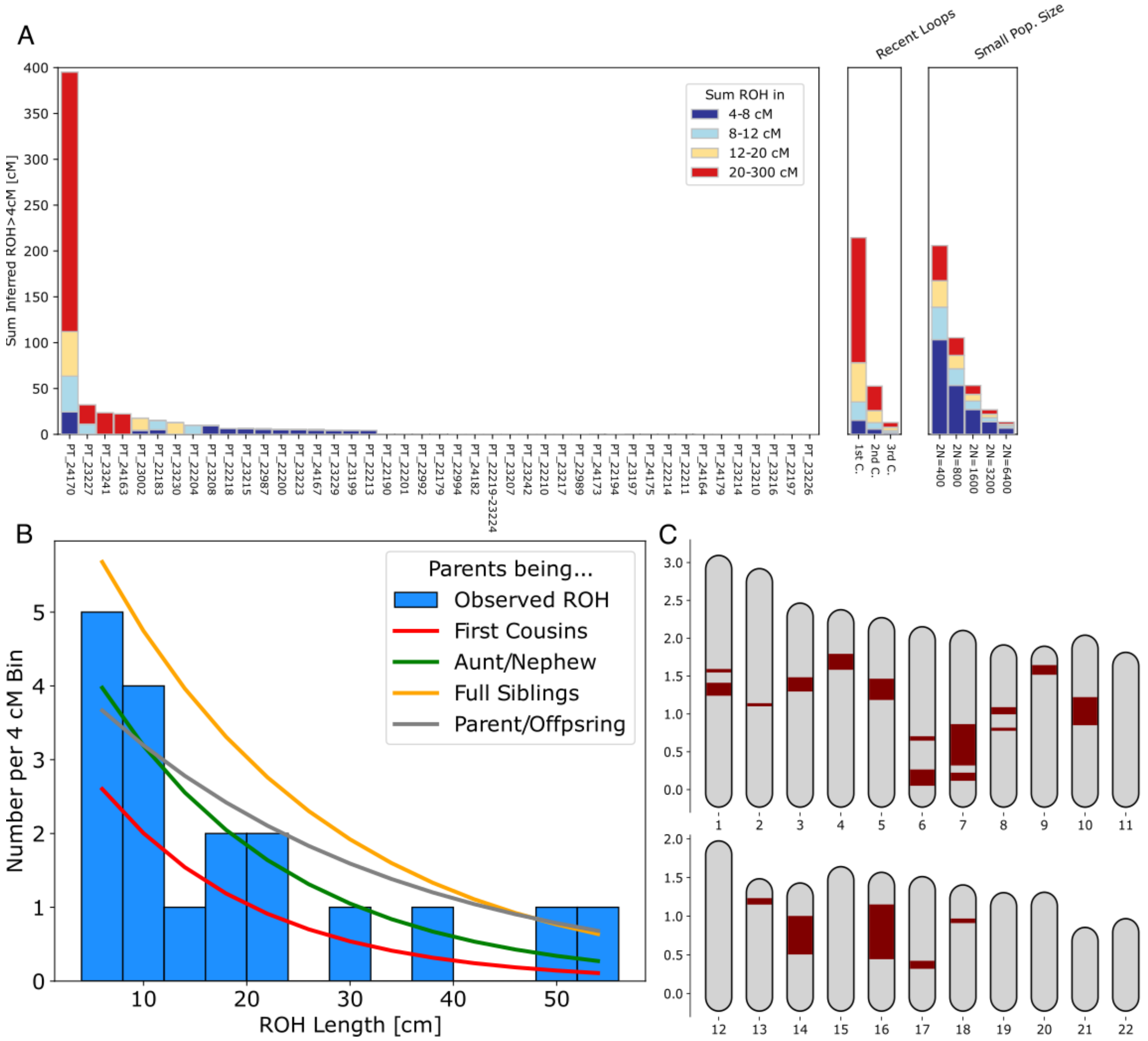

Fig. S38. (A) ROH analysis for individuals from the present study, exhibiting cumulative ROH lengths exceeding 4 cM and coverage of more than 400,000 SNPs from the 1240k dataset. (B) ROH lengths and expected densities of ROH for certain degrees of parental relationships and (C) karyotype for PT_24170 from Santarém_Islamic.

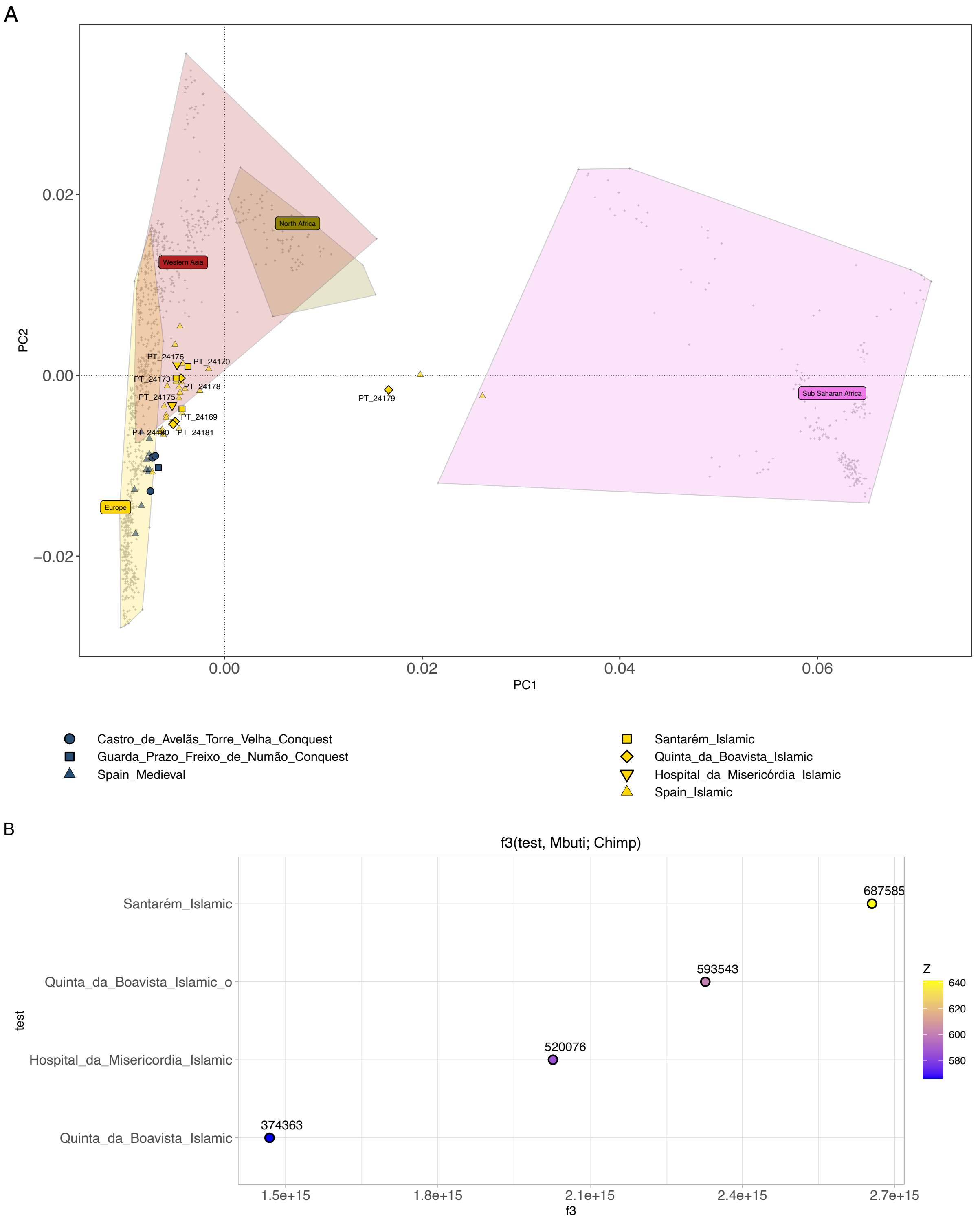

Fig. S39. (A) PCA of present-day West Eurasians, North Africans and Sub-Saharan Africans (overlaid colored polygons represent geographical clusters) with ancient individuals from Iberia and other regions projected onto the first two principal components, focusing on the Islamic period. Colors correspond to different temporal periods, as shown in 1B. (B) Outgroup f_3-statistics are presented as f_3(Test, Mbuti; Chimp) with *Test* being Hospital_da_Misericordia_Islamic, Quinta_da_Boavista_Islamic, Quinta_da_Boavista_Islamic_o and Santarém_Islamic. The x-axes represent the f_3-statistic values, with results displayed as the mean ± 1-SD, and colors representing Z-scores. The numbers above each dot indicate the number of SNPs used for each calculation.

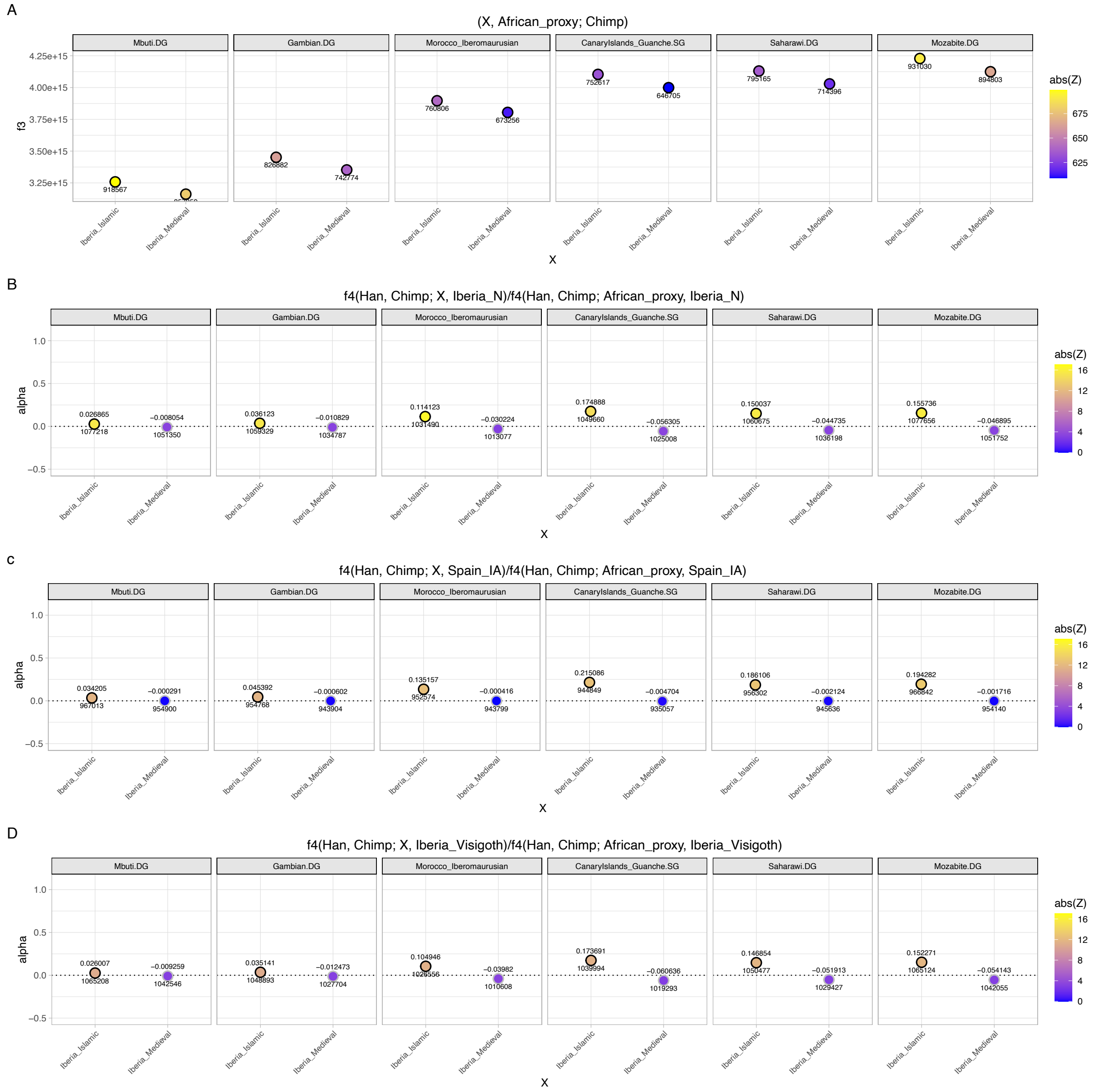

Fig. S40. (A) Outgroup f_3-statistics are presented as f_3(Iberia_Islamic/Iberia_Medieval, Test; Chimp) with *Test* including several African proxies. The y-axes represent f_3-values, with results displayed as the mean ± 1-SD, and colors representing Z-scores. The numbers below each dot indicate the number of SNPs used for each calculation. (B) f_4-ratios of the form (f_4(Han, Chimp; Iberia_Islamic/Iberia_Medieval, Y)) / (f_4(Han, Chimp; Test, Y)), where *Y* represents Iberia_N, (C) Spain_IA, and (D) Iberia_Visigoth. The y-axes represent alpha values, with results displayed as the mean ± 1-SD, and colors representing Z-scores (black strokes for Z-scores > 2 and grey strokes for Z-scores < 2). The numbers above each dot indicate the alpha values and the numbers below indicate the number of SNPs used for each calculation.

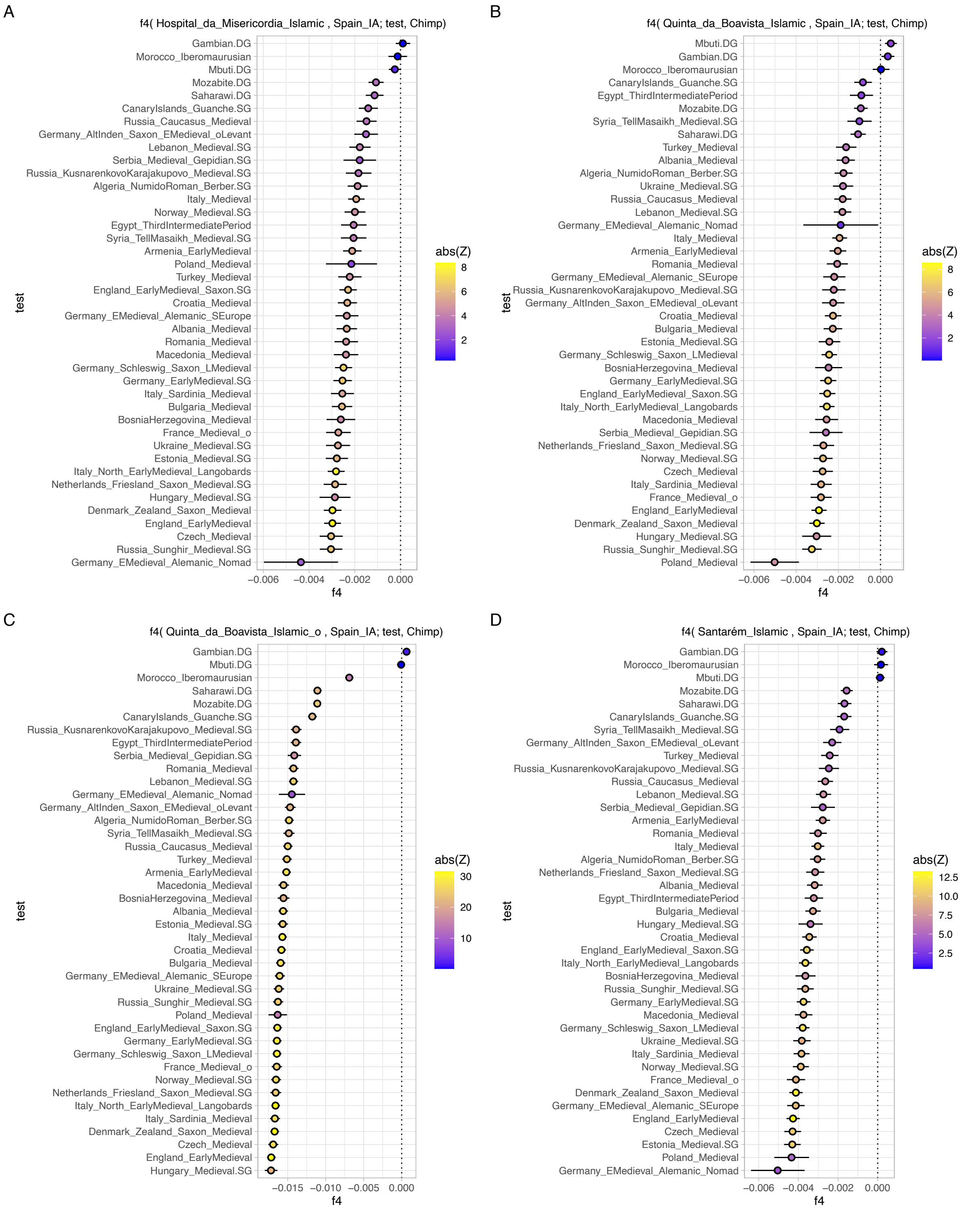

Fig. S41. The x-axes represent the f_4-statistic values, with results displayed as the mean ± 1-SD, and colors representing Z-scores. The numbers above each dot indicate the number of SNPs used for each calculation. f_4-statistics are shown as f_4(X, Spain_IA; Test, Chimp) with *Test* including Eurasian and African populations from the Islamic period or proxies and *X* being (A) Hospital_da_Misericordia_Islamic, (B) Quinta_da_Boavista_Islamic, (C) Quinta_da_Boavista_Islamic_o and (D) Santarém_Islamic.

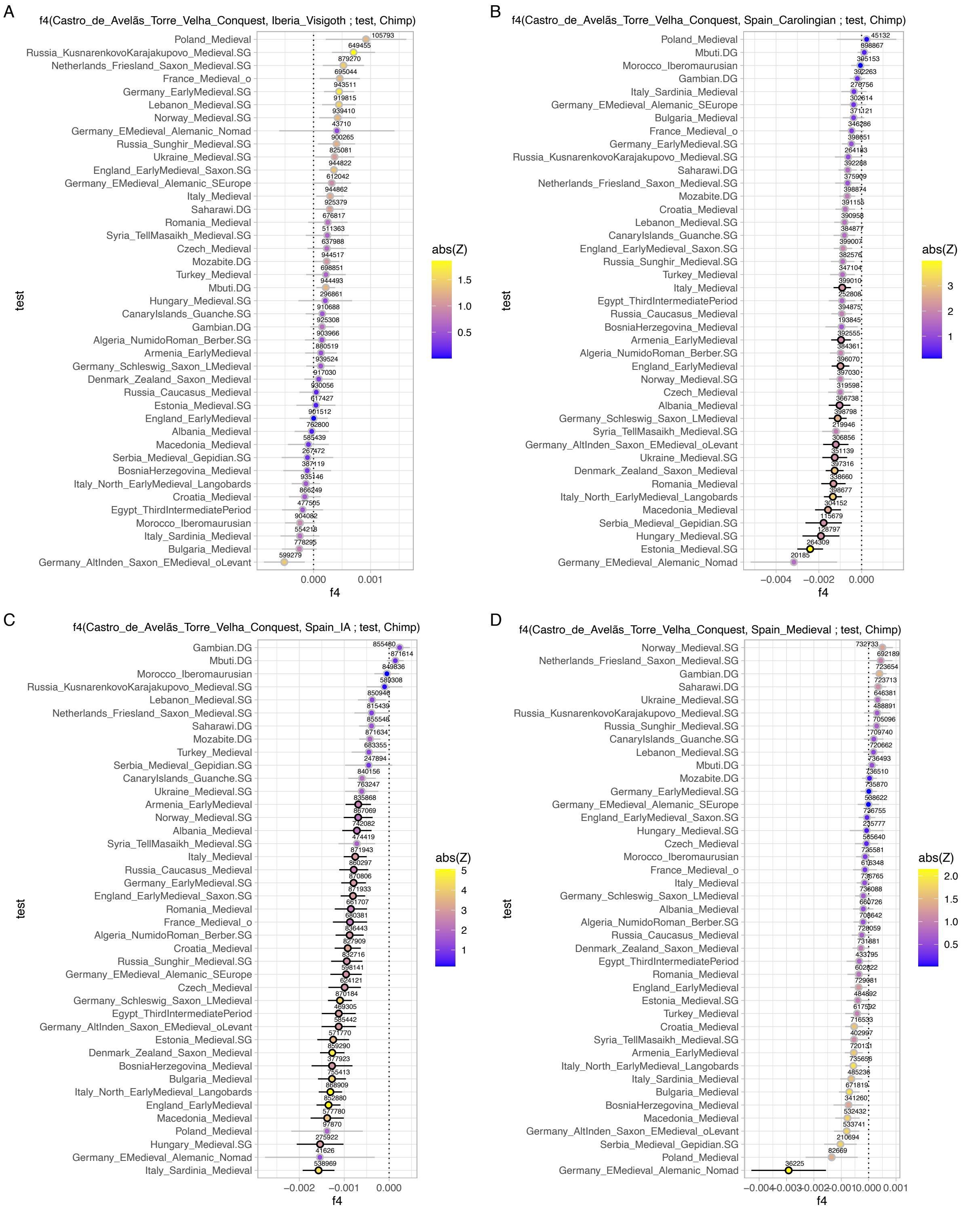

Fig. S42. The x-axes display f_4-statistic values as mean ± 1 SD, with colors indicating Z-scores and black strokes for Z-scores > 2. Numbers above each dot show the number of SNPs used. f_4-statistics are shown as f_4(Castro_de_Avelãs_Torre_Velha_Conquest, X; Test, Mbuti) with *Test* including Eurasian and African populations from the Medieval period or proxies and *X* being (A) Iberia_Visigoth, (B) Spain_Carolingian, (C) Spain_IA, and (D) Spain_Medieval.

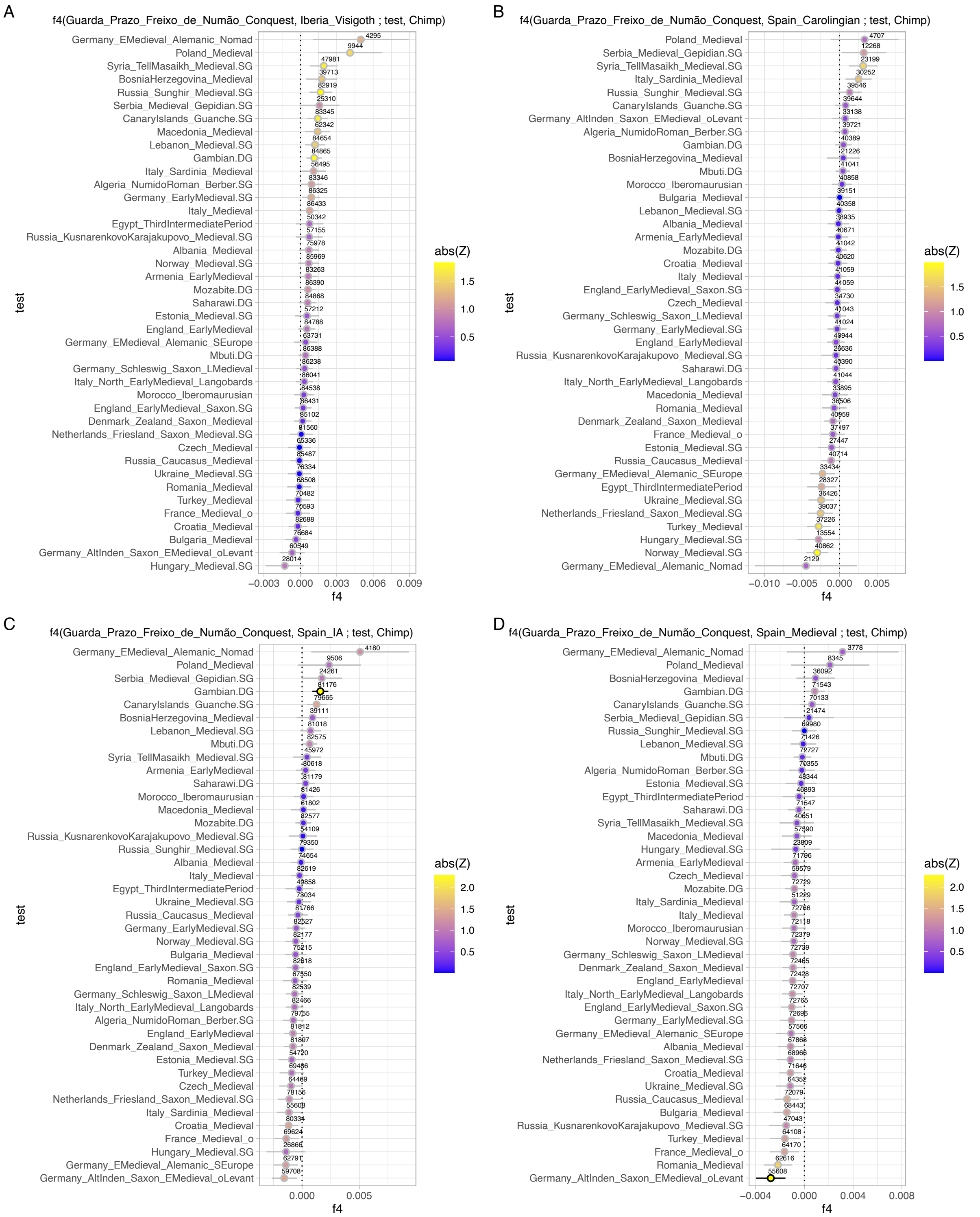

Fig. S43. The x-axes represent the f_4-statistic values, with results displayed as the mean ± 1-SD, colors representing Z-scores and black strokes Z-scores > 2. The numbers above each dot indicate the number of SNPs used for each calculation. f_4-statistics are shown as f_4(Guarda_Prazo_Freixo_de_Numão_Conquista, X; Test, Mbuti) with *Test* including Eurasian and African populations from the Medieval period or proxies and *X* being (A) Iberia_Visigoth, (B) Spain_Carolingian, (C) Spain_IA and (D) Spain_Medieval.

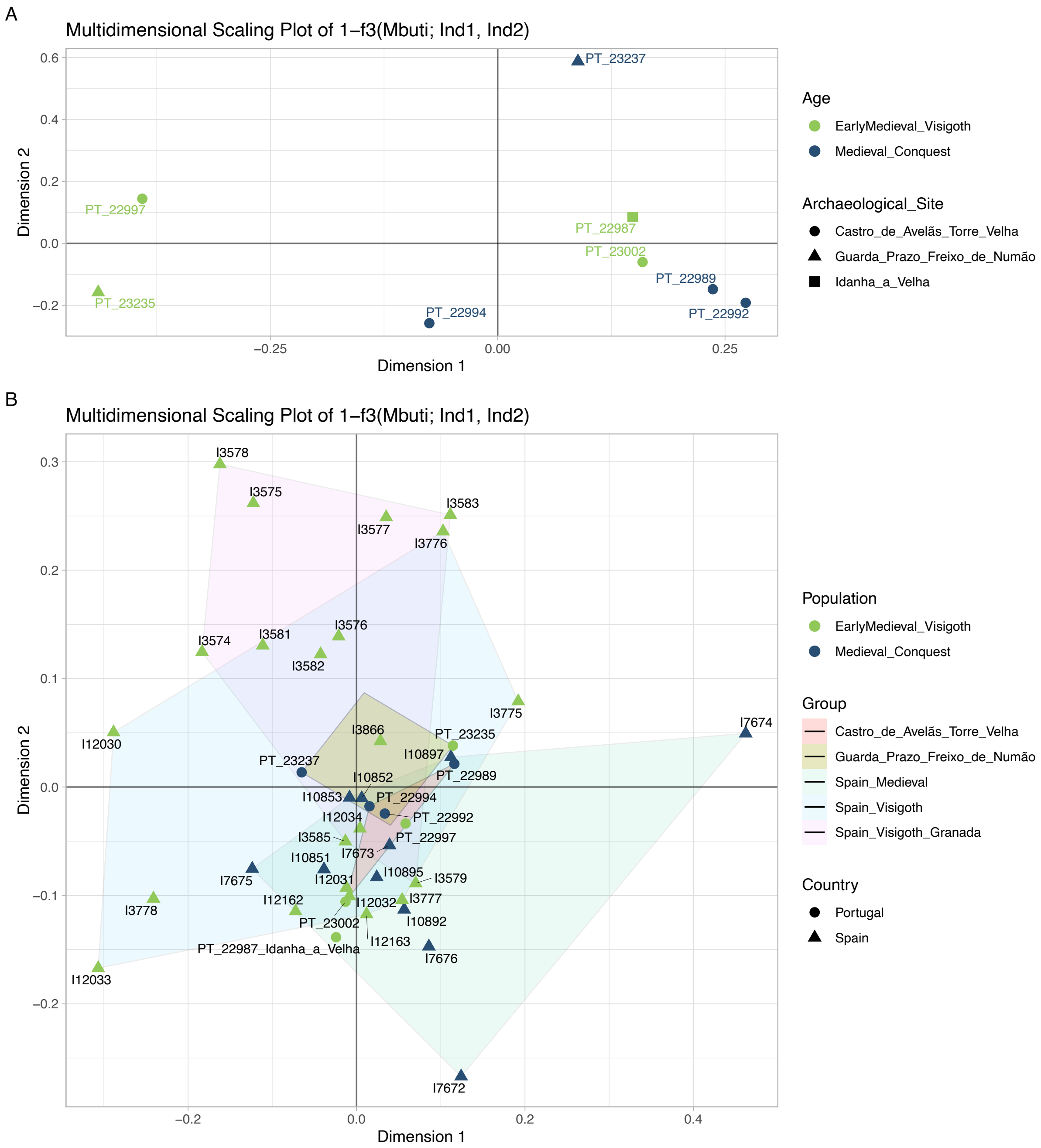

Fig. S44. Multidimensional scaling (1-f_3) for the Visigoth and Christian Conquest individuals. Outgroup pairwise f_3 computed with Mbuti as outgroup.

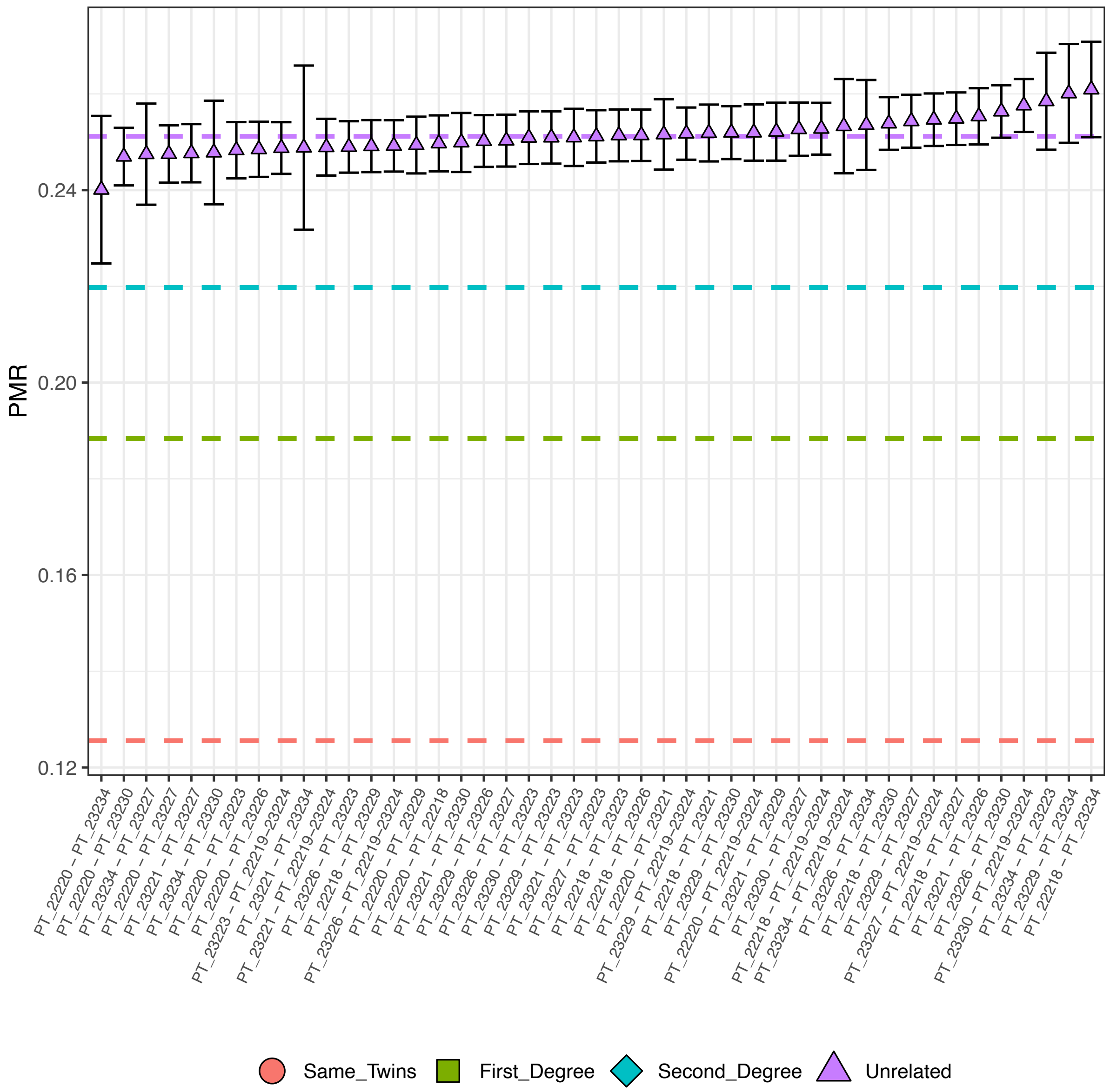

Fig. S45. Kinship analysis from Santarém_Rua_dos_Barcos_13th-c using BREADR.

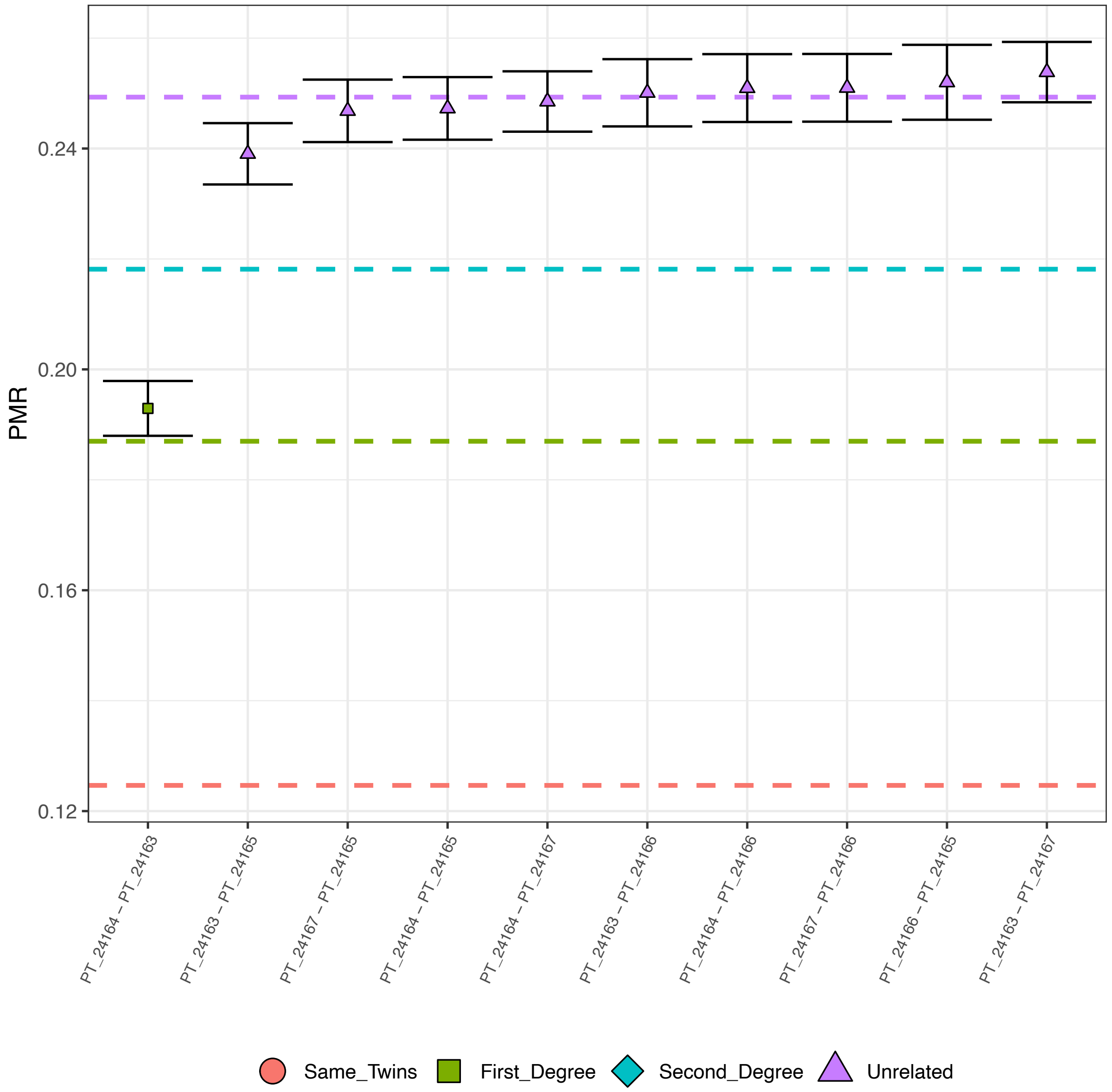

Fig. S46. Kinship analysis from São_Miguel_de_Odrinhas_13th-c using BREADR.

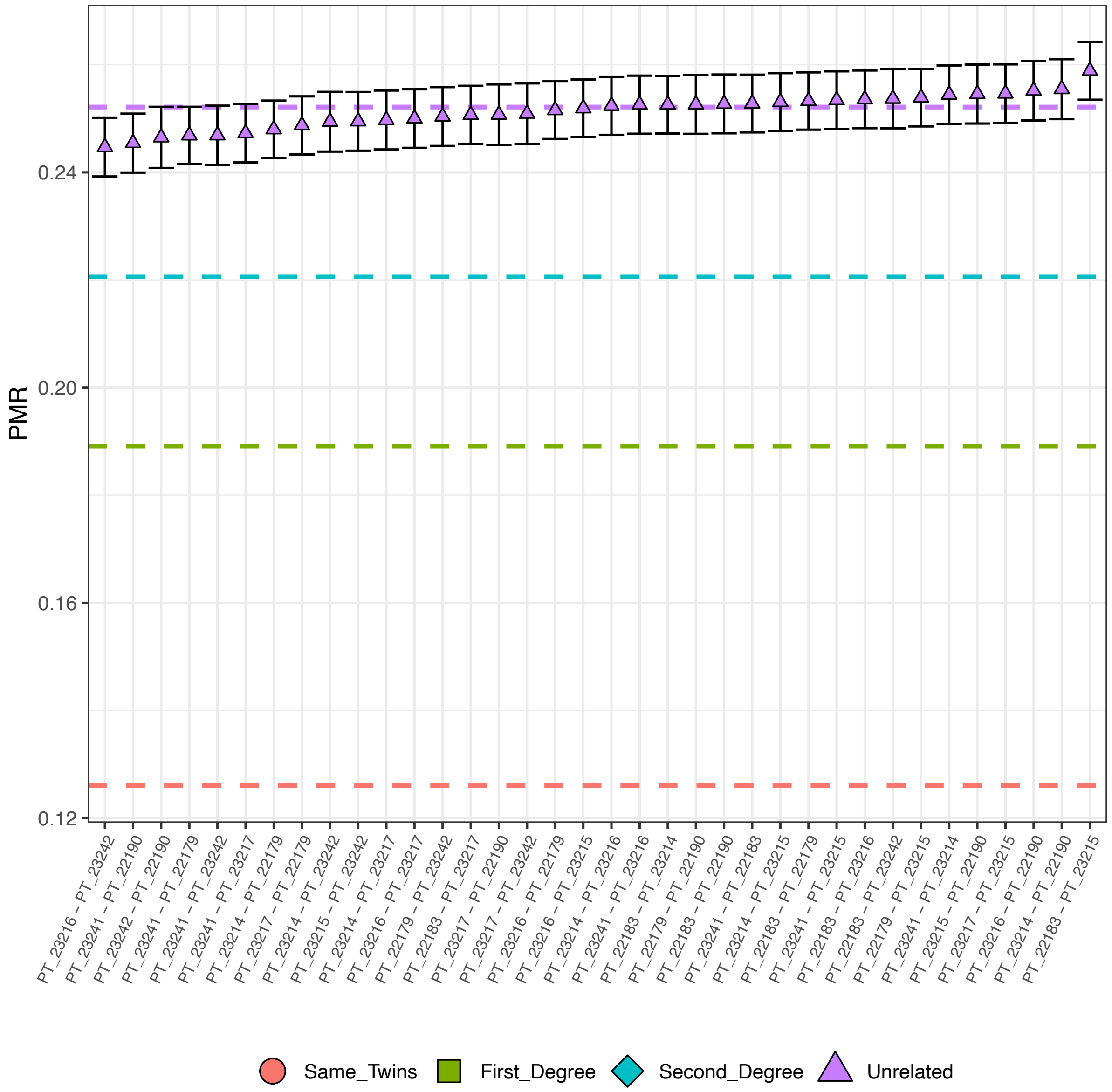

Fig. S47. Kinship analysis from Castelo_de_Montemor_o_Velho_18th-c, Aveiro_Travanca_18th-c, and Castelo_de_Abrantes_19th-c using BREADR.

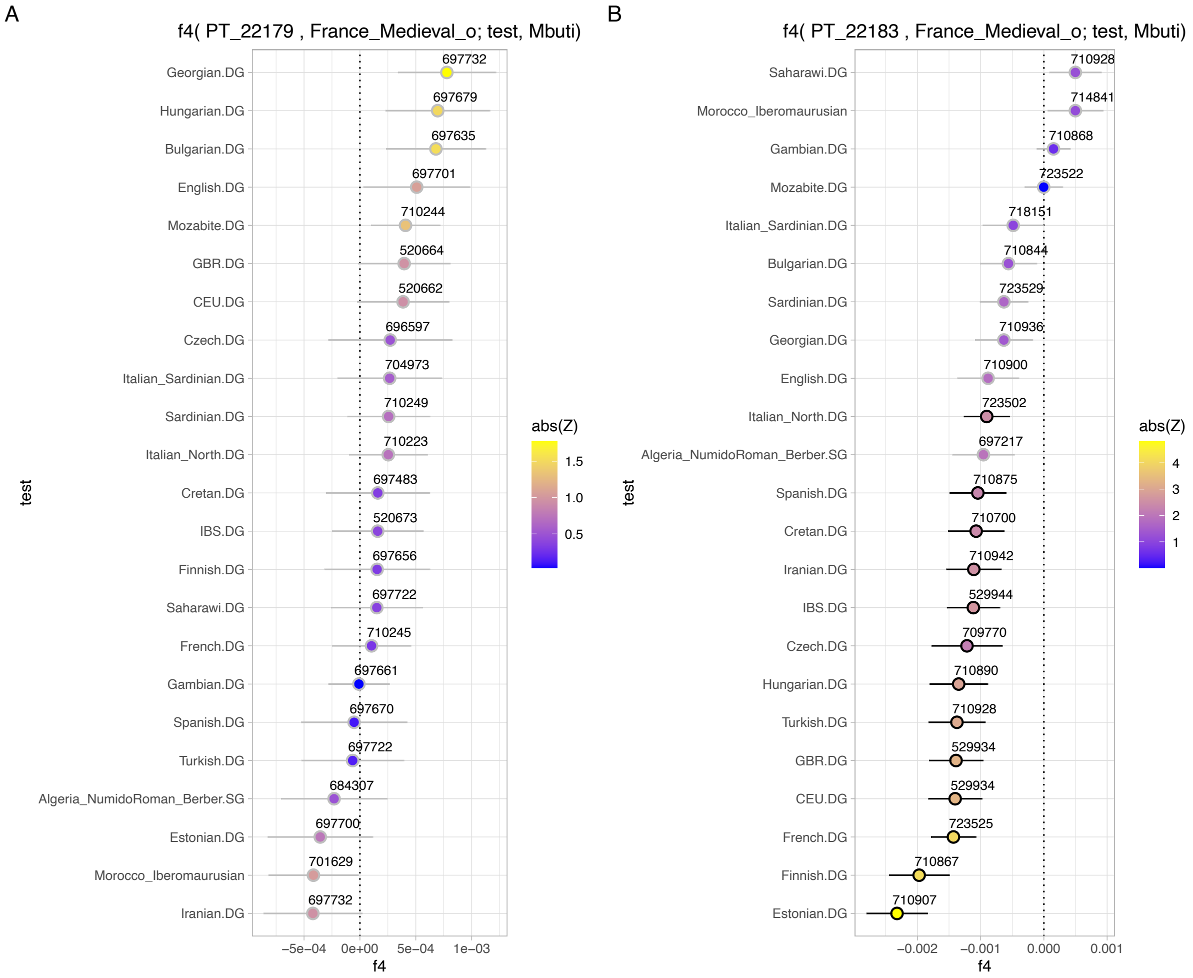

Fig. S48. The x-axes represent the f_4-statistic values, with results displayed as the mean ± 1-SD, colors representing Z-scores and black strokes Z-scores > 2. The numbers above each dot indicate the number of SNPs used for each calculation. f_4-statistics are shown as f_4(X, France_Medieval_o; Test, Mbuti) with *Test* including Eurasian and African populations from the Medieval period or proxies and *X* being (A) PT_22179 and (B) PT_22183.
